# Virus Capsid Modifications Accompanying Inactivation during Iron Electrocoagulation Revealed by Proteomics, Infrared Spectroscopy, and Molecular Modeling

**DOI:** 10.1021/acs.est.5c13277

**Published:** 2025-12-31

**Authors:** Akshat Verma, Shankararaman Chellam

**Affiliations:** † Department of Civil & Environmental Engineering, 14736 Texas A&M University, College Station, Texas 77843-3136, United States; ‡ Department of Chemical Engineering, Texas A&M University, College Station, Texas 77843-3122, United States

**Keywords:** MS2 bacteriophage, MALDI-TOF, spatial interaction mapping, DFT, potable reuse, Fenton reactions, water/wastewater treatment

## Abstract

Society’s drinking water needs are increasingly met by reusing municipal wastewater, necessitating high virus Log_10_ Reduction Values (LRVs). Concurrently, electrified processes are gaining prominence for water/wastewater treatment. Herein, we report that electrocoagulation with a low-carbon steel anode and graphite cathode at pH 6.5 and 5.5 and iron dose of 20 mg/L reduced the MS2 coliphage below detection limits (LRVs **≳**6.7) in just 11.5 min. Matrix Assisted Laser Desorption Ionization-Time-of-Flight Mass Spectrometry (MALDI-TOF-MS) of electrocoagulated viruses revealed single, double, and triple oxygen adducts and a negative mass shift peak in its coat protein without cleavage/scission. Density functional theory calculations coupled with computational spatial interaction mapping evidenced the formation of the quintet ferryl ion-cysteine 46 cluster. Hence, inactivation appears to have been accompanied by nonproteolytic capsid damages induced by reactive oxygen species (ROS). Evidence also pointed to possible ROS interactions with arginine 49 and tryptophan 32 residues. The $\frac{\alpha ‐helix}{\beta ‐sheet}$ ratio of viral protein secondary structures quantified by deconvoluting the amide I region of infrared spectra strongly and negatively correlated with inactivation, carbonyl group content, and MALDI-TOF-MS-derived protein alterations. Hence, iron electrocoagulation achieved high virus LRVs by removal (through enmeshment) and inactivation (via specific ROS interactions with coat protein residues).

## Graphical abstract

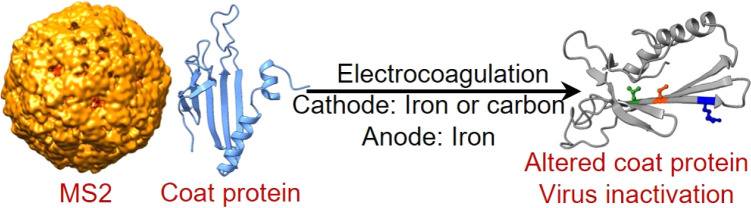

## 1 Introduction

The health and economic burden of waterborne viral illness in the United States and worldwide are high especially in children and the Global South.^1,2^ Viral risks magnify when municipal wastewater is purified for human consumption, compelling regulatory agencies to promulgate stringent Log_10_ Reduction Values (LRVs) and emphasize process engineering principles (e.g., multiple barrier approach) during (in)­direct potable reuse.^3,4^ Given our long familiarity with chemical coagulation for water/wastewater treatment, it is a logical choice to implement during potable reuse to improve contaminant removal and enhance treatment train robustness and resiliency.^3,5,6^ Its electrochemical analog “electrocoagulation” is an increasingly researched option to mitigate viruses via small-scale distributed treatment^7,8^ incorporating many advantages of electrified treatment.^9,10^

Male specific coliphages are good enteric virus surrogates in water/wastewater treatment investigations because some of them are physiologically similar to and reportedly behave like pathogens in the environment, reliably track fecal contamination, can be purified to high titers, and are nonpathogenic.^11^ For these reasons and as recommended by environmental virologists,^7,12^ experiments were performed with the coliphage MS2 that well-tracks ssRNA enteric pathogens (e.g., poliovirus, coxsackievirus, and norovirus) and facilitates comparison with numerous existing data sets.^7,8,13–19^

To date, specific viral amino acid and genomic targets likely associated with inactivation have been identified for disinfection with chlorine, UV light, singlet oxygen (^1^O_2_), and ferrate.^15–20^ In contrast, very limited information at the protein/amino acid level is available for coagulation, another microbial barrier, primarily because conventional treatment only removes nonenveloped viruses without inactivating them (i.e., no molecular alterations).^7,8,13,20^ We have imaged distorted and aggregated capsids following (electro)­coagulation, e.g., ref 8, without pursuing disassembly mechanisms.

Electrocoagulation can be evaluated within the same process envelope as in conventional drinking water treatment, which typically incorporates about 20 min of flocculation.^21^ Because wastewater recycling regulations require high virus LRVs (e.g., 12 for indirect potable reuse and 20 for direct potable reuse in California),^22,23^ assessing electrocoagulation performance under comparable hydraulic and regulatory design constraints is essential. Embedding these practical benchmarks within an experimental framework provides a realistic context for interpreting the mechanistic and performance results measured in the lab.

Our goals were to mechanistically investigate virus removal/inactivation during iron electro- and conventional coagulation and enhance virus LRVs by adjusting process conditions (e.g., dosage, pH, and cathode material). Primary objectives were to (i) identify protein degradation patterns and specific residues undergoing (oxidative) modifications and (ii) facilitate electro-Fenton reactions to increase LRVs by employing a graphite cathode under acidic conditions.

We prioritized coat protein degradation since it is the major capsid structural component.^17,19,24^ It is also likely more susceptible to reactive oxygen species (ROS)^17,19,25^ generated during iron electrocoagulation (i.e., ferryl ion ([Fe^IV^O]^2+^) and hydroxyl radical (^•^OH)). Capsid biochemical modifications were tracked using Matrix-Assisted Laser Desorption Ionization–Time of Flight Mass Spectrometry (MALDI-TOF-MS). Complementary information including alterations in $\frac{\alpha ‐helix}{\beta ‐sheet}$ ratio and carbonyl signatures were obtained by Fourier Transform Infrared spectroscopy (FTIR). Spatial interaction mapping^26,27^ was implemented to identify potential protein residue(s) positioned near plausible intermediates and assess their orientations. Density functional theory calculations^28–30^ were performed on protein–ROS complexes to quantify corresponding bonding characteristics, spin-state multiplicity, and electronic charge/spin polarization. To the best of our knowledge, this is the first work to integrate multiple experimental approaches with quantum mechanical modeling to demonstrate ROS-driven viral coat-protein oxidation accompanying inactivation. The combined approach provided and validated a framework to assess interactions of reactive intermediates with specific capsid residues, thereby providing clues to underlying inactivation mechanisms for the first time during electrocoagulation. Another novel contribution is that virus LRVs were enhanced by using a carbonaceous cathode during iron electrocoagulation, and underlying mechanisms were systematically examined.

## 2 Materials and Methods

### 2.1 Virus and Model Water Composition

MS2 propagation and purification followed well-established protocols, e.g., refs 8, 13, 15, 17, and 19 (details provided in Supporting Information Section S1a). The feedwater composition was tailored toward potable reuse by basing its inorganic composition on real-world secondary wastewater effluents.^31,32^ The synthetic water employed in this research contained higher levels of hardness (∼300 mg/L as CaCO_3_) and alkalinity (∼100 mg/L as CaCO_3_) compared to previous virus coagulation studies.^6–8,13,19^ It also included silica (30 mg/L) and sulfate (200 mg/L), which are often excluded from such investigations.^14–18^ The solution contained concentrations of the bicarbonate ion (∼120 mg/L) and chloride ion (300 mg/L), ions typical of secondary wastewater effluents.^33–35^ Hence, reported LRVs should be lower than measurements performed with lower concentrations (or absence) of these components^16,17,19^ because they are ROS sinks.^5,13,25,36^ The feedwater did not contain organic matter so as to avoid complications arising from their reactions with short-lived intermediates and mass spectrometric interferences during analysis of viral proteins, similar to numerous previous virus treatment publications.^8,13–20^ Additional details are in Supporting Information Section S1b and Table S1.

### 2.2 Conventional Coagulation and Electrocoagulation Experiments

All experiments were performed in triplicate, and data are reported as average ±standard deviation from three independent runs. One-hour long batch electrocoagulation experiments were performed with 500 mL water at 5, 10, and 20 mg/L total Fe, pH values of 6.5 and 5.5, and an initial virus concentration of ∼10^7^ PFU/mL (more details in Supporting Information Section S2–S4). These pH values were chosen based on earlier work demonstrating excellent virus and natural organic carbon removal by conventional iron coagulation and a practical lower pH boundary of 5.^6^ The suspension was rapidly mixed during electrolysis (coagulation) and slowly mixed for the remaining time (flocculation). Electric potential was applied at 1 mA/cm^2^ current density (Interface 1010E power supply, Gamry Instruments). Experiments performed using two identical plate electrodes (ground low-carbon steel, 99% Fe, McMaster-Carr, 1388K454, 50 cm^2^ wetted area, Table S2) were referred to as “Fe–Fe electrocoagulation.” Experiments performed with the same steel plate anode but a conductive graphite cathode (100% C, McMaster-Carr, 9121K61, Supporting Information Section S3) were termed “Fe–C electrocoagulation.” A negative control experiment was also performed where infective virus concentrations were quantified over the entire 60 min duration without passing current. More details including electrochemical reactions are in Supporting Information Section S4.

Fe­(II), total Fe, and H_2_O_2_ were monitored throughout each experiment as described in Supporting Information Section S5a. Infective virus concentrations were measured separately in the bulk solution and in the overall suspension (bulk + flocs) using the plaque assay to distinguish removal and inactivation.^7,13^ An important aspect of our research is that we distinguish between virus removal and inactivation, which cumulatively contribute to reduction during (waste)­water treatment. Removal denotes taking viruses out of the water column while retaining their infectivity, whereas inactivation represents their inability to infect their host. Both removal and inactivation are explicitly included in LRV calculations and relevant regulations (e.g., State of California’s rules for (in)­direct potable reuse^22,23^ and Environmental Protection Agency’s Surface Water Treatment Rule). Our conventional understanding is that microorganisms are only removed by coagulation–flocculation–sedimentation and filtration and only inactivated via disinfection.^37^ However, iron electrocoagulation can remove and inactivate nonenveloped viruses, thereby enhancing LRVs (i.e., process intensification by combining coagulation and disinfection in a single unit process).^7,8,13,38,39^ This is one of the main reasons for its promise and suitability for small-scale distributed treatment and our interest in this technology.

To determine total reduction (i.e., removal + inactivation), 1 mL of a well-mixed suspension was first withdrawn at predetermined times and filtered through a 0.45 μm poly­(ether sulfone) syringe filter. Then, the filtrate was immediately mixed with 25 mM Na_2_SO_3_ to quench residual oxidants and prevent further inactivation.^40,41^ Finally, the double-agar assay was performed to enumerate the infective virus concentration. To determine inactivation, a separate 0.1 mL aliquot of suspension was combined with 0.9 mL of 6% beef extract (pH 9.5) to dissolve precipitates and release captured viruses before performing the double-agar assay.^7,13^ Transient profiles of virus concentrations were obtained by normalizing the infective concentration at any time (N_t_) to the initial concentration (N_0_ ∼10^7^ PFU/mL). Each data point constituted an average of triplicate electrocoagulation experiments, with at least four plates per sample. Detection limits and detailed analytical steps are provided in Supporting Information Section S5b. Conventional coagulation was performed under the same conditions by adding FeCl_3_·6H_2_O.

### 2.3 Protein Degradation, Docking and Cluster Modeling, and Biochemical Characterization

MS2 coat protein degradation patterns were identified in linear positive ion mode using MALDI-TOF-MS^14,17,19,42^ (Bruker UltrafleXtreme). A positive control was evaluated wherein MS2 was digested with trypsin and then analyzed via MALDI-TOF-MS under conditions identical to those of intact and (electro)­coagulated viruses. This treatment is known to induce proteolytic scission^15,17,19^ unlike electrocoagulation (as reported below in Section 3.2) and masses of individual protein fragments are known. This experiment was crucial to establish our method’s sensitivity to detect low-mass fragments and make strong conclusions regarding the nature of capsid damage, i.e., proteolytic (scission of the peptide bond) or nonproteolytic (collapse/distortion of the 3-dimensional structure without peptide bond cleavage). Supporting Information Section S6 along with Figures S2 and S3 provide comprehensive information on instrument resolution, quality control, sample preparation, and the deconvolution method applied to obtain and interpret mass spectral data. Changes in protein secondary structures and carbonyl content (as measures of oxidative modifications) were monitored using ATR-FTIR^8,31,32,43,44^ (Nicolet iS10, Thermo Fisher, Supporting Information Section S8).

To identify capsid amino acid residues most susceptible to oxidative attack, a two-step modeling workflow was implemented. Most likely sites where [Fe^IV^O]^2+^ and ^•^OH^45,46^ could interact with the coat protein were first assessed at the residue level through spatial proximity mapping (AutoDock4.2.6)^26,27^ applied solely as a geometric screen to guide subsequent quantum mechanical cluster calculations. The local cluster specifically targeting the most susceptible residue at the likely reaction site was subjected to density functional theory calculations (DFT, ORCA5.0.2)^28,47^ to evaluate charge distribution and electronic interactions associated with potential oxidative modification adhering to state-of-the-art best-practices.^28^ Geometries were optimized with B3LYP-D3­(BJ)/def2-SVP­(gCP) algorithms (Becke three-parameter Lee–Yang–Parr with Grimme D3 and Becke-Johnson damping; def2 split-valence polarized with geometric counterpoise correction) and validated by a frequency check at the same level. Single-point energies and electronic analyses were refined with PBE0-D3­(BJ)/def2-TZVP subroutines (Perdew–Burke–Ernzerhof hybrid with D3­(BJ); def2 triple-ζ valence polarized), with representative def2-QZVP (quadruple-ζ valence polarized) checks to confirm basis-set convergence following well-established protocols.^28–30,46,48,49^ Implicit solvation was included at the single-point step using the Conductor-like Polarizable Continuum Model (CPCM) with the Solvation Model based on Density (SMD), parameterized for water.^28^ Spin state optimization of the ROS–coat protein cluster was performed using multiple combinations of charge and multiplicity to identify the most stable electronic configuration, with Löwdin analysis^47^ used to characterize atomic charges and spin populations, and Mayer bond order analysis^30,47^ to evaluate the covalency of coordination bonds. Supporting Information Section S7 and Figure S4 describe the preparation of receptor and reactive species structures, outlining parameterization, computational setup for spatial interaction and cluster-based modeling, analysis workflows, and associated assumptions and limitations.

## 3 Results and Discussion

### 3.1 Using a Graphite Cathode under Acidic Conditions Increased Inactivation and LRVs

The LRV sharply reached ∼3 soon after FeCl_3_ addition at pH 6.5, consistent with rapid precipitation and enmeshment by iron (oxyhydr)­oxides,^50–52^ and remained steady thereafter (Figure 1A, Supporting Information Section S9, Supporting Information Table S3).^8^ In contrast, LRVs progressively improved throughout the duration of FeCl_3_ coagulation at pH 5.5 only reaching ∼2 at 1 h (Figure 1C), attributed to the slower kinetics of iron precipitation and higher solubility of Fe­(III)-bearing solids (Supporting Information Section S9b and Figure S5).^52^ MS2 was simply removed (not inactivated) during conventional FeCl_3_ coagulation because infective viruses were quantitatively extracted from the suspension after dissolving flocs in beef extract at pH 9.5 (Figure 1B,D), as reported earlier.^7,8^

**Figure 1:**
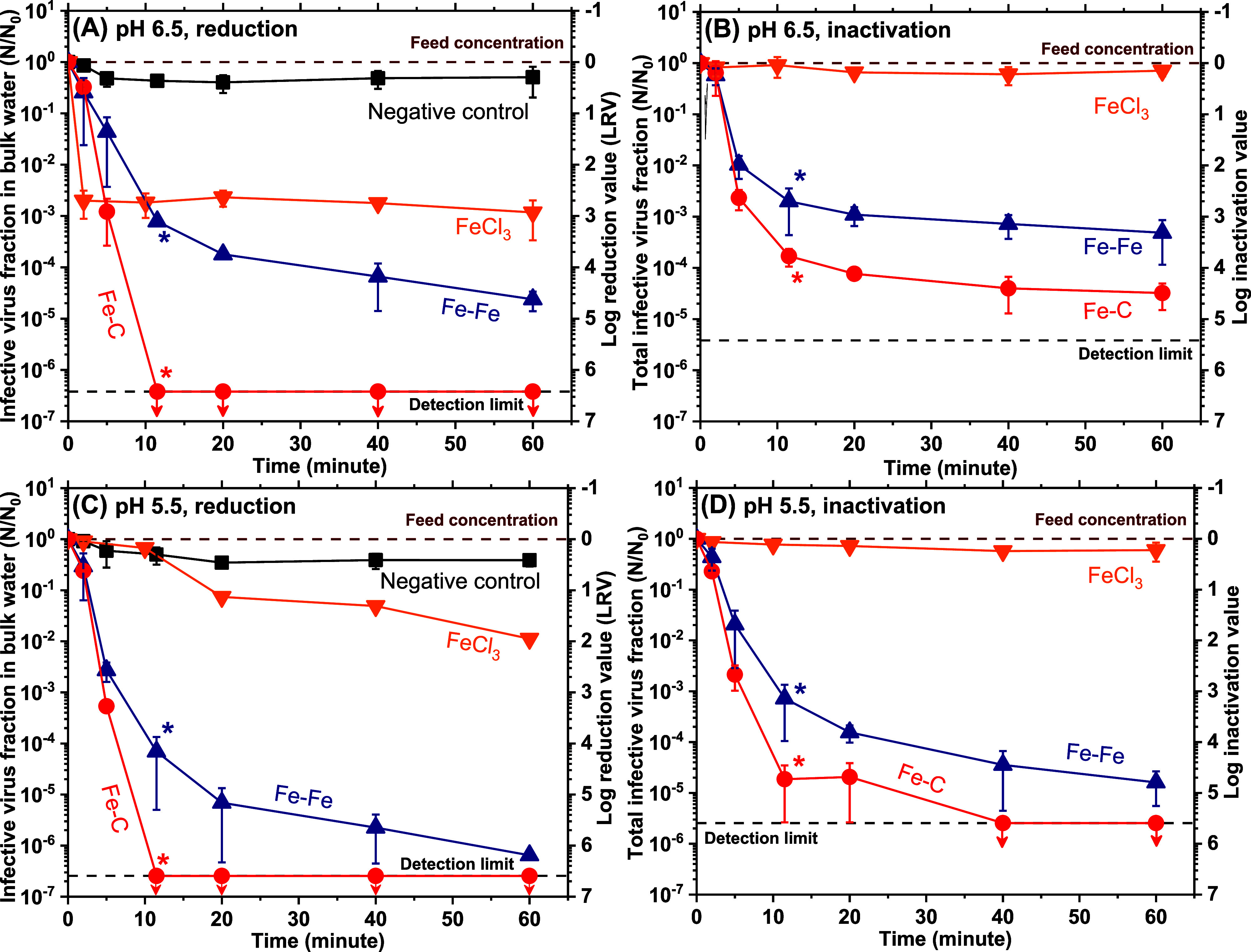
Temporal LRV profiles at pH 6.5 and 5.5 from the bulk water (a,c) and inactivation (b,d) by conventional FeCl_3_ coagulation, Fe–Fe electrocoagulation, and Fe–C electrocoagulation at a total iron dosage of 20 mg/L. Asterisks (*) denote the end of electrolysis. Data points represent the average of three independent electrocoagulation experiments. Error bars denote the corresponding standard deviation. The negative control (black color) corresponds to infectious virus concentrations measured without current passage. Results confirmed negligible reduction of viruses within the apparatus including all its constituent components. Results for 5 and 10 mg Fe/L dosages are shown in Supporting Information Figures S7 and S8.

During Fe–Fe electrocoagulation, LRVs monotonically increased, eventually reaching ∼5 (pH 6.5, Figure 1A) and ∼6 (pH 5.5, Figure 1B) in 1 h. Switching the cathode from carbon steel to graphite further improved LRVs both in terms of kinetics and magnitude, reducing MS2 to below the detection limit (LRV ≳ 6.7) in only 11.5 min (end of electrolysis, Figure 1A,C). Hence, iron electrocoagulation substantially outperformed conventional iron coagulation under all conditions and a more acidic pH with graphitic cathode further increased LRVs. Importantly, infective MS2 was only partially recovered from electrocoagulated suspensions by the plaque assay, demonstrating their inactivation (Figure 1B,D).^7,8^ A more acidic pH and a graphite cathode favored inactivation at all iron dosages (Supporting Information Figures S7 and S8). Differences in the rate and magnitude of loss of infectious viruses and the role of cathode material are pursued in more detail below.

### 3.2 Only Electrocoagulation Altered Coat Protein Biochemistry and Conformation (Not Conventional Coagulation)

#### 3.2.1 Electrocoagulation-Induced Coat Protein Degradation and Potential Oxidation Products

A complete MALDI-TOF scan of untreated MS2 revealed parent peaks at *m*/*z* 13,729 and 6,865 representing singly and doubly protonated molecular ions (M + H)^+^ and (M + 2H)^2+^, respectively, where M denotes the mass of the intact coat protein (13, 729 Da).^17,19,42^ Spectra after FeCl_3_ coagulation were indistinguishable from those of untreated viruses (Figure 2) and confirmed by peak deconvolution (Supporting Information Section S10, Table S6, and Figure S12) that assigned 100% area to the (M + H)^+^ peak. Hence, conventional iron coagulation did not modify the coat protein, consistent with our ability to quantitatively extract infective MS2 from the corresponding suspensions using the plaque assay (Figure 1B,D). This observation provided the mechanistic foundation for earlier assertions made by us, e.g., ref 8 and others, e.g., refs 7 and 13, that conventional coagulation solely removes nonenveloped viruses by enmeshment and sweep flocculation (i.e., no inactivation)^37^ and explained the underlying basis for Standard Method (SM) 9510 D to concentrate viruses albeit using alum.

**Figure 2:**
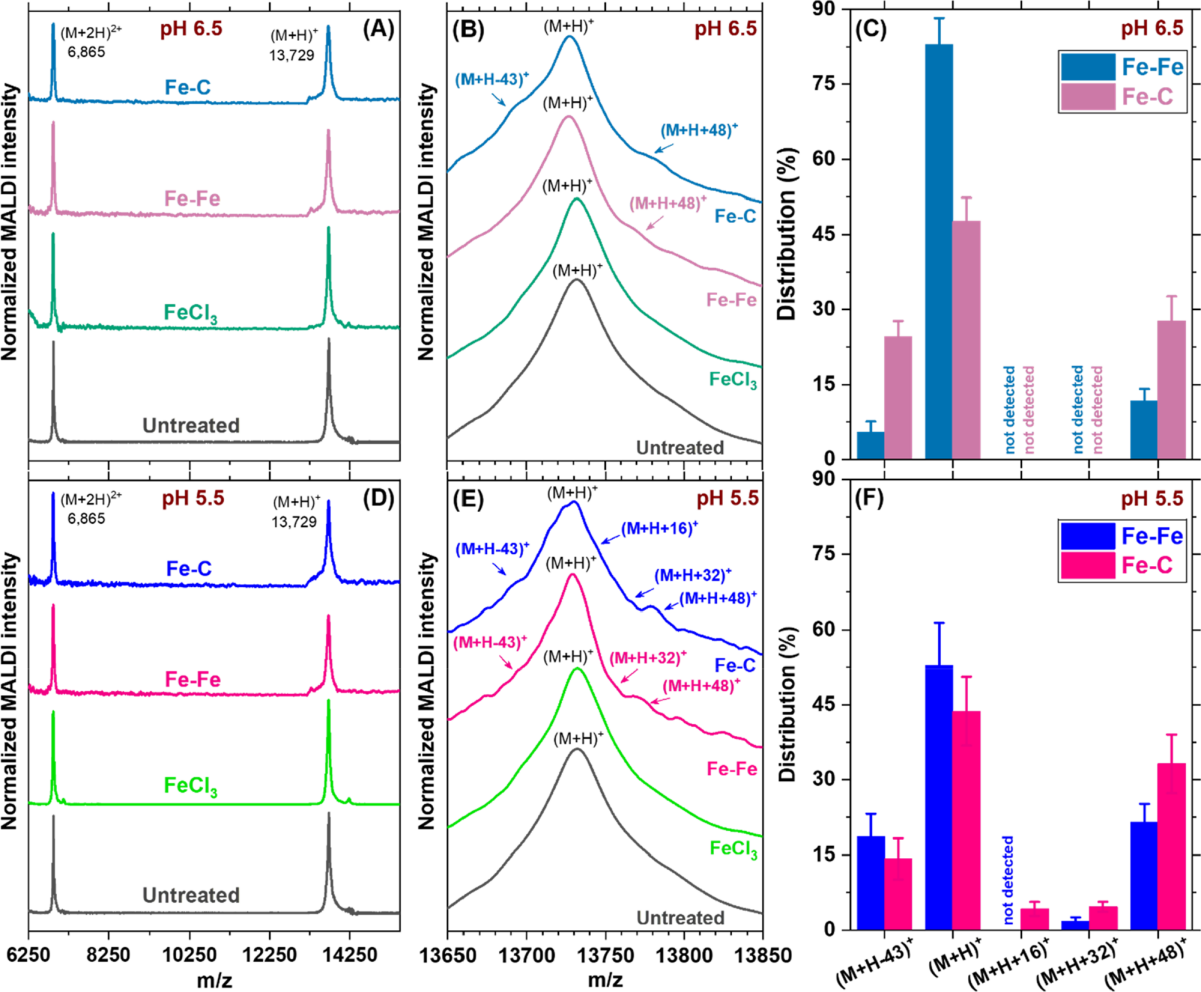
MS2 coat protein spectra before any treatment, after FeCl_3_ coagulation, after Fe–Fe electrocoagulation, and after Fe–C electrocoagulation using MALDI-TOF-MS at pH 6.5 (A–C) and 5.5 (D–F). Samples were collected after 60 min of combined coagulation and flocculation. No lower-mass cleaved products were detected below *m*/*z* 6,250 in all samples (Supporting Information Figures S9). Close-up scans of the *m*/*z* 13,729 region (B,E) revealed several mass shift peaks whose relative distribution derived after deconvolution (see Supporting Information Section S10d and Table S6) are shown in (C) and (F) for pH 6.5 and 5.5, respectively. Because only the (M + H)^+^ peak contributed to untreated and FeCl_3_-coagulated viruses, they are not depicted in (C) and (F) to improve readability (see Supporting Information Figure S12H). A trypsin-digested MS2 (positive control; Supporting Information Figure S10 and Table S5) confirmed that peptide fragments were detectable under identical MALDI conditions. Panels A, B, D, and E show average spectra from three independent runs, and the corresponding individual replicate spectra are presented in Supporting Information Figure S11. Panels C and F summarize the average with error bars corresponding to standard deviations of relative peak intensities obtained after deconvolution of triplicate spectra. Replicate spectra were highly consistent, with correlation coefficients 0.95 ≤ *r* ≤ 0.99. These details and a comprehensive statistical analysis are in Supporting Information Section S6 and S10.

No other lower-mass cleaved products or truncated proteins were detected in MALDI-TOF spectra after Fe–Fe and Fe–C electrocoagulation (Figure 2A,D and Supporting Information Figure S9) even though both approaches inactivated MS2 (Figure 1B,D), ruling out coat protein cleavage or scission. Hence, capsid integrity loss leading to inactivation as imaged by us^8^ most probably resulted from nonproteolytic disassembly, i.e., destabilization of intersubunit contacts and collapse of the quaternary capsid structure without peptide bond cleavage.^16,17^ The lack of any spectral evidence for protein truncation/cleavage suggested that electrocoagulation inactivated MS2 in a manner similar to ^1^O_2_,^14,17^ i.e., through oxidative capsid modifications. Results from the positive control experiment (Section 2.3) revealed numerous discrete peaks in the range *m*/*z* 500–6,000, consistent with the predicted tryptic fragments of MS2’s coat protein (Supporting Information Section S10b, Table S5, Figure S10). This confirmed that the instrument had sufficient sensitivity to detect peptide-bond scission when it occurred and validated the interpretation that iron electrocoagulation caused nonproteolytic, rather than cleavage-based, damage in the mass spectra shown in Figure 2.

Electrocoagulation-modified coat protein loci are marked in Figure 2B,E (also Supporting Information Section S10c and Figure S11).^17^ Although electrocoagulation induced significant inactivation (see Figure 1B,D), the (M + H)^+^ peaks remained dominant (with comparatively weaker oxygen adduct peaks), suggesting modification of only a few reactive residues in the coat protein. Peaks at (M + H + 16)^+^, (M + H + 32)^+^, and (M + H + 48)^+^ indicated corresponding single, double, and triple oxygen adducts, the first two only appearing at pH 5.5. The negative mass shift peak at (M + H – 43)^+^ is discussed in the next paragraph. As quantified in Figure 2C,F, Fe–Fe electrocoagulation retained more of the parent (M + H)^+^ peak at pH 6.5 (∼80%) than at pH 5.5 (∼50%). The parent peak was more heavily damaged during Fe–C electrocoagulation, retaining only ∼50% and ∼40% of its original area at pH 6.5 and 5.5, respectively. Hence, capsids were altered to a greater extent by lowering the pH and/or using a graphite cathode mirroring inactivation measurements. For example, (i) reducing the pH to 5.5 improved inactivation by ∼1.5-log at 60 min for Fe–Fe electrocoagulation (∼4.9-log in Figure 1D compared to only ∼3.4-log at pH 6.5 in Figure 1B) and (ii) changing the cathode material to graphite improved inactivation by ∼1.4-log at pH 6.5 and 60 min (∼4.8-log compared to only ∼3.4-log for the steel cathode, Figure 1B). Continued dominance of the (M + H)^+^ peak even for electrocoagulated viruses indicated that even oxidation of a small number of susceptible residues was sufficient to disrupt capsid stability and lose infectivity. Note that Fe–C electrocoagulation reduced the (M + H)^+^ peak area to a greater extent compared with Fe–Fe electrocoagulation and simultaneously generated more intense +16, +32, and +48 Da oxygen-adduct signals for both pH values. Together, the larger decrease in the native (M + H)^+^ peak and the stronger oxygen-adduct signatures under Fe–C electrocoagulation quantitatively corresponded to its greater MS2 inactivation shown in Figure 1. Multiple peptide transformations absent fragmentation indicated disruption of side-chains, noncovalent interactions (e.g., hydrogen bonding, electrostatic, and hydrophobic interactions), and destabilization of intersubunit interfaces and quaternary structures,^16,17,24^ leading to nonproteolytic modifications, consistent with our micrographs.^8^ These oxidative side-chain modifications weakened capsid packing and were sufficient to inactivate MS2 even when its peptide backbone remained intact.^16,17^ More intense oxidation patterns quantified during Fe–C electrocoagulation relative to the Fe–Fe system were therefore consistent with its higher LRVs under otherwise identical conditions (Figure 1). It also confirmed that nonproteolytic, ROS-driven oxidation was the dominant mechanism of virus control during iron electrocoagulation.

After the coat protein’s chemical transformations were categorized, potential individual amino acid targets and products were identified referencing the virology/biochemistry literature (Table 1). The *m*/*z* + 48 peak was attributed to triple oxidation of cystine (Cys) residues (C46 and C101, adding 3 oxygen atoms) to form cysteine sulfonic acids.^53^ The *m*/*z* + 16 peak probably arose from oxidation of methionine (Met, M88, and M108), Cys (C46 and C101), tryptophan (Trp, W32, and W82), tyrosine (Tyr, Y42, Y52, Y85, and Y129), and/or proline (Pro, P22, P65, P78, P93, P117, and P119) to methionine sulfoxides,^54^ sulfenic acids,^53^ hydroxy-tryptophan,^55^ 3,4 dihydroxyphenylalanine,^17^ and hydroxyproline/glutamic semialdehyde,^56^ respectively (adding a single oxygen atom). The *m*/*z* + 32 shift was potentially manifested due to oxidation of Met, Cys, and/or Trp to form methionine sulfones,^54^ cysteine sulfinic acids,^53^ and/or *N*-formylkynurenine,^55^ respectively (adding two oxygen atoms). Finally, conversion of arginine (Arg) residues (R38, R49, R56, and R83) to glutamic semialdehyde likely was responsible for the *m*/*z* - 43 peak, reflecting loss of the terminal guanidino group (C_1_H_5_N_3_) and incorporation of one oxygen atom. All residues discussed herein are ROS-susceptible^57,58^ and are summarized in Table 1.

**Table 1:** Probable Amino Acid Targets and Products Formed in Electrocoagulation^a^

peak	potential amino acid target	possible product	experimental conditions	reference
(M + H + 16)^+^	Met (M88, M108)	methionine sulfoxides	Fe–C electrocoagulation at pH 5.5	^54^
	Cys (**C46**, C101)	cysteine sulfenic acids		^53^
	Trp (**W32**, W82)	hydroxy-tryptophan		^55^
	Tyr (Y42, Y52, Y85, Y129)	3,4 dihydroxyphenylalanine		^17^
	Pro (P22, P65, P78, P93, P117, P119)	hydroxyproline		^57^
(M + H + 32)^+^	Met (M88, M108)	methionine sulphones	Fe–Fe electrocoagulation at pH 5.5 and Fe–C electrocoagulation at pH 6.5 and 5.5	^54^
	Cys (**C46**, C101)	cysteine sulfinic acid		^53^
	Trp (**W32**, W82)	*N*-formylkynurenine		^55^
(M + H + 48)^+^	Cys (**C46**, C101)	cysteine sulfonic acid	Fe–Fe and Fe–C electrocoagulation at pH 6.5 and 5.5	^53^
(M + H - 43)^+^	Arg (R38, **R49**, R56, R83)	glutamic semialdehyde	Fe–Fe and Fe–C electrocoagulation at pH 6.5 and 5.5	^56^

a Note that conventional FeCl_3_ coagulation neither modified the coat protein nor inactivated viruses, which is why only electrocoagulation results are included. Residues in bold font are the affected sites potentially undergoing nonproteolytic modifications (details in Supporting Information Section S10 and Table S8). Additional information and citations are in Supporting Information Sections S6, S7, and S10–S12.

The relative formation of [Fe^IV^O]^2+^ and ^•^OH during iron electrocoagulation (Supporting Information Section S4b) has not yet been authoritatively established even though the literature points to the more likely presence of ferryl ion under (slightly) acidic conditions used herein.^13,59–61^ ROS were not directly quantified in this work, but [Fe^IV^O]^2+^ formation at near-neutral pH is supported by previous homogeneous and electro-Fenton studies, isotopic evidence, and probe-based validations^59,60,62–65^ (more details in Supporting Information Section S4b). Whereas ^•^OH reacts broadly with most amino acids favoring structurally accessible protein hot spots,^58,66^ ferryl ion proceeds via inner-sphere and geometry- and coordination-dependent pathways,^29,30,48^ preferentially at accessible ligating residues. Therefore, we identified likely locations and specific residues that underwent reactions along with corresponding geometries by modeling structure-based interactions followed by quantum mechanical calculations (Supporting Information Section S10e) since peptide-level digestion^67,68^ was not possible. To the best of our knowledge, this is the first time such an investigation has been conducted for viral proteins in an environmental context.

#### 3.2.2 The High-Spin Ferryl Cluster Offered Clues to Oxidative Coat Protein Modifications

Molecular modeling was used to clarify the mechanistic basis of experimentally observed oxidation patterns and determine the interactions of the ferryl ion with specific amino acid residues present in the MS2 coat protein. Computational analysis complemented the experimental evidence by revealing the geometric and electronic factors that governed residue-level oxidation. Modeling provided the molecular basis for interpreting and coupling capsid modifications accompanying viral inactivation, highlighting its novelty and value. A two-step molecular-modeling workflow described in Supporting Information Section S7a was implemented. Spatial interaction mapping first located amino acids accessible to ferryl ion attack, and subsequent quantum chemical cluster calculations evaluated charge redistribution, spin states, and bond characteristics at these reactive sites.

Spatial interaction mapping (used solely as a geometric screen) positioned the ferryl ion near the N-terminal region of the coat protein (Phe4, Ser23-Cys46), ∼5.6 Å from Cys46, suggesting that it could be a plausible oxidative target^27,69,70^ (Figure 3A, Supporting Information Section S11a and Figure S13). The Cys46 residue was prioritized based on its thiol reactivity,^69,70^ its proximity to and alignment with the docked ferryl center (Figure 3A, Supporting Information Figure S13 and Table S7–S8), reactivity with high valent iron (Fe­(VI)) while inactivating MS2,^19^ and for being implicated in RNA binding.^15,17,24^ It was further examined with DFT to capture covalent oxo-iron­(IV) bonding, spin-state effects, and electronic polarization.^27,29,46–49^ Details of model parameters including grid, dispersion, and self-consistent field (SCF) criteria are in Supporting Information Section S7e.

**Figure 3:**
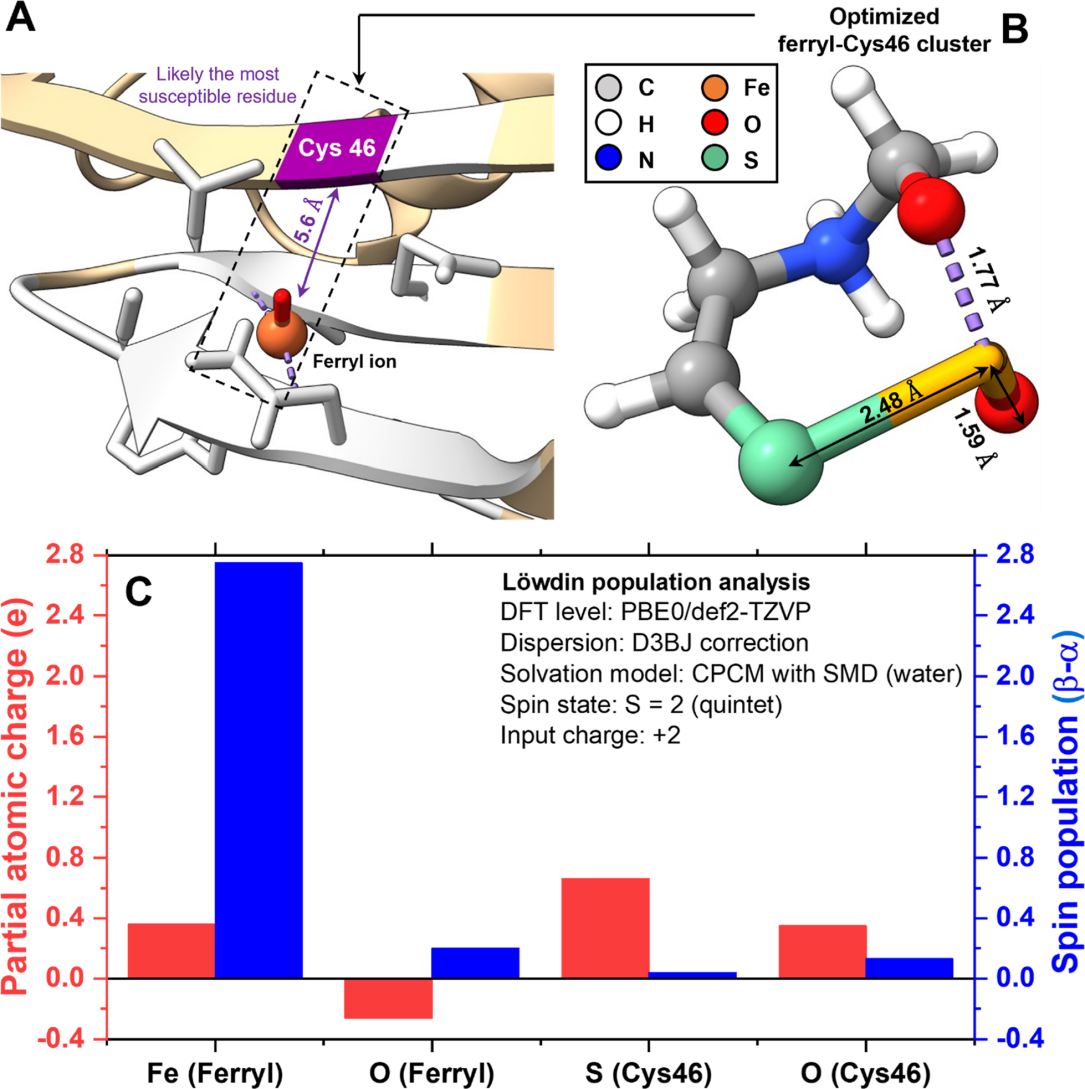
Molecular modeling providing mechanistic insights into ferryl ion-mediated Cys46 oxidation. (A) Binding pose prediction showing the ferryl ion was positioned near Cys46 (purple), along with surrounding residues (within 6 Å)^47,73,74^ shown in gray, suggesting a likely site for chemical modification. (B) Optimized quintet cluster (charge +2), showing a partial covalent Fe–S bond (2.48 Å), a short Fe–O bond (1.59 Å), and a secondary Fe···O interaction (1.77 Å, violet dashed line) with the Cys46 backbone carbonyl.^29,30^ (C) Partial atomic charges and spin populations of selected atoms in the optimized quintet cluster,^47^ consistent with a highly reactive ferryl radical capable of initiating multisite oxidation.^30,48,72^ These findings possibly support experimentally observed +16, +32, and +48 Da adducts in MALDI-TOF-MS (Figure 2). Computational reliability was ensured through convergence, spin-state, and frequency checks described in Section 2.3 and Supporting Information Sections S7 and S11.

Among the spin-state configurations evaluated for the ferryl–Cys46 cluster, the quintet was most stable and favored over the triplet (and even the doublet, assessed as a low-spin control). Supporting Information Section S11b details the optimization trajectories (Supporting Information Figure S15), spin-state energetics, and frequency validation, showing that only the quintet retained the lowest SCF energy, negligible spin contamination, chemically consistent bonding metrics, a large frontier orbital gap supporting single-reference stability, and no imaginary modes (true minimum), thereby providing a robust basis for its selection. The optimized geometry with multiplicity 5 (i.e., quintet in Figure 3B) featured a partial covalent Fe–S bond of length 2.48 Å^29^ with the thiolate sulfur of Cys46, a short terminal Fe–O bond of length 1.59 Å,^48,49,71^ and a secondary Fe···O interaction at 1.77 Å distance with the backbone carbonyl oxygen of the same residue.^29,30^ This configuration created a compact and spatially oriented reactive pocket, positioning two nucleophilic centers, viz., sulfur and backbone oxygen around the electrophilic ferryl ion, thereby facilitating orbital overlap and charge transfer, which are prerequisites for bond formation and multistep oxidation.^30,72^

Spin density and partial charge distribution (from Löwdin population analysis,^47^ Figure 3C) collectively support this arrangement. The ferryl iron carried the highest spin density (2.75) and a partial positive charge (0.36), consistent with a high-valent Fe­(IV) species in a high-spin (total cluster spin quantum number of 2), magnetically active configuration known to support Fe–O π* orbital occupancy and radical-type reactivity.^30^ The terminal oxo (0.20 spin density, −0.26 charge) also exhibited substantial unpaired electron density and mild polarization, indicative of partial oxyl radical character and an electrophilic, redox-active nature.^30^ Notably, the spin density extended very weakly onto the thiolate sulfur (only 0.04), while it carried a substantially positive charge (0.66), together indicating electron withdrawal and polarization of the Cys46 side chain. The backbone carbonyl oxygen also exhibited nonzero spin density (0.13) and a moderately positive charge (0.35), suggesting weak spin delocalization, likely due to orbital coupling with the high-spin iron center.^75^ This site, although only modestly spin-polarized, became more charge-polarized in solvent, reflecting electronic influence from the ferryl unit.^76^ Overall, the distribution supported electron withdrawal from sulfur through the Fe–S bond and Fe···O interactions (Figure 3A), consistent with covalent bonding and redox activation. This interpretation is consistent with Mayer bond order analysis^29,30^ (Supporting Information Section S11b, Supporting Information Table S9,S10; see also def2-QZVP basis set check with negligible differences). Even though we did not model oxidation kinetics, these patterns are consistent with a redox-active environment.^29^ A spin population of 0.8 on the cysteine backbone carbon reflected delocalization, while minor negative spins on adjacent atoms likely arose from polarization rather than redox activity^77,78^ (Supporting Information Table S9).

Collectively, these electronic and geometric features supported a mechanistic basis for likely stepwise oxidative modification of the Cys46 (C_3_H_7_NO_2_S) residue: first to cysteine sulfenic acid (C_3_H_7_NO_3_S; +16 Da), then to cysteine sulfinic acid (C_3_H_7_NO_4_S; +32 Da), and finally forming cysteine sulfonic acid (C_3_H_7_NO_5_S; +48 Da) through successive O atom incorporation.^53^ Stepwise O-addition explains discrete mass shifts observed experimentally in MALDI-TOF spectra (Figure 2B,E), supporting the role of the quintet-state ferryl–Cys46 complex as a plausible oxidative intermediate during MS2 inactivation. Together, spatial interaction mapping and electronic structure analysis provided complementary evidence linking ferryl-mediated oxidation of Cys46 with the +16, +32, and +48 Da adducts in the mass spectra. This cysteine oxidation is similar to UV_254_ specifically oxidizing the Cys46 side chain and targeting the adjacent Cys46–Ser47 region for cleavage in MS2.^15^ Interestingly, both ^1^O_2_^17^ and Fe­(VI)^19^ also target the same region.

To focus on the key reactive site, cluster-based modeling included only ferryl and the immediate Cys46 environment for bonding and spin analysis, omitting the broader protein context; solvent effects were treated implicitly with CPCM and SMD (water) at the single-point stage after gas-phase optimization (details in Supporting Information Sections S7e and S11b,c). The measured −43 Da mass shift in Figure 2 may also result from ROS-mediated conversion of the nearest neighboring Arg49 residue (C_6_H_14_N_4_O_2_; Supporting Information Table S8 lists the interatomic distances between reactive species and neighboring residues in the binding region) to glutamic semialdehyde (C_5_H_9_NO_3_,^56,79^ discussed in Section 3.2.3) even though these residues were not within 6 Å of reactive species in spatial interaction models (Supporting Information Section S11a). This was not pursued in greater detail because the lack of Fe–N coordination (Supporting Information Figure S13) necessitates indirect oxidation^56,79^ requiring a larger and more complex cluster, which was beyond our current modeling scope. Nonetheless, modification of Arg49, which lies in the extended RNA-binding patch,^80^ could weaken electrostatic interactions with the phosphate backbone,^56,79^ potentially leading to loss of genome encapsidation and consequent inactivation.

Spatial interaction mapping simulations with ^•^OH showed a unique interaction map with Trp32 (Supporting Information Section S11a and Figure S14) located in the coat protein’s N-terminal hydrophobic core,^24^ and its alteration may perturb local packing impacting capsid stability,^81^ leading to inactivation.

#### 3.2.3 Electrocoagulation Oxidized Capsid Proteins and Modified Their Secondary Structures

Based on MALDI-TOF-MS and modeling evidence for multiple oxidation events in the coat protein via electrocoagulation, we quantified modifications to capsid secondary structures and its carbonyl content (another measure of oxidative damage) using infrared spectroscopy.^8,82^

Differences between FTIR spectra of FeCl_3_-coagulated and untreated phages in Figure 4 were negligible, like MALDI-TOF-MS results (Figure 2), and consistent with the inability of conventional coagulation to inactivate MS2 ^7,8,13^ (Figure 1). In contrast, electrocoagulation induced measurable spectral differences, which became more prominent with the graphite cathode and/or at the lower pH (Figure 4A, see also Supporting Information Figure S16). The amide II relative peak area was ∼33% in untreated phages, which was reduced to ∼7% at pH 6.5 and ∼2% at pH 5.5 by Fe–C electrocoagulation, reflecting substantial changes to in-plane N–H bending and C–N stretching vibrations.^43^ Amide II peak areas also decreased following Fe–Fe electrocoagulation but to a lesser degree (to ∼18% at pH 6.5 and 12% at pH 5.5) and were only negligibly changed (by ∼1–2%) via FeCl_3_ coagulation (Figure 4A and Supporting Information Table S11). The amide II region is sensitive to Glu ν_as_(COO^–^),^44^ supporting modeling results of Glu31 interactions with ROS (Supporting Information Figures S13 and S14), validating changes to the protein backbone by electrocoagulation, possibly leading to inactivation.

**Figure 4:**
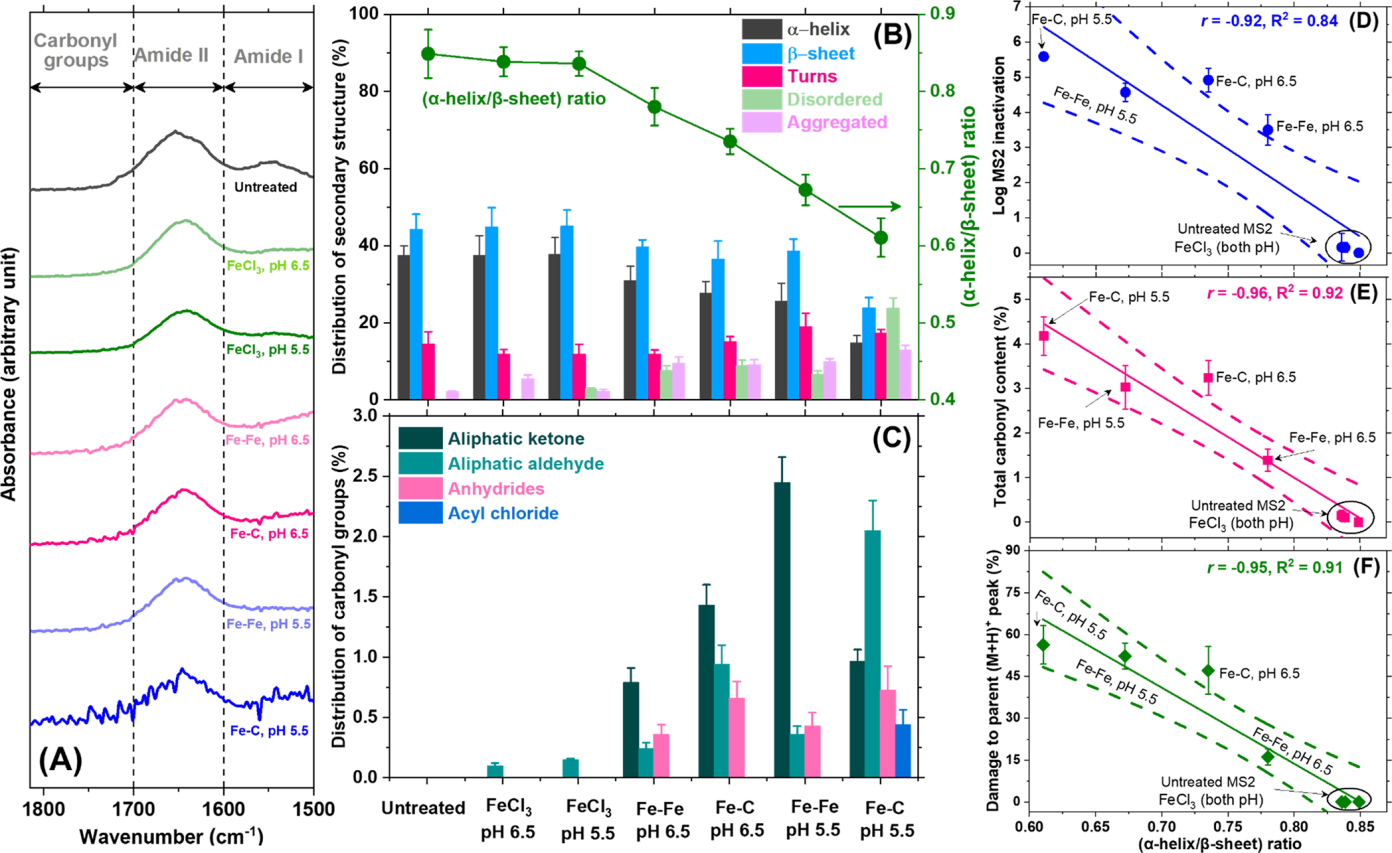
MS2 biochemical characterization before and after treatment via FeCl_3_ coagulation, Fe–Fe electrocoagulation, and Fe–C electrocoagulation at pH 6.5 and 5.5 (iron dosage of 20 mg/L at 60 min). Averages of three ATR-FTIR spectra between 1820 and 1500 cm^–1^ are in (A). Relative distribution of protein secondary structures obtained from deconvoluting the amide I region (1700–1600 cm^–1^, Supporting Information Figure S17) is in (B). The relative distribution of carbonyl-associated groups is in (C) by analyzing the 1820–1700 cm^–1^ region. Correlating α-helix/β-sheet ratio with inactivation (D), total carbonyl content (E), and MALDI-TOF derived protein damage (F). Dashed lines in D, E, and F represent 95% confidence bands. Conditions corresponding to each experiment are provided near each symbol in D, E, and F. ATR-FTIR spectra were collected in triplicate. For each of the triplicate spectral measurements, individual spectra were acquired from three different positions on the membrane used to filter suspensions containing viruses and averaged to obtain one representative spectrum per replicate, which were then averaged across replicates. Hence, each spectrum panel A represents a total of nine different spectra. The average spectra of the three independent electrocoagulation experiments covering a total of six different conditions and one control (untreated MS2) are depicted. Panels B–F show the corresponding average and standard deviation (error bars) of these triplicates for the major secondary structure and carbonyl bands. Replicate similarity was confirmed using the instrument software OMNIC QCheck correlation (*r* ≥ 0.7). Fractional contributions (Fi %) for protein secondary structure (Panel B) and carbonyl content (Panel C) were obtained from peak-fit deconvolution and are reported as average ± standard deviation for the triplicates. Detailed procedures are described in Supporting Information Section S8.

The amide I region^43^ was deconvoluted (Supporting Information Figure S17, peaks assigned per Supporting Information Tables S12 and S13) to elucidate treatment-induced changes to proteins’ secondary structures.^8^ α-Helices (∼37%) and β-sheets (∼44%) were most common in untreated MS2 with an $\frac{\alpha ‐helix}{\beta ‐sheet}$ ratio of 0.85 ± 0.03, not far from the crystallographic estimate of ∼0.75.^24^ As expected from the lack of inactivation (Figure 1) and no changes in MALDI-TOF spectra (Figure 2), adding FeCl_3_ only negligibly changed this spectral region.^8^ However, substantial alterations were noted upon electrocoagulation, particularly in the Fe–C system at lower pH (Figure 4B and Table S13I), which lowered α-helices and β-sheets to only ∼15% and ∼24%, respectively (Figure 4B), concomitantly increasing unordered and aggregated structures from nearly undetected levels in untreated MS2 ^8^ to ∼24% and ∼13%, respectively. Similar trends were observed in the Fe–Fe system and at the higher pH, but to lesser extents (Figure 4B). The $\frac{\alpha ‐helix}{\beta ‐sheet}$ ratio (olive green color in Figure 4B) remained unchanged by FeCl_3_ coagulation but was markedly reduced by electrocoagulation^8^ in the sequence Fe–Fe at pH 6.5 > Fe–C at pH 6.5 > Fe–Fe at pH 5.5 > Fe–C at pH 5.5, indicating progressively more intense structural alterations and increasing treatment severity in this order. Relative differences in the secondary structures followed the same trend as the extent of nonproteolytic modifications detected by MALDI-TOF-MS (Figure 2) and LRVs (Figure 1B,D), offering insights into specific amino acid backbone modifications likely mediating conformational destabilization.^57,58^

Oxidative damages arising from electrocoagulation treatment were evaluated by monitoring the appearance of new peaks in the carbonyl^8,82^ region (Figure 4C and Supporting Information Tables S12,S13II). Untreated phages did not exhibit any peaks in the 1820–1700 cm^–1^ range, which remained substantially unchanged^82^ following conventional coagulation at both pH values. Note that carbonyl groups in untreated viruses are conjugated within peptide bonds (–CO–NH–) and appeared only in the amide I region (1700–1600 cm^–1^),^43,56^ with no unconjugated C=O detectable in the oxidative marker region (1820–1700 cm^–1^). New peaks emerged at 1704, 1716, 1732, and 1749 cm^–1^ after Fe–C electrocoagulation at pH 6.5 (Supporting Information Figure S16) corresponding to aliphatic aldehydes (∼0.9%), anhydrides (0.66%), and aliphatic ketones (∼1.4%). Lowering the pH to 5.5 intensified these peaks and led to the appearance of additional ones at 1771 and 1791 cm^–1^ (Figure 4C) corresponding to aliphatic aldehydes (2.1%), anhydrides (0.7%), and acyl chloride (∼0.4%) while lowering the contribution from aliphatic ketones (∼1%), symptomatic of a redistribution of carbonyl species under more acidic conditions. As expected, Fe–Fe electrocoagulation gave similar trends (see Supporting Information Section S12). Introduction of specific carbonyl functionalities was anticipated from modeling and MALDI-TOF-MS results. For example, glutamic semialdehyde can be catalytically formed via oxidation of the Arg49 residue^56,79^ nearest to the Ser23-Cys46 docked region (Supporting Information Table S8) and was consistent with the –43 Da mass-shift peak (Figure 2). Additionally, oxidation of Thr45 at its β-carbon has been shown to form aliphatic ketones.^83,84^ Further, the carboxyl side chain of Glu31, located within the ferryl-interacting region (Supporting Information Figure S13), can undergo side-chain condensation to form anhydrides^43,58^ or participate in substitution reactions (with chloride ions in solution) forming acyl chloride.^43,58^ Evidence for oxidation was also obtained via S–O peaks even though they suffer from severe overlap with water specifically ν_3_(SO_4_^2–^)^85^ and the virus RNA ν­(PO_2_^–^).^8^ Electrocoagulated viruses exhibited new ν­(S–O_*x*_) peaks at 1191, 1138, 1136, 1135, 1049, and 1048 cm^–1^ (Supporting Information Figure S16), indicating thiol oxidation in cysteine^15,53,70^ (attributed to Cys46 located within the ferryl-interaction region; Figure 3A and Supporting Information S13).

The $\frac{\alpha ‐helix}{\beta ‐sheet}$ ratio was strongly and inversely correlated to log-inactivation (Figure 4D), total carbonyl content (Figure 4E), and MALDI-TOF-derived protein damages (Figure 4F). Therefore, progressively greater disruption of secondary structures (i.e., lower $\frac{\alpha ‐helix}{\beta ‐sheet}$ values than the untreated virus) quantitatively corresponded to increased extent of oxidation and functional loss of the coat protein^82^ (also see Supporting Information Figure S18). Consistent relationships across independent analytical techniques, viz., FTIR and MALDI-TOF-MS underscored the potential structural basis of the measured inactivation during electrocoagulation and the lack of inactivation by conventional iron coagulation.

### 3.3 Electrochemically Generated Intermediates and Their Relation to Inactivation

Thermodynamic modeling of saturation states and mineral solubility revealed strongly pH-dependent reactions that controlled Fenton chemistry (Supporting Information Section S9, Figure S6, and Table S4). Fe­(II) was (i) generated at 100% Faradaic efficiency (Supporting Information Section S2 and Figure S1),^7,8^ (ii) strongly pH dependent in terms of its temporal profiles^8,50,51^ but less so on the cathode material (Figure 5A), and (iii) present in substantial amounts throughout all experiments (Figure 5a and Supporting Information Figure S19). As thermodynamically predicted and already reported,^50–52^ more Fe­(II) was present at the lower pH explained by the strong inverse pH dependence on its overall oxidation rate constant.^50,51^ The remaining iron was always converted stoichiometrically to the total iron Faradaic target and predicted to be Fe­(III)-(oxyhydr)­oxide polymorphs^50,52^ (Supporting Information Table S4 and Figure S6). Importantly, Fe­(II) concentrations were always slightly lower in the Fe–C system at both pH values, which is consistent with H_2_O_2_ profiles, as explained next.

**Figure 5:**
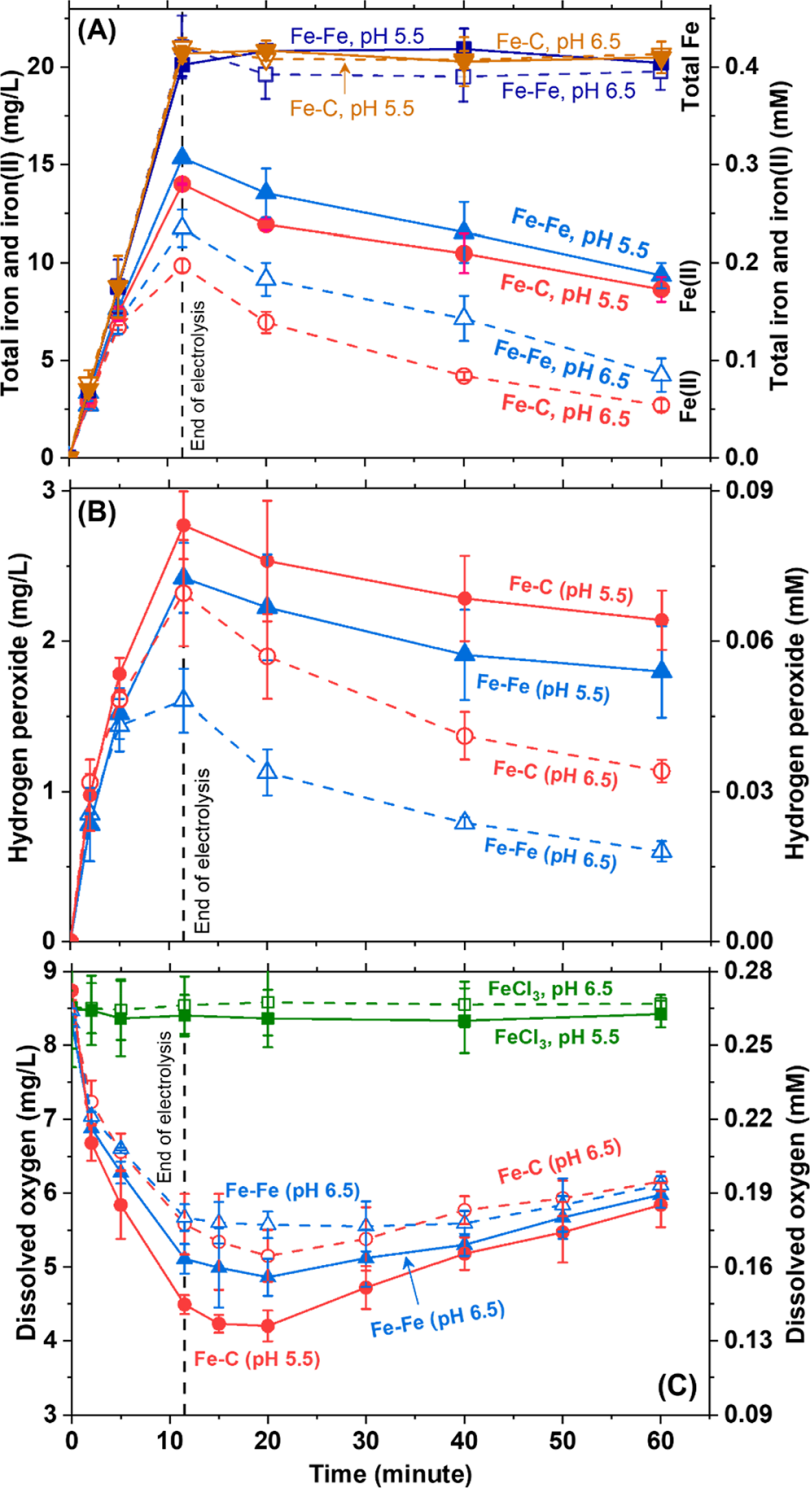
Transient profiles of (A) Fe­(II) and total Fe, (B) H_2_O_2_ concentrations, and (C) dissolved oxygen concentrations for Fe–C and Fe–Fe electrocoagulation at pH 6.5 and 5.5. Iron and H_2_O_2_ concentrations corresponding to total iron doses of 5 and 10 mg/L are shown in Supporting Information Figures S19 and S21. Open and closed symbols correspond to experiments at pH 6.5 and 5.5, respectively. Data represent average ± standard deviation from three independent electrocoagulation experiments.

Unlike Fe­(II), temporal profiles of the other Fenton precursor, viz., H_2_O_2_ strongly depended on both the cathode material and pH (Figure 5B): significantly greater amounts were generated by replacing the carbon steel cathode with graphite and reducing the pH from 6.5 to 5.5 attributed to weaker adsorption of the *OOH intermediate (formed via electrochemical reduction of dissolved oxygen, Figure 5C and Supporting Information Section S3).^86^ Correspondingly, apparent H_2_O_2_ Faradaic efficiencies were higher with the graphite cathode and at pH 5.5 (Supporting Information Section S13b; Figures S20 and S21, and Table S14). These efficiencies are conservative estimates because of rapid *in situ* peroxide consumption by Fe­(II) and decay during measurement.^8,13,86^ Note that the anode was kept unchanged and always produced Fe­(II) at 100% Faradaic efficiency independent of experimental conditions (Supporting Information Figure S1). Hence, trends in H_2_O_2_ measurements in Figure 5B corresponded to the initiation and progression of Fenton reactions, i.e., Fe–C at pH 5.5 > Fe–Fe at pH 5.5 > Fe–C at pH 6.5 > Fe–Fe at pH 6.5. Crucially, this sequence was identical to (i) virus inactivation (Figure 1), (ii) modifications to protein secondary structures and the $\frac{\alpha ‐helix}{\beta ‐sheet}$ ratio (Figure 4B–F), and (iii) nonproteolytic damages in MALDI-TOF and carbonyl formation (Figures 2 and 4C). The trend in the extent of Fenton reactions was validated by the slightly lower Fe­(II) concentrations (or more Fe­(II) consumption) in Figure 5A for the Fe–C system at both pH values. Further, control experiments (Supporting Information Section S14, Figures S22–S23) showed that H_2_O_2_ alone negligibly inactivated MS2 (LRV < 0.3)^8,13^ and Fe­(II) alone yielded modest LRVs (<1.7), underscoring their enhanced virucidal efficacy in combination via electro-Fenton reactions during iron electrocoagulation.^8^

## 4 Implications

Electrocoagulation with a low-carbon steel anode and a graphite cathode (i.e., Fe–C electrocoagulation) at slightly acidic conditions reduced MS2 to below detection limits (LRVs ≳ 6.7), substantially exceeding Surface Water Treatment Rule requirements (LRV = 4) in ≤11.5 min, well within the design flocculation residence time of 20 min for conventional treatment. Although lower LRVs were measured using low-carbon steel anode and cathode (i.e., Fe–Fe electrocoagulation), they were still greater than what was obtained via conventional coagulation due to concurrent removal and inactivation.^7,8^ Hence, iron electrocoagulation appears to be well-suited to provide virus credits particularly during (in)­direct potable reuse where substantially greater LRVs are required compared to surface water treatment.^3–5,21–23,37^ Additionally, inactivation was increased (by ≳1.5-logs at 60 min) when the pH was lowered from 6.5 to 5.5, analogous to improved disinfection byproduct (DBP) precursor removal by “enhanced coagulation” as already being practiced in many treatment plants, e.g., ref 6. Hence, we recommend adopting a similar approach (i.e., “enhanced electrocoagulation”) and broaden this technology’s treatment objectives, i.e., improved control of viruses, DBP precursors, and turbidity. Whereas previous electrocoagulation investigations have largely focused on the role of the sacrificial anode, e.g., refs 7, 8, and 10, measurements reported herein add to the smaller subset of publications targeting the crucial role played by dimensionally stable cathodes in enhancing the control of chemical contaminants^10^ and now viruses. Substantial LRV improvements during the ∼50 min of flocculation (e.g., by ∼2 at pH 5.5 in the Fe–Fe system) indicated the benefits of extended slow-mixing durations beyond the 20 min design value. These process engineering advantages coupled to its portability, lower greenhouse gas emissions, and reduced need for permanent infrastructure to store corrosive chemicals^9,10^ further reinforce the promise of iron electrocoagulation that however need to be critically evaluated via longer-term testing at larger scales. Although a detailed technoeconomic analysis is beyond the scope of this work, other bench- and pilot-scale investigations have shown iron electrocoagulation to be technically and economically feasible for small and decentralized systems.^87–90^ It is emphasized that results herein were generated in the absence of organic matter, as has been often practiced in well-controlled mechanistic laboratory-scale virus studies,^13,14,17,19,25^ again pointing to the need for validation using larger-scale experiments with real secondary wastewater effluents.

This is an important issue because organic matter can influence ROS formation and concentrations during electrochemical disinfection and consequently the kinetics and magnitude of virus inactivation.^7,91,92^ Natural and effluent organic matter can (i) act as ROS sinks,^93,94^ transforming ROS into less reactive organic-centered radicals,^95^ (ii) suppress electroFenton reactions by complexing Fe­(II),^95,96^ and (iii) potentially foul electrodes to reduce Faradaic efficiency of Fe­(II) and H_2_O_2_ electrodissolution.^35,97,98^ Organics can also associate with viruses, change their surface properties, and reduce oxidant access to their capsids/genome.^99,100^ These phenomena can be expected to reduce virus LRVs during electrocoagulation of real (waste)­waters containing organic matter, although its magnitude is difficult to predict. Regardless of the underlying mechanisms, organic matter has been empirically shown to lower virus LRVs in treatment systems.^91,101^ Hence, virus LRVs during electrocoagulation of actual (waste)­waters laden with organic matter can be expected to be lower than those reported herein (additional details are in Supporting Information Section S15). In any case, incorporating any type of coagulation to augment virus LRVs will also improve downstream process performance (e.g., membranes or ozone)^3,5^ and conform to California’s requirement to use “diverse treatment mechanisms” during potable reuse.

Mass spectrometry, infrared spectroscopy, and DFT modeling demonstrated that specific MS2 capsid protein residues were attacked by ferryl ion similar to ^1^O_2_,^17^ ferrate,^19^ and UV_254_ ^17^ but unlike free chlorine^14^ and ^•^OH^66^ that tend to act indiscriminately. Interestingly, the same residue identified in this work (Cys46) has also been implicated for damage by UV_254_ absorption^17^ and is part of an RNA-binding motif,^24^ essential for stable genome–protein interactions and capsid integrity. This ferryl oxidation hotspot (Cys46) may offer an alternative nonproteolytic inactivation pathway for MS2 inactivation in light of Cys101 being identified as being sensitive to ^1^O_2_.^17^ Although we connected local oxidative chemistry to virus inactivation, it is unclear whether such modifications are necessary and sufficient conditions for infectivity loss, as has been previously cautioned for the case of disinfection.^17^ Nevertheless, site-specific reactions accompanying inactivation identified herein might explain the widely different LRVs achieved for dissimilar viruses (e.g., ϕ6, P22, ϕx174)^7,8^ by iron electrocoagulation as well as for the same virus by different treatments (an incomplete list for MS2 is in Supporting Information Table S15) and suggests new research avenues to mechanistically determine inactivation pathways (i.e., individual amino acid sites). This study investigated MS2 as a surrogate for nonenveloped human pathogens as commonly practiced in (waste)­water treatment investigations.^7,12^ However, viruses exhibit a high degree of diversity in terms of their morphology (e.g., presence of an envelope or tail), capsid composition, and genome type, each of which can influence individual oxidative inactivation pathways and LRVs. Hence, results from this article strictly apply only to MS2 and need to be cautiously extrapolated to other viruses. For these reasons, we are currently pursuing experiments to validate and extend ROS-driven oxidation mechanisms and identify amino acid targets for inactivation of dissimilar bacteriophages representing various virus realms, with the ultimate goal of determining inactivation mechanisms of human pathogens.

## Supplementary Material



## Notes

Extensive details on the electrocoagulation system, water composition, MALDI-TOF-MS and ATR-FTIR analyses, equilibrium speciation calculations, spatial interaction mapping, and DFT cluster modeling including computational setup, functionals, basis sets, solvation models, optimization trajectories, spin-state surveys, additional checks, and all associated methodological assumptions and limitations (PDF)
