# Supplementary material for: Virus Capsid Modifications Accompanying Inactivation during Iron Electrocoagulation Revealed by Proteomics, Infrared Spectroscopy, and Molecular Modeling
Source: Environ Sci Technol. 2025 Dec 31;60(1):1488–502. doi: 10.1021/acs.est.5c13277 (PMC12810256; doi:10.1021/acs.est.5c13277)
Supplement: Supplementary file 1 [file es5c13277_si_001.pdf]

SUPPORTING INFORMATION

Virus capsid modifications accompanying inactivation during iron electrocoagulation revealed by proteomics, infrared spectroscopy, and molecular modeling

Akshat Verma[1], Shankararaman Chellam[1,2,*]

[1]Department of Civil & Environmental Engineering, Texas A&M University, College Station, TX 77843-3136

[2]Department of Chemical Engineering, Texas A&M University, College Station, TX 77843-3122

Corresponding author: S. Chellam, Zachry Department of Civil & Environmental Engineering, Texas A&M University, College Station, TX 77843. Phone: (979) 458 5914; chellam@tamu.edu

Number of Sections: 15

Number of Figures: 23

Number of Tables: 15

Methods

Section S1. Details related to phage and synthetic/model water

S1a. Phage selection, purification, and propagation

Bacteriophage MS2 was used as a surrogate of enteric viruses to evaluate electrocoagulation performance.[1-3] It was selected based on its structural similarities towards several human pathogenic ssRNA viruses including those currently listed on Contaminant Candidate List 5 (CCL5)[1] by the United States Environmental Protection Agency (USEPA). MS2 was propagated and purified following previously reported protocols [4, 5] with slight modifications. For MS2 purification, host *Escherichia coli* (ATCC 15597) was grown up to a mid-log phase (i.e., OD_{600}: 0.3-0.5) in 100 mL tryptic soy broth (TSB; tryptone 17 g/L, soytone 3 g/L, dextrose 2.5 g/L, NaCl 5 g/L, K_2HPO_4 2.5 g/L, pH 7.5) at 37 °C. Afterwards, phages were added to the bacterial suspension at multiplicity of infection (MOI) of 0.1 with supplement of 1 mM $MgSO_4$. After an extra period of 16 hours of incubation at 37 °C, bacterial cell debris was centrifuged at 13,500 g for 20 min at 4°C and the supernatant was filtered with 0.2 and 0.1 μm polyethersulfone (PES) membrane filter. Phages remaining in the filtrate were ultracentrifuged at 104,000 g for 12 hours at 4 °C to form a pellet, which was resuspended overnight in 1 mL of phosphate buffer saline (PBS; 4.8 mM of NaH_2PO_4, 17.6 mM of Na_2HPO_4, 145.4 mM of NaCl, pH 7.5). The titer of infective MS2 in resulting stock was ~10^{12} PFU/mL measured by the double-agar layer method.[6]

S1b. Model water composition

Based on one case of direct potable reuse (Case 1) and another of indirect potable reuse (Case 2),[7-11] we formulated the composition (Table S1) to mimic real-world municipal wastewater secondary effluent. Although we closely captured the inorganic composition, we refrained from adding organic matter to avoid mass spectral interferences and consumption of reactive oxygen species.

Table S1. **Water composition of two case studies and formulated model secondary effluent water used in this study.**

Parameter	Case 1 (direct potable use)		Case 2 (indirect potable use)		Synthetic secondary effluent (this study)	
**I. Cations**	**mg/L**	**mM**	**mg/L**	**mM**	**mg/L**	**mM**
Ca^{2+}	78.3	2.0	79.7	2.0	75	1.9
Mg^{2+}	18.8	0.8	28.6	1.2	25	1.0
Na^{+}	368	16.0	225	9.8	225	9.8
**II. Anions**	**mg/L**	**mM**	**mg/L**	**mM**	**mg/L**	**mM**
SiO_3^{2-}	45.0	0.6	20.3	0.3	30	0.4
Cl^{-}	289	8.1	285	7.9	300	8.5
SO_4^{2-}	268	2.8	195	2.0	200	2.1
HCO_3^{-}	134	2.2	246	4.0	122	2.0
Hardness (as $CaCO_3$)	253	2.5	317	3.2	292	2.9
Alkalinity (as $CaCO_3$)	110	1.1	202	2.0	~100$^{\alpha}$	~1$^{\alpha}$
**General parameters**	**Units mentioned in parentheses**					
pH (no unit)	7.0		7.2		5.5 and 6.5$^{\beta}$	
Conductivity (µS/cm)	n.a.		1680		1550	

$^{\alpha}$ concentrations depend on the pH values

$^{\beta}$ pH was adjusted using HCl

n.a: not available

Section S2. Faradaic efficiency of iron in electrocoagulation systems

For Fe-Fe and Fe-C systems, Faradaic efficiencies were calculated using equation S1.

$$Faradaic\ efficiency\ (\%) = \frac{Measured\ concentration}{Theoretical\ dose} \times 100 \qquad (S1)$$

where, theoretical iron dose was estimated using equation S2 and measured concentrations of total iron was determined per details mentioned in SI Section S5b

$$Theoretical\ dose\ \left(\frac{mg}{L}\right) = \frac{1000A_wIt}{ZFV} \qquad (S2)$$

where, A_w represents molecular mass of Fe 55.85 g/mol, I represents current (0.05 A), t is time (~ 691 s for 20 mg/L of Fe dosage, 346 s for 10 mg/L of Fe dosage, and 173 s for 5 mg/L of Fe dosage), F is Faraday constant (~96,485 C/mol), Z represents number of electrons transferred (2 for Fe), V is reactor volume (0.5 L). Faradaic efficiencies for total Fe for both Fe-Fe and Fe-C electrocoagulation systems at different pH are represented in SI Figures S1.

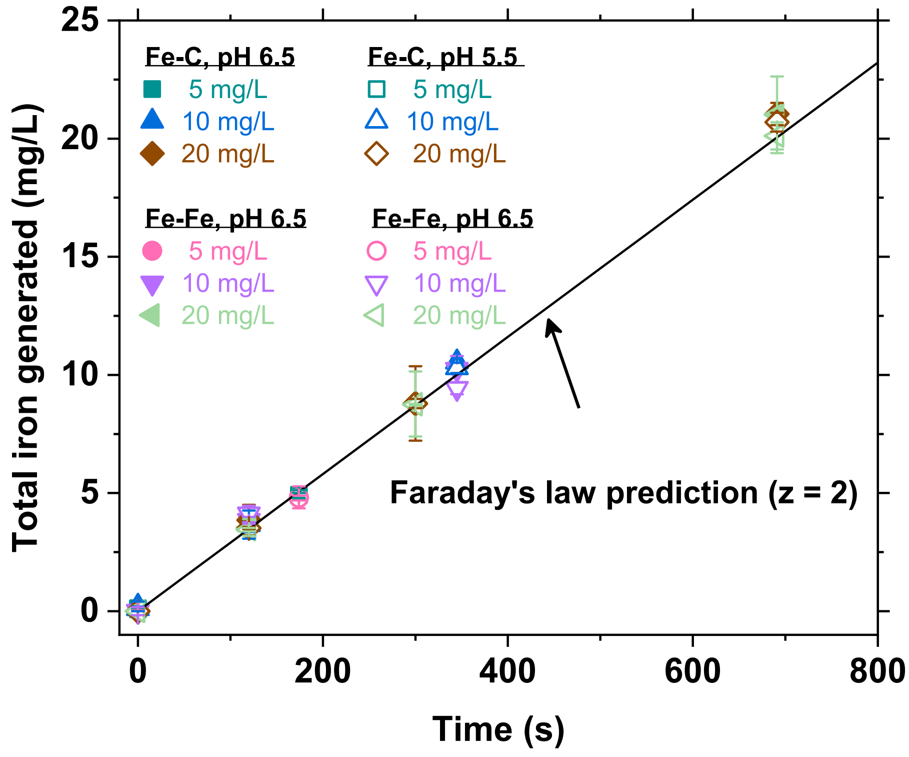

Figure S1. Iron dosing during Fe-Fe and Fe-C electrocoagulation showing excellent agreement between experimental data and Faraday's law with 2-electron transfer.at pH 6.5 and 5.5.

Table S2. **Chemical composition of electrodes used in this study.**

Iron electrode	
**Element**	**% (by weight)**
Fe	>98.9
C	<0.25
Cu	>0.20
P	<0.03
Si	<0.40
S	<0.03
**Carbon electrode**	
C	100
Impurities	none

[a] Link for Fe (carbon steel) electrode: https://www.mcmaster.com/1388K454/
[b] Link for C (graphite) electrode: https://www.mcmaster.com/9121K61/

S3. Reason for higher production of H_2O_2 with carbon (graphitic) electrode [12-14]

Electrochemical reduction of O_2 takes place in two ways: (a) Four electron reduction of O_2 to generate H_2O, and (b) two electron reduction of O_2 to generate H_2O_2. These reactions are detailed in equations S3 and S4.

$$O_2 + 4e^- + 4H^+ \rightarrow 2H_2O \qquad E_H^0 \sim 1.23\ V \qquad \text{S3}$$

$$O_2 + 2e^- + 4H^+ \rightarrow 2H_2O_2 \qquad E_H^0 \sim 0.7\ V \qquad \text{S4}$$

Intermediate reactions for $4e^-$and $2e^-$ reductions are mentioned in equations S5 and S6

$$O_2 + 4e^- + 4H^+ \rightarrow *OOH + 3(H^+ + e^-) \rightarrow *O + 2(H^+ + e^-) \rightarrow *OH + 1(H^+ + e^-) \rightarrow 2H_2O \qquad \text{S5}$$

$$O_2 + 2e^- + 2H^+ \rightarrow *OOH + (H^+ + e^-) \rightarrow H_2O_2 \qquad \text{S6}$$

$*OOH$ is a common intermediate formed during 4 and 2 electron reduction of O_2. For the generation of H_2O_2, dissociation of O-O bond from $*OOH$ is necessary. Depending on the material property of the electrocatalyst, *OOH has strong or weak adsorption on the surface. Strong adsorption of *OOH favors the 4e$^-$ reduction over 2e$^-$ reduction due to lack for O-O bond dissociation. Therefore, weak adsorption of *OOH favors the 2e$^-$ reduction of O_2 to generate H_2O_2. Carbonaceous electrocatalysts only weak adsorb *OOH, which favors the formation of H_2O_2.

Section S4. Relevant details related to electrocoagulation experiments

S4a. Operational details

The batch electrocoagulation system was evaluated for electrode stability, electric potential development during short-term operation, iron dosing control, and temporal variations in aqueous parameters in synthetic secondary effluent under different pH conditions. HCl was used to adjust the pH. The pH of the electrolyte was 6.5 and 5.5. The lower pH was selected to promote virus inactivation by enhancing the generation of Fenton's reagents (Fe(II) and H_2O_2), thereby facilitating the formation of reactive oxygen species (ROS). All experiments were triplicated, and averages and standard deviations are reported. Wetted area of electrodes was ~50 cm^2 (5 cm × 5 cm for one side), SI Table S2] for both Fe-Fe and Fe-C systems. After each electrocoagulation run, the electrodes were cleaned with HCl solution (pH~3) and rinsed with deionized water to remove residual iron. Mechanical cleaning was performed sequentially with 120- and 220-grit sandpapers, followed by thorough rinsing with deionized water. Carbon cathode has been previously used for removal of various pollutants,[15, 16] but not viruses.

Only a single bacteriophage, viz. MS2 was used in this study as a model of enteric viruses[17, 18] to examine inactivation mechanism(s) in much detail during electrocoagulation. MS2 is a very well characterized RNA phage and widely employed in water and wastewater treatment investigations.[4, 19-26] Additionally, a single starting virus concentration (order of 10^7 pfu/mL) and the same initial MS2 inoculum was used across all conditions to ensure comparability between different experimental conditions used in the study. This consistent initial concentration allowed clear interpretation of electrochemical and oxidative effects across the three iron dosages (5, 10, and 20 mg Fe/L), two pH values (6.5 and 5.5), and two electrocoagulation systems (Fe-Fe and Fe-C).

S4b. Relevant reactions involved in iron electrooxidation experiments[20, 27-29]

**Details**	**Reaction**	
Oxidation of Fe(0) at anode	$Fe(0) \rightarrow Fe(II) + 2e^-$	S7
Oxidation of Fe(II) by $O_{2(aq)}$	$Fe(II) + O_{2(aq)} \rightarrow Fe(III) + \cdot O_2^-$	S8
Oxidation of Fe(II) by $\cdot O_2^-$	$Fe(II) + \cdot O_2^- + 2H^+ \rightarrow Fe(III) + H_2O_2$	S9
Reduction of Fe(III) by $\cdot O_2^-$	$Fe(III) + \cdot O_2^- \rightarrow Fe(II) + O_2$	S10
Relevant Fenton's reaction	$Fe(II) + H_2O_2 \rightarrow Fe(III) + \cdot OH + OH^-$	S11
	$Fe(II) + H_2O_2 \rightarrow Fe^{IV}O^{2+} + H_2O$	S12
Production of $H_{2(g)}$ at cathode	$2H^+ + 2e^- \rightarrow H_{2(g)}$	S13
Production of H_2O_2 at cathode	$2H^+ + O_2 + 2e^- \rightarrow H_2O_2$	S14
Formation of iron flocs	$Fe(III) + 3H_2O \rightarrow Fe(OH)_{3(s,am)} + 3H^+$	S15
Utilization of Fenton's reaction products	$\cdot OH + Fe(II) \rightarrow OH^- + Fe(III)$	S16
	$Fe^{IV}O^{2+} + Fe(II) + 2H^+ \rightarrow H_2O + 2Fe(III)$	S17

Under electrocoagulation conditions, conventional probe- or quencher-based ROS assays are difficult to apply because various factors interfere with probe stability and signal interpretation. This includes the continuously varying Fe(II)/Fe(III) redox environment, evolving Fe(oxy)hydroxide flocs, and gas generation near electrodes. As a result, standard fluorescence or scavenger assays cannot reliably distinguish oxidant species or quantify rates under these heterogeneous, current-driven conditions.

Instead, our mechanistic interpretation of ROS in this manuscript draws on extensive literature that has directly identified ferryl intermediates in Fe-based systems operating under similar pH and redox conditions. Previous studies[30, 31] have established that in homogeneous Fe(II)/H_2O_2 (Fenton) systems, oxidant speciation shifts from hydroxyl radical–dominated chemistry at acidic pH (< 4) to ferryl ion intermediates at mildly acidic to near-neutral pH (~5–7). More recent work[32] has demonstrated that this same ferryl-favoring transition also occurs in electrochemical Fenton-like systems, where anodic Fe dissolution and cathodic H_2O_2 generation create redox conditions comparable to those in electrocoagulation. Further confirmation of ferryl formation through ^{18}O labeling[33] and complementary probe and quencher validations[34, 35] have provided additional mechanistic support consistent with the oxidation patterns observed herein.

Section S5. Virus quantification and other analytical methods

S5a. Details related to aqueous phase analysis

Solution pH, dissolved oxygen, and conductivity in the bulk solution were monitored by a pH probe (Orion Basic, Thermo Fisher Scientific), a luminescent/optical meter (LDO101, HACH), and a conductivity probe (CDC401, HACH), respectively.[4, 36] Total iron was quantified per the HACH 8112 method where ferric iron, if any, is reduced by sodium metabisulfite to ferrous iron forming a purple-colored complex with 2,4,6-tripyridyl-s-triazine (TPTZ) at a ratio of 1:2. The complex was quantified at a wavelength of 590 nm (HACH DR6000).[37, 38] The HACH 8146 method was used to quantify ferrous iron concentrations after chelating it with 1,10-phenanthroline to form a pink complex which was quantified by light absorbance at 510 nm (HACH DR6000).[37] H_2O_2 was measured using previously described Cu(I)-2,9-dimethyl-1,10-phenanthroline method based on the formation of an orange-colored complex that was quantified at 454 nm[39, 40] (HACH DR6000). Reduction of Cu(II) by Fe(II) in the filtered (0.2 µm) samples was prevented by the addition of 7.2 mM sodium citrate.[36]

S5b. Virus quantification using double layer method

All electrocoagulation experiments were conducted in triplicate and plaque assays were performed using at least four plates per sample, each containing a minimum of 10 plaques to ensure statistical reliability. The phage detection limit was 10 PFU/mL for the bulk solution and 100 PFU/mL for the total suspension (bulk + flocs) based on assay volumes of 1 mL and 0.1 mL, respectively. 6% beef extract was freshly prepared before experiments and adjusted to pH ~ 9.5. Plaque counts from replicate plates were averaged, and the variability was expressed as standard deviations. All glassware and filtration assemblies were acid-washed and autoclaved before use to minimize background contamination.

Section S6. MS2 coat protein analysis using MALDI-TOF-MS

S6a. Instrument resolution and control validation

The Matrix-Assisted Laser Desorption/Ionization Time-of-Flight Mass Spectrometer (MALDI-TOF-MS) was operated and controlled using Bruker Daltonics FlexControl, and data were analyzed using Bruker Daltonics FlexAnalysis. For this study, 91% of the laser power was used for the analysis. The instrument was operated with ion sources of 19.99 kV (ion source 1) and 18.99 kV (ion source 2).

For this study, an m/z range of 3,000 to 15,500 was scanned. Analysis was performed in two m/z windows (3000–6200 and 6200–15500) to optimize detector sensitivity and resolution across the full mass range, ensuring accurate detection of both low- and high-mass protein/fragment peaks. Prior to any analysis in MALDI-TOF, we first confirmed that synthetic secondary water used in this study (Table S1) was free from any protein contamination (Figure S2). No potential peaks were observed at pH 6.5 and 5.5 between m/z range 6,000 to 16,000.

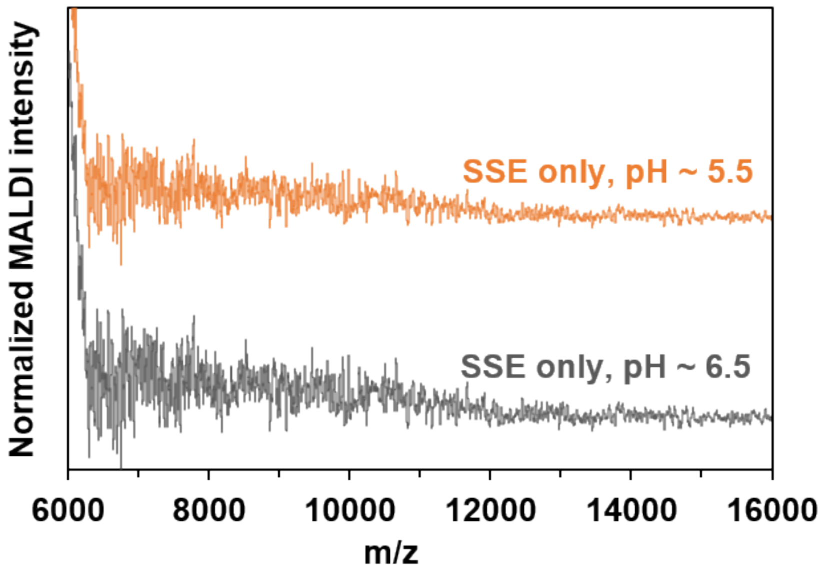

Figure S2. MALDI-TOF analysis of synthetic secondary effluent (SSE) at pH 6.5 and 5.5. The scan was performed between m/z range of 6,000 to 16,000 encompassing the region of interest for the coat protein of untreated MS2.

Next, we calibrated the instrument based on our experimental conditions. For this we used 1 mg/mL cytochrome in the same background as the viral samples (synthetic water, Table S1) to assess instrumental resolution under conditions matching our experimental setup in linear mode. The $[M+H]^+$ peak of cytochrome c appeared at m/z 12,360 with a full width at half maximum (FWHM) of 11.36 Da, corresponding to a resolution of approximately 1,086 (estimated as resolution = m/Δm, where m = 12,360 and Δm is mass shift or FWHM) (Figure S8a,b). Based on this experimentally validated FWHM, mass shifts within ±11 Da may not be confidently distinguished, whereas shifts beyond this value can be reliably interpreted (such as +16, +32, +48, and –43 Da observed in virus samples as discussed in Section 3.2).

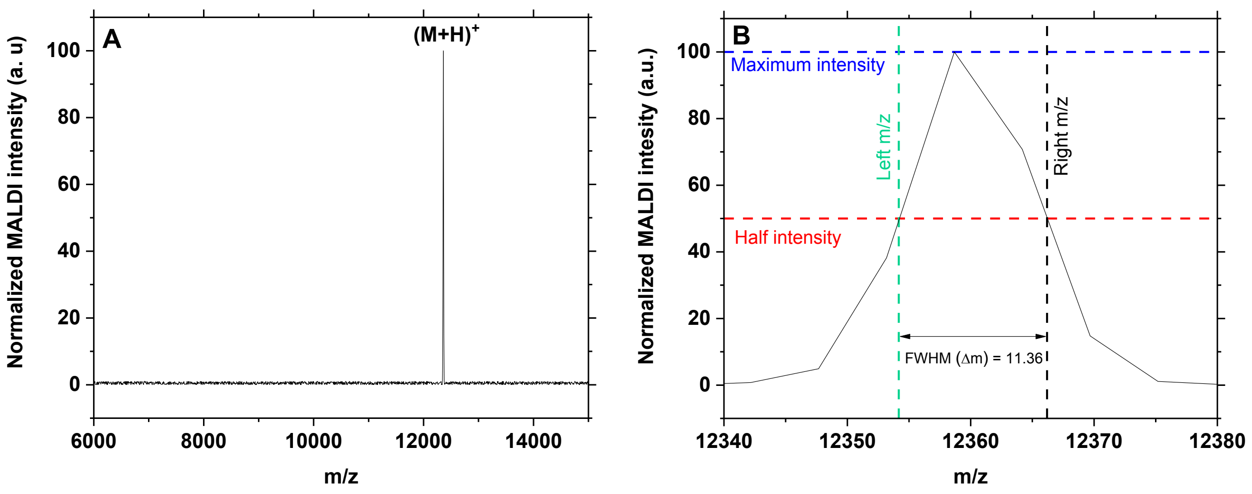

Figure S3. (A) MALDI-TOF-MS spectrum of cytochrome c $[M+H]^+$ acquired in linear mode under synthetic water conditions used in this study (Table S1), showing a prominent peak at m/z 12,360. **(B)** Full width at half maximum (FWHM) analysis of the cytochrome c peak, yielding a value of 11.36 Da, which establishes the minimum resolvable mass difference under these experimental conditions.

S6b. Sample preparation

For this analysis, conventionally coagulated and electrocoagulated samples were prepared in a similar way, but in a smaller working volume (100 mL). The initial virus concentration was ~10^9 PFU/mL. After the end of the experiments (60 minutes), 50 mL of coagulated samples were immediately quenched for further virus attenuation by adding 25 mM sodium sulfite. The

suspension was then sonicated for 5 minutes to detach virus from the flocs. The suspension was then stored at 4 °C until further use.

For the analysis, 15 μL of sample (untreated or (electro)coagulated MS2) sample was diluted with 1% trifluoracetic acid (TFA). The sample was desalted and concentrated using the C4 column (ZipTip® Pipette Tips P10, Millipore). Subsequently, 2 μL of matrix was added to the column to elute protein from the column. Matrix consisted of 10 mg of α-cyano-4-hydroxycinnamic acid dissolved in 1 mL of solvent (1:1 acetonitrile and 0.1% TFA). Subsequently, a protein-deposited matrix was loaded in MALDI plate (MTP 384 target polished steel BC, Bruker). The sample was dried at 40 °C (Type 37900, culture incubator). Subsequently, the plate was then loaded in the instrument (Ultraflextreme, Bruker) for the analysis.

S6c. Reproducibility and deconvolution of MALDI-TOF spectra

Three replicate spectra were recorded to confirm reproducibility. Average of these duplicate spectra is presented in Figure 2 of the main manuscript. The average intensity profiles were closely aligned with their corresponding replicates, with correlation coefficients ranging from 0.95 to 0.99. This high level of reproducibility supports the reliability of the MALDI-TOF measurements and validates the observed (oxidative) modifications under different electrochemical and coagulation conditions. Section S10 and Figure S10 details a comprehensive investigation of spectral reproducibility.

MALDI-TOF-MS spectra[41, 42] were deconvoluted by integrating peak areas within ±5.5 Da of each expected mass-to-charge (m/z) value, corresponding to the half-width of the experimentally validated FWHM (11.36 Da, Figure S3). Specifically, integration windows were set as follows: $[M+H]^+$ (13,724.5–13,735.5), $[M+H+16]^+$ (13,740.5–13,751.5), $[M+H+32]^+$ (13,756.5–13,767.5), $[M+H+48]^+$ (13,772.5–13,783.5), and $[M+H-43]^+$ (13,686.5–13,697.5) (Section S10

along with Table S6 and Figure S12 details comprehensive peak deconvolution). All peaks falling within each defined window were considered indistinguishable due to instrument resolution and were assigned to the corresponding oxidation or cleavage product. The relative area of each window was then used to quantify the distribution of oxidative modifications in the viral coat protein (Figure 2). Peaks were better resolved considering local maxima method.

Section S7. Spatial interaction mapping and cluster-based modeling

S7a. Reason for two-phase modeling approach

We used a two-phase workflow: (i) spatial interaction mapping (non-reactive docking) to identify sterically feasible approaches of reactive intermediates to the MS2 coat protein, followed by (ii) a local quantum mechanical (QM) cluster to examine coordination and electronic polarization at the candidate site. Docking was used strictly only to nominate contact-prone residues (≤ 6.0 Å); all chemical interpretation derives from the QM analysis. Docking scores (kcal/mol) is software heuristics used only for pose ranking, not for binding thermodynamics.

Spatial interaction mapping or molecular docking can be conventionally used to model stable, noncovalent ligand–protein interactions.[43, 44] In this study, it was employed as an initial screening tool to identify surface-exposed residues that may be prone to oxidative attack by highly reactive, short-lived species such as hydroxyl radicals ($^{\bullet}OH$) and ferryl ion ($[Fe^{IV}O]^{2+}$ or simply represented as Fe(IV)=O). Given the non-classical and transient nature of these ROS,[45, 46] and the fact that standard docking scoring functions are not parameterized for high-valent metal–oxo species or radicals docking outputs were not interpreted as binding free energies or reactivity predictions, but rather only as indicators of spatial accessibility and proximity to potential reactive sites on the MS2 coat protein.[47-49] Accordingly, scores/estimated K_i are treated as heuristic pose-ranking metrics only. Similar strategies have been reported previously, such as docking of radical-

scavenging ligands like flavin and hydroxyurea to tyrosyl radical sites in human ribonucleotide reductase to identify oxidation-prone regions.[47-49]

To complement this spatial screening, a cluster-based quantum mechanical (QM) model was used to further evaluate the feasibility of coordination and electronic polarization consistent with oxidative engagement at the most promising residue–ligand interface identified from docking. This two-phase approach allowed both geometric and electronic factors to be considered when assessing susceptibility to reactive oxygen species (ROS)-mediated capsid damage (Figure S4). Results from these modeling efforts were subsequently compared with experimental evidence of oxidative modification observed in MALDI-TOF mass spectrometry (comparative analyses reported in the main text).

While more detailed mechanistic simulations—such as reactive molecular dynamics or solvent accessibility analyses—may provide additional insights, this combined docking and QM strategy offers a practical and chemically grounded framework for prioritizing oxidation-prone contact sites on viral coat proteins exposed to electrochemically generated ROS.

S7b. Software required

Flowchart of molecular docking process is shown in Figure S4**.** The following software packages were downloaded and installed on Windows 11.

a) MGLTools1.5.7: Used for ligand and receptor preparation, adding charges, and converting file formats for AutoDock (https://ccsb.scripps.edu/mgltools/downloads/)[50, 51]
b) AutoDock4.2.6: Performed molecular docking using the Lamarckian Genetic Algorithm (https://autodock.scripps.edu/download-autodock4/)[50, 51]
c) UCSF ChimeraX v1.9: Used for 3D structure visualization, receptor editing, residue capping, and measuring distances (https://www.cgl.ucsf.edu/chimerax/download.html)[52, 53]

d) Discovery Studio: Used to visualize molecular interactions (qualitative contact diagrams) and inspect docking poses (https://discover.3ds.com/discovery-studio-visualizer-download)[54]

e) Open Babel (v3.x): Used for file format conversion (e.g., PDB ↔ MOL2 ↔ PDBQT; PDB → XYZ) and atom typing for QM inputs. (https://github.com/openbabel/openbabel/releases)[55]

f) ORCA 5.0.2: Used for cluster-based QM modeling, geometry optimization, and energy calculations (https://orcaforum.kofo.mpg.de/app.php/portal).[56] QM methods and results are described in the corresponding SI sections.

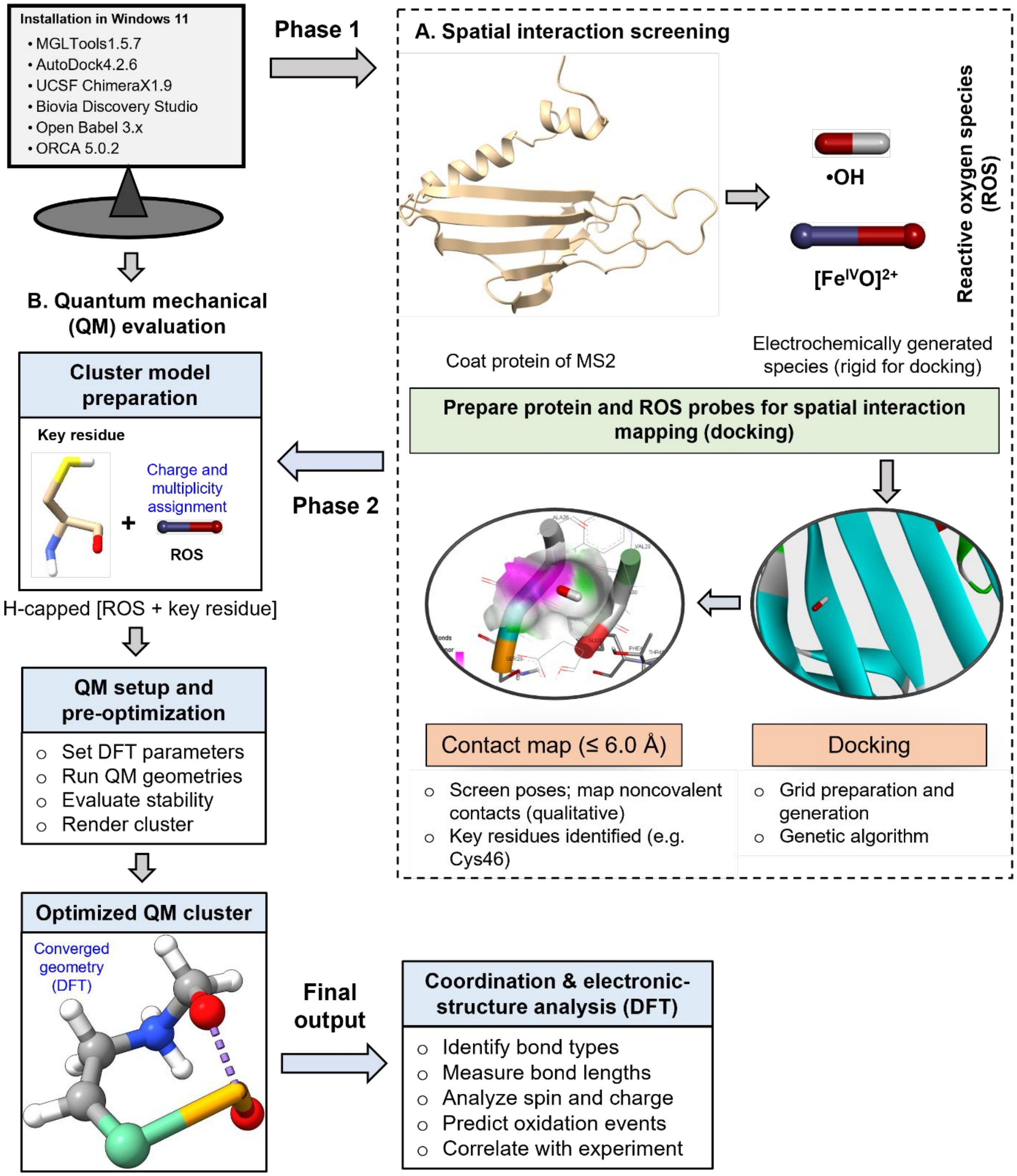

Figure S4. Flowchart of molecular docking (spatial interaction screening; phase 1) and cluster-based modeling using quantum mechanical (QM) modeling (phase 2) workflow.

S7c. Preparation and optimization of models

The three-dimensional (3D) model of MS2 coat protein was downloaded from the Protein Data Bank[57] (https://www.rcsb.org/structure/1MSC). Protonation states were assigned based on physiological pH (6.5 and 5.5), and non-protein components (e.g., bulk waters/ions) were removed for docking grid definition. Missing hydrogens were added, and side-chain orientations were optimized using standard ChimeraX1.9 rotamer libraries; the receptor was kept rigid during docking.

The computed 3D model of $^{\bullet}OH$ (CAS: 3352-57-6; https://webbook.nist.gov/cgi/cbook.cgi?ID=C3352576&Mask=1000) was retrieved from the National Institute of Standards and Technology.[58] For, $[Fe^{IV}O]^{2+}$, 2D structure of the Fe(IV) center was downloaded from PubChem database (CID: 11963629; https://pubchem.ncbi.nlm.nih.gov/compound/Iron_IV),[59] and imported in MolView.[60] In MolView, an O atom was added to the Fe(IV) center to form Fe=O, and input charges were assigned for file generation. Ferryl was represented as an Fe=O unit preset to 1.65 Å (rigid fragment). Subsequently, the ferryl fragment was converted to a 3D molecular geometry. Partial charges were approximated using the Gasteiger method in AutoDockTools 1.5.7 (heuristic; not parametrized for high-valent metal–oxo species), and were used only to generate PDBQT files; they are not interpreted thermodynamically. For spatial-interaction mapping, $[Fe^{IV}O]^{2+}$ and $^{\bullet}OH$ were approximated as neutral Fe–O and O–H fragments simply for geometric assessments; these docking models were not used for energetics or spin-state analysis.

S7d. Docking procedure

AutoDockTools 1.5.7 was used for the preparation of several parameter files – AutoDock4 (AD4/AD4.1 parameter .dat), grid (.gpf), and docking (.dpf). Docking was performed using

AutoDock 4.2 molecular package.[50, 51, 53, 61, 62] The Lamarckian Genetic Algorithm (LGA) with grid boxes centered on the full protein surface to allow unbiased contact-site exploration[61] was used for the docking process with 250 conformational runs. Each grid box was defined with a spacing of 0.375 Å and dimensions of 126 × 126 × 100 grid points (47.3 × 47.3 × 37.5 $Å^3$), centered at (x = 39.622, y = 18.270, z = 17.172 Å) on the MS2 coat protein surface. Mutation and crossover rates were 0.02 and 0.8, respectively. The maximum number of energy evaluation was 2.5 x 10^7. Docked poses were scored using AutoDock's docking score (kcal/mol; heuristic) and AutoDock-estimated inhibition constant (K_i, mM; heuristic). For each species, the highest-ranked pose by docking score was retained as a representative for pose/proximity analysis; scores/ K_i are pose-ranking heuristics only and are not interpreted as binding thermodynamics or reactivity.[51] A complex with the lowest energy and IC was considered to be the most favorable among all the interactions.[51, 63] The best-fitting (top-ranked) pose was analyzed to identify nearby residues using BIOVIA Discovery Studio Visualizer v20.1.0.19295[54] and UCSF ChimeraX v1.9[52, 53]; Discovery Studio interaction labels (e.g., "metal-acceptor," "electrostatic") are software annotations used for qualitative contact visualization and do not imply bonding or energetics. Residues within 6 Å of each ligand in the top-scoring poses were identified for further cluster-based analysis.[64-68]

S7e. Cluster model construction and analysis

A detailed step-by-step description of the density functional theory (DFT) workflow is provided below to ensure reproducibility:

Step-1 – General consideration: While $^{\bullet}OH$ is known for broad reactivity with various amino acids,[45, 69-71] specific interactions involving $[Fe^{IV}O]^{2+}$ with MS2 coat proteins have not yet been characterized. Notably, $[Fe^{IV}O]^{2+}$ is more likely to form and persist under the mildly acidic conditions used here (pH 5.5 and 6.5),[30, 33, 72-75] making it the more relevant and potentially

dominant ROS in our system. Hence in our present study, we have specifically focused on $[Fe^{IV}O]^{2+}$ interaction with coat protein. Also, we have considered only pH 5.5, as docking results gave almost similar interaction pose and sites at both pH 5.5 and 6.5.

Step-2 – Cluster model construction: A capped QM cluster was built around the docked ferryl–Cys46 interface to evaluate local electronic structure and plausible reactivity.

- *Target residue:* Cys46 was considered because of its: (a) proximity with the $[Fe^{IV}O]^{2+}$ (~5.6 Å and Figure S10), (b) thiol reactivity of Cys46[67, 76], and (c) RNA binding implications.[24, 26, 77]
- *Composition:* The model included the $[Fe^{IV}O]^{2+}$ and the side chain of Cys46 (placed at ~2 Å from $[Fe^{IV}O]^{2+}$), with the peptide backbone truncated and capped using hydrogen to preserve the local electronic environment.
- *Coordinates and preparation:* Initial coordinates were extracted from ChimeraX (version 1.9) following molecular docking, and the structure was edited in Windows Notepad to generate .xyz files and input files (.inp) compatible with ORCA5.0.2[56].

Step-3 – Electronic structure settings:

- *Functionals:* Geometries were optimized with B3LYP (Becke three-parameter, Lee–Yang–Parr hybrid) with D3(BJ) dispersion. Energetic/electronic analyses used PBE0 (Perdew–Burke–Ernzerhof hybrid) with D3(BJ).[78]
- *Basis sets:* Geometries employed def2-SVP (split-valence polarized, double-ζ quality) with the geometric counterpoise correction (gCP). Energetic/electronic analyses employed def2-TZVP (triple-ζ valence polarized); and a basis-set convergence probe employed def2-QZVP (quadruple-ζ valence polarized). These levels were chosen for their established performance

in transition–metal–oxo systems (such as oxo-iron(IV) bonding) and compatibility with medium-sized biomimetic clusters.[78]

- *Solvation model:* Solvent response was included at the single-point stage using the Conductor-like Polarizable Continuum Model (CPCM) with the Solvation Model based on Density (SMD), parameterized for water, applied at the PBE0-D3(BJ)/def2-TZVP level on geometries optimized with B3LYP-D3(BJ)/def2-SVP(gCP).[78]
- *Hybrid-DFT acceleration:* The Resolution of the Identity for the Coulomb term (RI-J) and Chain of Spheres for the Exchange term (COSX) or RIJCOSX approximation was tested to accelerate hybrid calculations[78] but led to SCF instabilities for this ferryl–Cys cluster; therefore, all reported data use exact exchange (no RIJCOSX).
- *Code and numerical details:* Calculations used ORCA 5.0.2 (default numerical integration grid Grid4; functional-specific D3BJ parameters; full DFT integral evaluation without resolution-of-identity approximations; TightSCF thresholds: $\Delta E < 1\times10^{-8}$ Eh, density RMS $< 1\times10^{-7}$ a.u.)[56]

Step-4 – Spin-state survey and rationale: For the capped ferryl–Cys cluster ($\sum Z = 90$), the total electron count depends on the overall charge: charge = +2 gives 88 electrons (even), while charge = +1 gives 89 electrons (odd). By the standard parity rule (even electron counts are consistent with odd spin multiplicities; odd counts with even multiplicities), the +2 cluster was examined in triplet (multiplicity = 3, S = 1) and quintet (multiplicity = 5, S = 2) states, which correspond to the canonical d^4 ferryl spin configurations observed in synthetic (S = 1) and non-heme enzymatic (S = 2) systems.[79, 80] The +1 cluster was also tested in the doublet state (multiplicity = 2, S = 1/2) as a control, since an odd-electron cluster could in principle describe antiferromagnetic coupling with ligand-radical character, although this is not the usual ferryl picture. A quartet (multiplicity = 4,

S = 5/2) was not pursued, as it is inconsistent with d^4 ferryl electronic structure and lacks experimental precedent.[46]

Step 5 – Geometry-optimization workflow and diagnostics

- *Sequence:* Optimization was initiated in the doublet (from the docking pose); the optimized doublet geometry then served as the starting structure for triplet and quintet optimizations.
- *Convergence strategy:* Multiple input trials under standard and loose criteria were used to gauge convergence behavior; the final optimized structures satisfied all self-consistent field (SCF) and geometry thresholds. SCF convergence was set to tight.
- *Frequency validation:* For the final quintet, a vibrational frequency analysis at the B3LYP-D3(BJ)/def2-SVP(gCP) level was performed to confirm true minimum i.e. no imaginary modes.
- *Monitored diagnostics (per state):* Orbital occupations, Löwdin charges, spin populations, Mayer bond orders, and energy gaps between highest occupied molecular orbital (HOMO) and the lowest unoccupied molecular orbital (LUMO) (as a multi-reference diagnostic) [56, 81]

Step 6 – Energetic/electronic evaluation and robustness/quality checks

- *Energetic/electronic evaluation:* For each optimized structure, PBE0-D3(BJ)/def2-TZVP calculations were carried out in the gas phase and with CPCM and SMD (water) to quantify relative state energetics (ΔE), spin polarization ($\langle S^2 \rangle$, spin densities), charge distribution (Löwdin)[56, 81], and bonding metrics (Mayer bond orders).[56, 80]
- *Robustness / quality checks:*
  a) Basis-set convergence: a representative quintet CPCM PBE0/def2-QZVP evaluation to confirm negligible differences in spins/charges relative to TZVP.

b) DZ-level BSSE sensitivity: Geometries were re-optimized at B3LYP-D3(BJ)/def2-SVP(+gCP) and re-evaluated at CPCM PBE0/def2-TZVP to confirm numerical shifts were small and spin-state ordering was preserved.

c) Hybrid-DFT stability: RIJCOSX was disabled due to SCF instabilities; all results reflect exact-exchange evaluations.

- <u>*Visualization and reporting:*</u> Optimized structures were visualized with UCSF ChimeraX v1.8 and Discovery Studio. Bond metrics—especially Fe–O (ferryl), Fe–S (ferryl O ↔ Cys46 thiol), etc.—were inspected as reactivity indicators. Convergence metrics (RMS gradients, max step, ΔE) were extracted directly from ORCA outputs.[56] Only chemically reasonable, fully converged geometries were retained for interpretation alongside MALDI-TOF-MS oxidation mass shifts (+16, +32, +48 Da).

S7f. Limitations of the computation modeling approach

The assumptions and limitations associated with each modeling phase are summarized below:

<table>
<tr><th>Component</th><th>Assumptions</th><th>Limitations</th></tr>
<tr><td>Spatial interaction screening (molecular docking; phase-I)</td><td>o Protein structure was treated as rigid during docking
o Reactive species was approximated as a neutral ligand to enable compatibility with classical force fields
o Spatial proximity between the reactive species and nearby residues was used to identify potential oxidation targets for further evaluation</td><td>o Docking does not capture spin state, charge transfer, or covalent bonding
o The oxidation state and radical nature of the reactive species are not represented accurately
o Docking geometry alone cannot confirm oxidative reactivity
o Requires follow-up electronic analysis to assess reactivity potential
o Docking assumed neutral Fe–O and O–H fragments to sample sterically feasible poses; all energetics and spin-state conclusions come from quantum mechanical cluster calculations.</td></tr>
<tr><td colspan="3">The QM phase complemented docking by evaluating electronic structure, radical character, and oxidative potential, addressing reactivity, spin-state, and charge limitations</td></tr>
<tr><td>QM cluster modeling (Phase-II)</td><td>o Built using the geometry derived from docking between the reactive species and a nearby residue</td><td>o The model used continuum solvation (CPCM with SMD, water) only at the single-point stage, omitting explicit</td></tr>
</table>

	o Geometries were optimized in the gas phase, and single-point energies with implicit solvation (CPCM with SMD, water) were computed on the same geometry.<br>o Ground-state spin-state optimization (doublet, triplet, quintet) was used to probe possible electronic configurations	solvent, long-range electrostatics, and surrounding residues.<br>o No reaction pathways, transition states, or oxidation products were modeled.<br>o Represents a static electronic snapshot rather than a full oxidative mechanism.<br>o Does not capture dynamics or conformational effects from the full protein environment.

Section S8. Biochemical analysis using ATR-FTIR

Untreated and coagulated MS2 samples were directly mounted on the diamond accessory which was facilitated with a source (Ever-Glo MIR), beam splitter (KBr), and a detector (DTGS). For each sample, ATR and auto-baseline corrected spectra of 128-averaged coadded scan at 4 cm^{-1} resolutions were further normalized with the highest peak intensity using the Omnic 9 software embedded with the FT-IR system. Spectra were smoothened using 9-points Savitzky-Golay smoothing, and the overlapped peaks were better resolved by considering the second derivative algorithm.

Samples for ATR-FTIR were prepared as detailed in our previous papers.[4, 36, 82] Spectrum corresponding to untreated MS2 sample was the subtracted spectra of pelletized "regular" and "control" MS2 stocks.[4] Regular MS2 stock was prepared as outlined in Section S1, whereas the control sample was prepared similarly as that of regular stock except for the virus inoculation. Similarly, spectrum corresponding to coagulated MS2 samples were obtained by subtracting the spectra of vacuum-filtered suspension (1.2 µm glass filter membrane; dried for 9-12 h) obtained from experiments involving regular and control MS2 stocks. For each coagulated sample, spectrum was collected from three different locations on the filter membrane. Thereafter these spectra were averaged and then the subtraction was performed.

For each coagulated sample, spectra were collected from three different positions on the filter membrane and averaged to obtain one representative spectrum per replicate. Replicate similarity was confirmed by OMNIC QCheck correlation ($r \geq 0.7$). The averaged spectra from three independent experiments were then used for subsequent analysis.

Fraction contribution (F_i; %) and associated standard deviation (SD) of secondary protein structure or carbonyl content was calculated by:

$$F_i = \frac{A_i}{\sum_{i=1}^{i=n} A_i} \quad \text{S18}$$

$$SD(P_k) = \frac{SE(A_i)}{\sum_{i=1}^{i=n} A_i} \quad \text{S19}$$

where,

A_i = fitted area of component i

$SE(A_i)$ = standard error of component i from the fit report

RESULTS AND DISCUSSION

Section S9. Equilibrium speciation calculations

S9a. Protocol

Potential Fe-bearing phases that could control Fe-speciation under different chemical conditions were predicted through thermodynamic modeling of solution saturation state and mineral solubility. Also, non-Fe bearing phases were also identified that could potentially affect the overall reactions under the systems relevant to batch experiments. For these analyses, the updated thermodynamic database was used as detailed in Nilling et al. (2022).[83] The saturation state of a system was evaluated by calculation of saturation index with respect to different Fe-bearing and non-Fe-bearing solids using equation S3.

$$\text{Saturation Index} = \log(\text{IAP}) - \log(\text{K}_{\text{sp}}) \quad \text{(S20)}$$

where, IAP and K_{sp} refer to ion activity and solubility products, respectively. For a specific solid, the solution saturation index I = 0, < 0, or > 0 indicated saturation, undersaturation, or supersaturation, respectively. To identify potential solids at saturation with porewater, the range of saturation index was selected as -2 to 2 [83] to account for propagated errors in saturation index calculations from sampling and analytical errors associated with the data collection and avoid missing any relevant solid phase. Geochemical calculations were performed using Visual MINTEQ 3.1.[84]

Analytes were inputted per the details mentioned in Table S1. Iron was added at a known concentration in the form of either $FeCl_3$ in in case of conventional coagulation or directly input as Fe(II) in electrocoagulation case. In electrocoagulation case, Fe(III)/Fe(II) redox couple was specifically specified in the software at a redox potential (E_H) value of 0.25 V (measured using probe after 60 minutes of experiment). However, in $FeCl_3$ conventional coagulation case iron redox couple was not specified as Fe(III) cannot be reduced to Fe(II) under oxidizing (open-to-air) condition. Solubility plots of iron were developed at a fixed ionic strength of 0.1 M. Dominant solid or aqueous phases at a particular pH and redox potential values (E_H) were identified using predominance diagram plotted using Geochemist's Workbench©.[85]

***S9b*. Equilibrium predictions for $FeCl_3$ coagulation experiments**

All potential Fe(III)-bearing solids remain supersaturated (saturation index > 0) relevant to the experimental condition at both pH 5.5 and 6.5. However, much higher supersaturation was noted at pH 6.5 as compared to pH 5.5 for all Fe(III)-bearing solids indicating higher precipitation chances at pH 6.5 than 5.5 (Table S3). Hematite [$Fe_2O_{3(s)}$] was predicted to be the most favorable

solid at both these pH values followed by maghemite [γ-$Fe_2O_{3(s)}$] and magnesioferrite [$MgFe_2O_{4(s)}$].

Table S3. Saturation index of various Fe(III)-bearing solids relative to the $FeCl_3$ coagulation experimental conditions at pH 5.5 and 6.5 at 20 mg/L iron dosing. Geochemical calculations were performed using Visual MINTEQ 3.1.[84]

Fe(III)-bearing solids	Saturation index[a]	
	pH = 5.5	pH = 6.5
$Fe(OH)_{2.7}Cl_{0.3(s)}$	10.1	11.0
Ferrihydrite [$Fe(OH)_{3(s)}$]	5.9	7.2
Goethite [α-$FeOOH_{(s)}$]	8.7	9.9
Hematite [$Fe_2O_{3(s)}$]	19.7	22.1
H-Jarosite [$KFe_3(SO_4)_2(OH)_{6(s)}$]	7.6	7.2
Lepidocrocite [β-$FeOOH_{(s)}$]	7.8	9.0
Maghemite [γ-$Fe_2O_{3(s)}$]	11.9	14.3
Magnesioferrite [$MgFe_2O_{4(s)}$]	8.5	12.9
Na-Jarosite [$NaFe_3(SO_4)_2(OH)_{6(s)}$]	10.8	11.5

[a] saturation index > 0 represents a solid can precipitate

Solubility plots of potential Fe(III)-bearing solids indicated that hematite and Na/H-Jarosite were respectively the least and the most soluble solids at both pH 5.5 and 6.5 (Figure S5). Nevertheless, the solubility plot clearly suggested that higher solubility of all potential Fe(III)-bearing solids at pH 5.5 as compared to pH 6.5. In other words, at lower pH (5.5), dissolved iron concentrations will be relatively higher than the respective concentrations at pH 6.5. Since the chances of Fe(III) precipitation were more favorable at pH 6.5 than at pH 5.5, higher virus reduction was observed at pH 6.5 in $FeCl_3$ coagulation relative to coagulation at pH 5.5 (Figures 1A,C). Furthermore, the slow kinetics of iron precipitation at pH 5.5 relative to pH 6.5 [86, 87] was also consistent with the geochemical predictions.

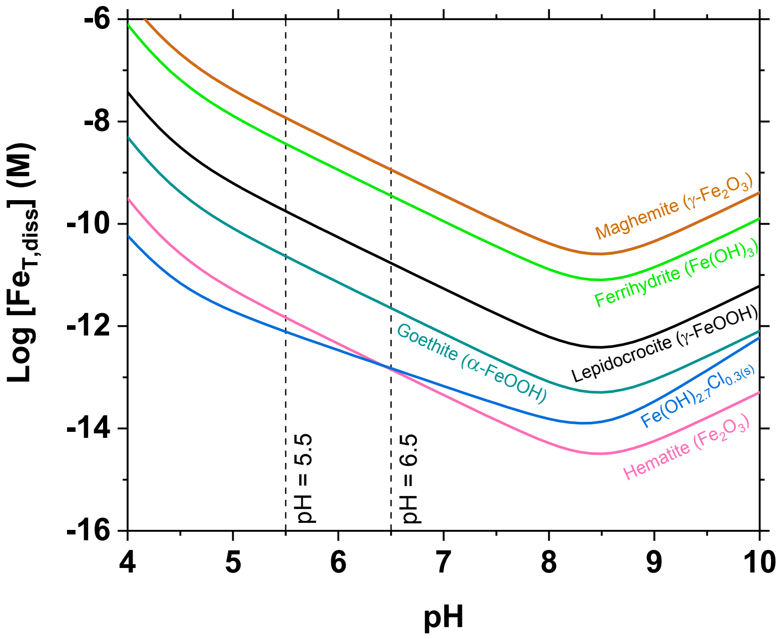

Figure S5. Solubility plots of potential Fe(III)-bearing solids relative to the batch experimental conditions relevant to conventional $FeCl_3$ coagulation. Concentrations of different analytes were added per Table S1 along with 20 mg/L of total iron (added in the form of $FeCl_3$). Geochemical calculations were performed using Visual MINTEQ 3.1[84] at a fixed ionic strength of 0.1 M. Dotted vertical lines at pH 6.5 and 5.5 represent experimental conditions. $Fe_{T,diss}$ represents total dissolved iron concentration.

S9c. Equilibrium predictions for iron electrocoagulation experiments

At pH 6.5, ~100% of total Fe was expected to be precipitated as Fe(III) solids[88-92] (~67%), and a mixture of Fe(II)/Fe(III) mineral (~33%), magnetite [$Fe_3O_{4(s)}$] (Figure S6A and Table S4). However, at the lower pH 5.5, equilibrium iron concentration was distributed as dissolved Fe(II) (~21-84%), precipitated Fe(II) (~5-26%), and precipitated Fe(III) (~10-53%) for varying Fe-dosing (Figure S6B). Due to significant presence of dissolved Fe(II) at pH 5.5 for all iron dosages (5, 10, and 20 mg/L), Fenton reactions would be more favorable at pH 5.5 than at pH 6.5.

The E_H-pH plot indicated the predominance presence of $Fe(OH)_{2.7}Cl_{0.3(s)}$, goethite [α-$FeOOH_{(s)}$], hematite [$Fe_2O_{3(s)}$], magnetite [$Fe_3O_{4(s)}$], and aqueous $FeSO_4$ at pH 6.5 and pH 5.5 (Figure S6C). Results from the predominance plot were consistent with the saturation state calculation.

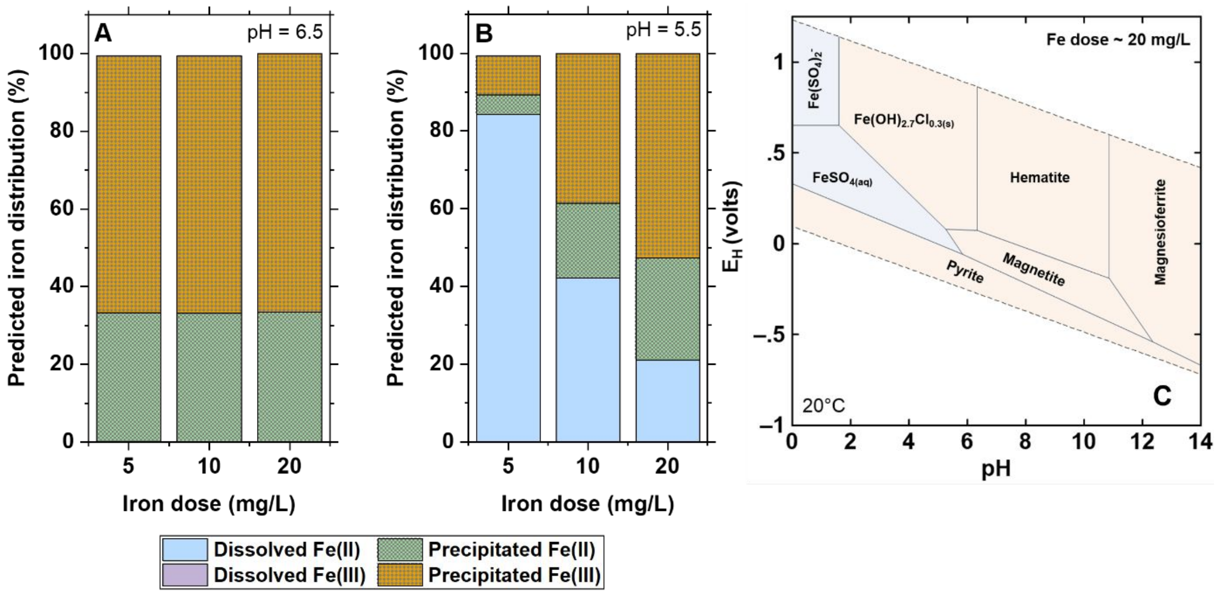

Figure S6. (A, B) Predicted equilibrium iron distribution for iron electrocoagulation systems at different pH and iron dosing. Calculations were performed with an updated thermodynamic database using Visual MINTEQ 3.1.[84] (C) Predominance plot for coagulation system at iron dosing of 20 mg/L. Plot was made using the Geochemist's workbench with an updated thermodynamic database[85]. Predominance plots with different iron dosing (5 and 10 mg/L) were not shown as no major changes were observed.

Table S4. Saturation index of various Fe-bearing solids relative to the iron electrocoagulation batch experimental conditions at pH 5.5 and 6.5 at different iron dosing. Geochemical calculations were performed using Visual MINTEQ 3.1.[84]

Relevant solids	Saturation index[a]					
	pH ~ 5.5			pH ~ 6.5		
	Iron dose (mg/L)					
	5	10	20	5	10	20
**I. Fe-bearing solids**						
$Fe(OH)_{2.7}Cl_{0.3(s)}$	-0.44	-0.13	***0.17***	***2.26***	***2.57***	***2.87***
Goethite [α-$FeOOH_{(s)}$]	-1.66	-1.35	-1.05	***1.34***	***1.65***	***1.95***
Hematite [$Fe_2O_{3(s)}$]	-0.91	-0.30	***0.30***	***5.09***	***5.70***	***6.30***
Lepidocrocite [β-$FeOOH_{(s)}$]	-2.54	-2.23	-1.93	***0.46***	***0.78***	***1.07***
Magnetite [$Fe_3O_{4(s)}$]	***0.22***	***1.13***	***2.03***	***8.22***	***9.13***	***10.03***
**II. Non-Fe bearing solids**						
Chalcedony [$SiO_{2(s)}$]	***0.26***	***0.26***	***0.26***	***0.26***	***0.26***	***0.26***
Cristobalite [$SiO_{2(s)}$]	***0.06***	***0.06***	***0.06***	***0.06***	***0.06***	***0.06***
Quartz [$SiO_{2(s)}$]	***0.71***	***0.71***	***0.71***	***0.71***	***0.71***	***0.71***

[a] "***Bold and italicized***" values in represents a solid can precipitate as saturation index > 0

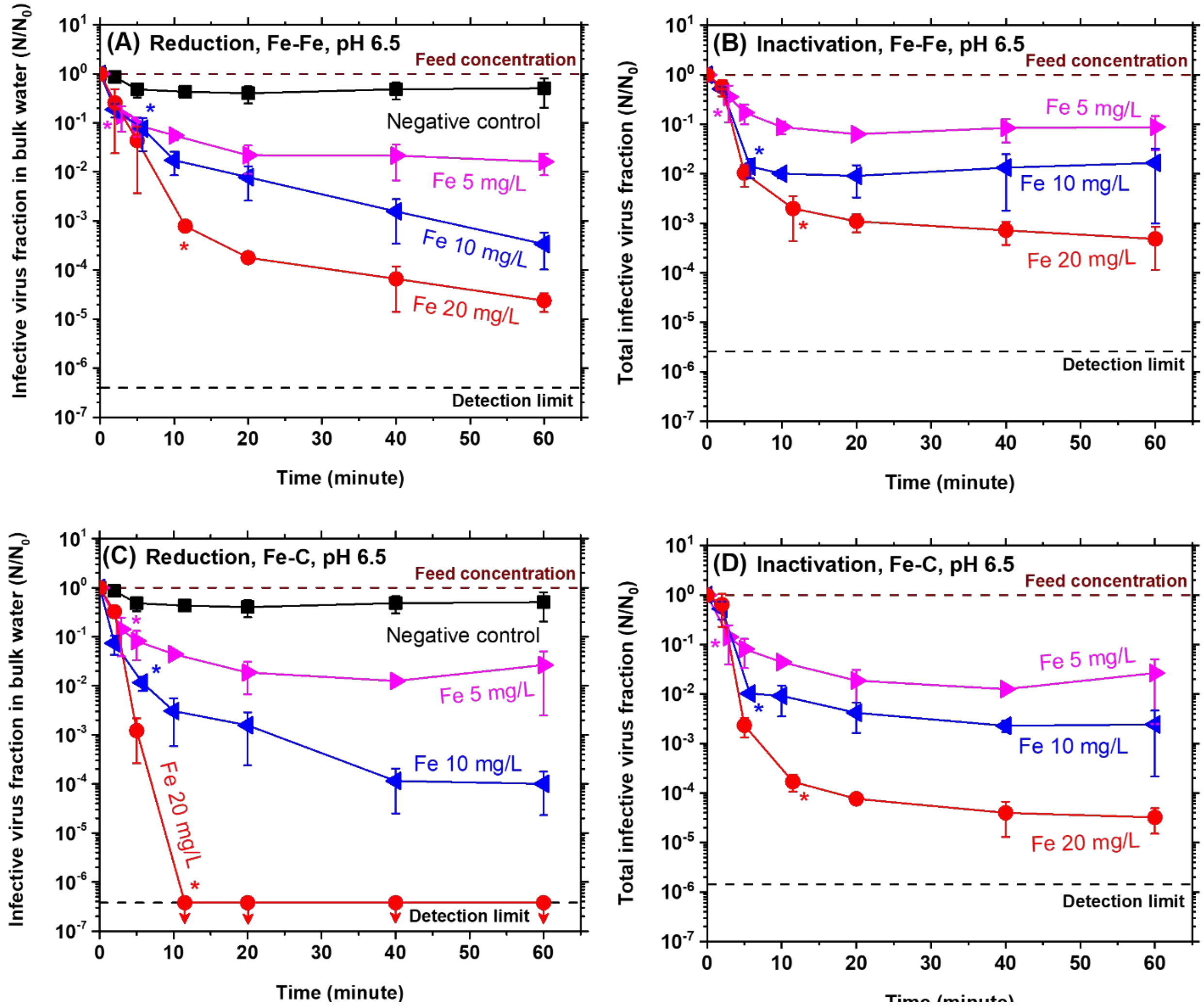

Figure S7. Temporal profile of virus (a, c) reduction and (b, d) inactivation from synthetic water at different pH 6.5 for iron anode and iron cathode electrocoagulation and electrooxidation (Fe-Fe), and iron anode and carbon cathode electrocoagulation and electrooxidation (Fe-C) systems at different iron dosages. For all conditions, the experiments were performed at 1 mA/cm^2 for a total of 1 h, including electrolysis time (as indicated by asterisk (*) mark; 2.9, 5.8, and11.5 minutes corresponding to Fe-dosing of 5, 10, and 20 mg/L, respectively) and flocculation time. Virus (MS2) removal and inactivation was determined from bulk water and suspension (bulk water + flocs), respectively. Data points represent the average of three independent experiments. Error bars denote the standard deviation. The negative control corresponds to virus LRVs measured without current passage.

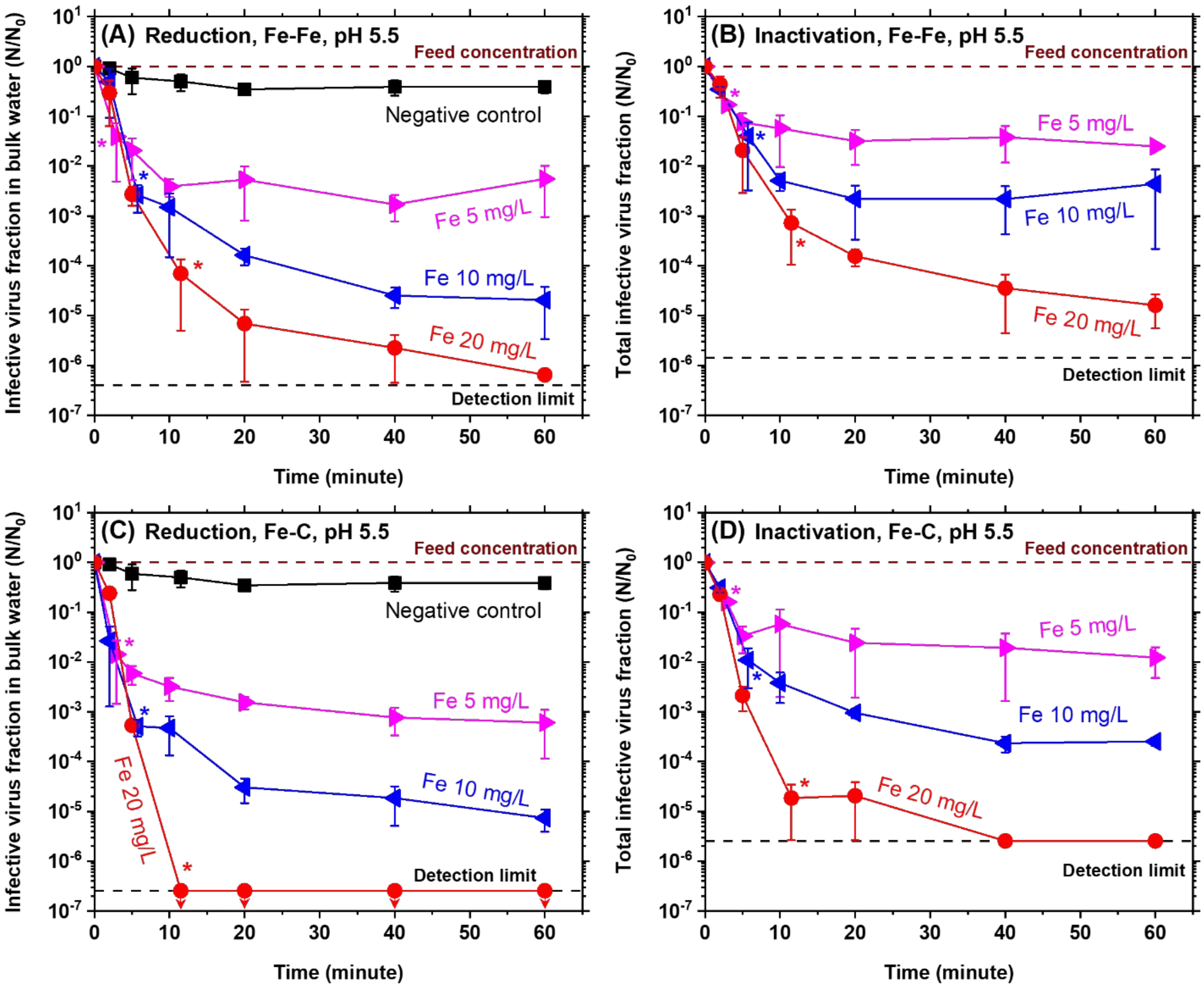

Figure S8. Temporal profile of virus (a, c) reduction and (b, d) inactivation from synthetic water at different pH 5.5 for iron anode and iron cathode electrocoagulation and electrooxidation (Fe-Fe), and iron anode and carbon cathode electrocoagulation and electrooxidation (Fe-C) systems at different iron dosages. For all conditions, the experiments were performed at 1 mA/cm^2 for a total of 1 h, including electrolysis time (as indicated by asterisk (*) mark; 2.9, 5.8, and11.5 minutes corresponding to Fe-dosing of 5, 10, and 20 mg/L, respectively) and flocculation time. Virus (MS2) removal and inactivation was determined from bulk water and suspension (bulk water + flocs), respectively. All data points represent the average of three independent experiments. Error bars denote the standard deviation. The negative control corresponds to virus LRVs measured without current passage.

Section S10. Protein degradation analysis using MALDI-TOF-MS

S10a. Glossary of mass spectrometric terms

1) M: Mass of the intact MS2 coat protein monomer (13,728 Da).
2) Proteolytic damage (protein scission): Formation of cleaved products or truncated protein fragments resulting from backbone cleavage.
3) Non-proteolytic oxidative modification: Chemical modifications of the intact protein detected as mass gains of +16, +32, or +48 Da, corresponding to the incorporation of one, two, or three oxygen atoms, respectively [e.g., (M+H+16)$^+$, (M+H+32)$^+$, (M+H+48)$^+$. These peaks are characteristic of oxidative damage.
4) Non-proteolytic mass-loss modification: Chemical modification of the intact protein associated with a negative mass shift, observed as a -43 Da peak (e.g., M+H-43)$^+$, representing a specific mass-loss variant.
5) Sulfur oxidation products: Oxidation of cysteine residues (e.g., Cys46) to sulfenic (–SOH, +16 Da), sulfinic (–SO_2H, +32 Da), or sulfonic (–SO_3H, +48 Da) acid derivatives, consistent with stepwise oxygen incorporation.
6) Aromatic residue oxidation: Modification of tryptophan (e.g., Trp32) or phenylalanine side chains leading to hydroxylation or higher oxidation products, affecting hydrophobic packing.
7) Backbone carbonylation: Introduction of carbonyl functionalities (aldehydes, ketones) on amino acid side chains, contributing to overall oxidative modification patterns detected in FTIR and MALDI-TOF.
8) RNA-binding patch residues: Specific modifications at Cys46 and Arg49 that may disrupt genome–protein interactions, observed as oxidative or mass-loss variants.

S10b. No protein cleavage at lower m/z region

In the lower m/z region (500–6250), no potential peaks were observed across any of the tested conditions, including after $FeCl_3$, Fe–Fe electrocoagulation, and Fe–C electrocoagulation at pH 6.5 (Figure 9A) and 5.5 (Figure 9B). Signal in this range remained flat and featureless, exhibiting only baseline-level noise without any indication of protein fragments or modification-related ions. This consistent absence of meaningful spectral features across all samples confirms that relevant oxidation products or capsid-derived fragments are not present in this lower mass window.

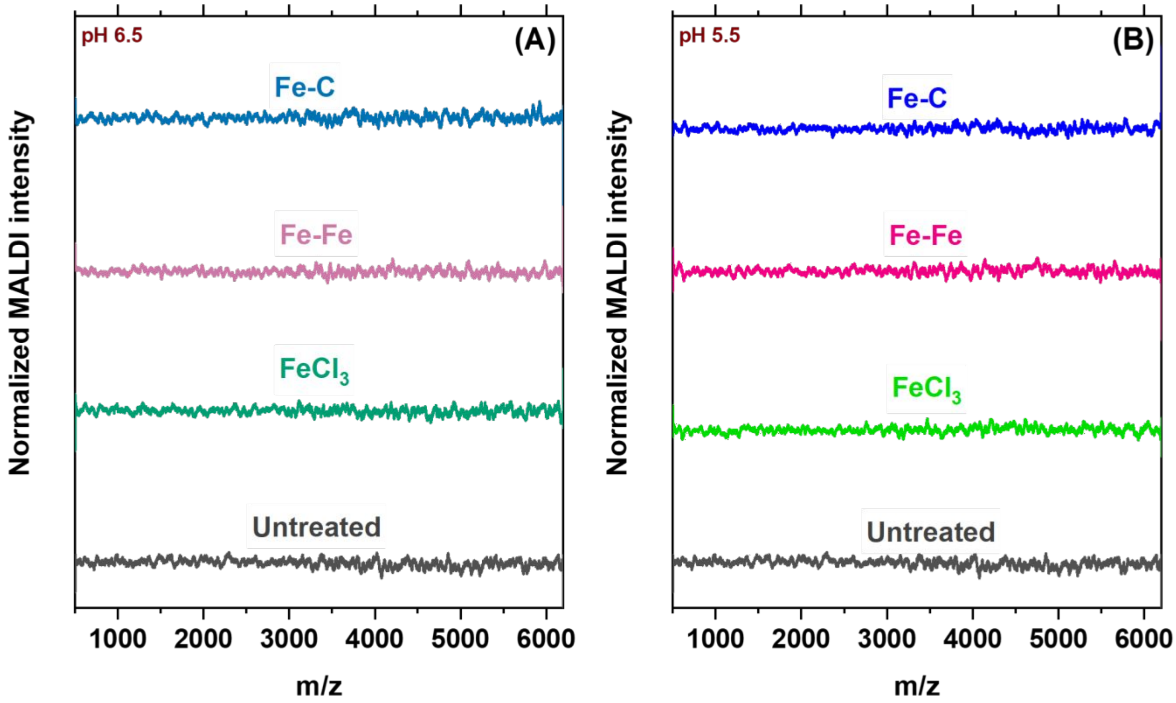

Figure S9. No distinct peaks were observed between m/z 3000-6200 across all samples at pH 6.5 and pH 5.5, and the signal was dominated by random baseline noise.

To confirm that the absence of low-mass peaks in virus samples was not due to limited MALDI-TOF sensitivity, MS2 was digested with trypsin and analyzed under identical instrumental conditions for spectral acquisition. As expected, the resulting spectrum (SI Figure S5) displayed multiple discrete peptide peaks between m/z 500–6,000, consistent with the predicted tryptic fragments of the major coat protein (SI Table S5). Prominent $[M+H]^+$ signals

were observed at m/z ~ 918, 1,077, 1,242, 1,436, 1,559, 1,754, 2,301, 2,614, 4,349 and 4,506 Da. These fragments correspond closely to those reported previously for MS2 tryptic digests,[22, 24] validating both the completeness of the digestion and the resolving power of the instrument.

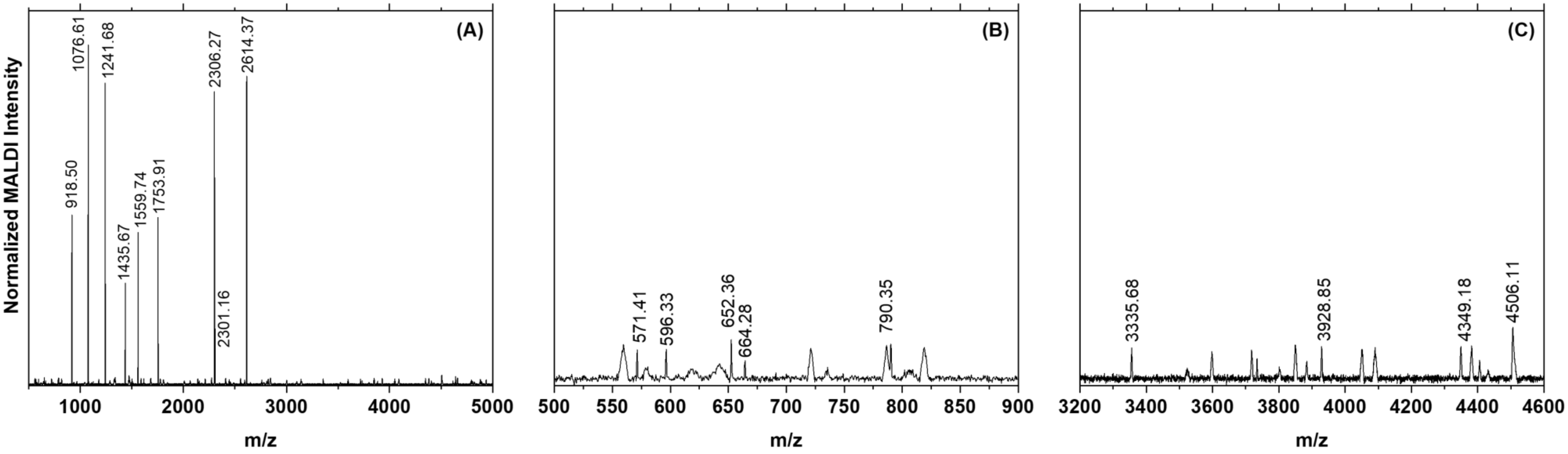

Figure S10. Tryptic-digest MALDI-TOF positive control for the MS2 coat protein. (A) Full spectrum (m/z 500–6000) acquired under the same linear-mode conditions as virus samples. (B) Expanded view of the low-mass region (m/z 500–900). (C) Expanded view of the higher-mass region (m/z 3200–4600). Labeled peaks mark the observed $[M+H]^+$ positions of the major tryptic fragments used for comparison with the predicted list in Table S5. Only major peptide peaks are annotated; other minor signals may correspond to $[M+2H]^{2+}$ species or background

Table S5. Predicted and observed tryptic fragments of the MS2 coat protein.

Predicted m/z	Observed m/z	Start	End	Sequence	Missed cleavage	Comments
4506.16	4506.11	1	43	ASNFTQFVLVDNGGTGDVTVAPSNFANGVAEWISSNSRSQAYK	1	Detected
4349.26	4349.18	67	106	VATQTVGGVELPVAAWRSYLNMELTIPIFATNSDCELIVK	1	Detected
3928.87	3928.85	1	38	ASNFTQFVLVDNGGTGDVTVAPSNFANGVAEWISSNSR	0	Detected
3355.74	3355.68	84	113	SYLNMELTIPIFATNSDCELIVKAMQGLLK	1	Detected
2614.31	2614.37	84	106	SYLNMELTIPIFATNSDCELIVK	0	Detected
2306.29	2306.27	62	83	VEVPKVATQTVGGVELPVAAWR	1	Detected
2301.19	2301.16	107	129	AMQGLLKDGNPIPSAIAANSGIY	1	Detected
1753.96	1753.91	67	83	VATQTVGGVELPVAAWR	0	Detected
1559.77	1559.74	114	129	DGNPIPSAIAANSGIY	0	Detected
1435.71	1435.67	44	56	VTCSVRQSSAQNR	1	Detected

Predicted m/z	Observed m/z	Start	End	Sequence	Missed cleavage	Comments
1241.63	1241.68	39	49	SQAYKVTCSVR	1	Detected
1076.64	1076.61	58	66	YTIKVEVPK	1	Detected
918.48	918.5	50	57	QSSAQNRK	1	Detected
790.38	790.35	50	56	QSSAQNR	0	Detected
760.44		107	113	AMQGLLK	0	Not detected
664.34	664.28	44	49	VTCSVR	0	Detected
652.4	652.36	57	61	KYTIK	1	Detected
596.3	596.33	39	43	SQAYK	0	Detected
571.35	571.41	62	66	VEVPK	0	Detected
524.31		58	61	YTIK	0	Not detected

Note: The amino-acid sequence shown above uses one-letter notation (A = Ala, C = Cys, D = Asp, etc.) following standard proteomics convention. Three-letter notation (e.g., Cys = cysteine, Trp = tryptophan, Tyr = tyrosine, etc.) is used elsewhere in the manuscript for clarity and consistency with biochemical discussions.

S10c. Peak sharpness and preferred peak analysis in MALDI-TOF

In MALDI-TOF analysis of MS2 coat protein, two prominent peaks are typically observed at approximately 6,865 Da and 13,729 Da, corresponding to the $(M+2H)^{2+}$ and $(M+H)^{+}$ ions, respectively. Notably, although both peaks may exhibit similar relative intensities, the peak at 6,865 Da often appears sharper than its 13,729 Da counterpart. This phenomenon is largely attributable to the inherent characteristics of time-of-flight mass spectrometry, where resolution decreases with increasing m/z values.[93, 94] Lower mass ions generally exhibit narrower peak widths due to reduced kinetic energy dispersion and more consistent flight times to the detector.[95] Conversely, heavier ions tend to display broader peaks owing to a greater distribution in their desorption/ionization energies and arrival times. Additionally, the broader 13,729 Da peak may result from minor adduct formations or unresolved isotopic envelopes, especially at higher mass ranges where detector sensitivity and delayed extraction efficiency slightly decline.[93] These effects collectively contribute to the observed differences in peak sharpness between the two species in MALDI-TOF spectra.

While both $(M+2H)^{2+}$ and $(M+H)^{+}$ peaks were visible in the MALDI-TOF spectrum of MS2 coat protein, it is standard analytical practice to focus on the singly charged $(M+H)^{+}$ peak at approximately 13,729 Da for close-scan and high-precision analyses.[24] This preference stems from the fact that the $(M+H)^{+}$ peak represents the intact monomeric form of the coat protein and provides a direct, unambiguous measure of mass changes associated with oxidative modifications or proteolytic cleavage events.[24, 96, 97] Modifications such as oxidation produce a consistent +16 Da shift in the $(M+H)^{+}$ peak, which is easier to resolve and quantify accurately compared to the corresponding +8 m/z shift in the $(M+2H)^{2+}$ peak. Additionally, MALDI-TOF instruments and analytical protocols are typically optimized for singly charged monomers in protein mass

fingerprinting workflows. Also, the observed FWHM or Δm was 11.36 Da indicating oxidation adducts of +8 for $(M+2H)^+$ cannot be accurately determined.

S10d. Spectral reproducibility and deconvolution of MALDI-TOF spectra

Figure S10 confirmed that all replicate spectra showed highly consistent peak distributions and signal intensities across treatments and pH conditions (correlation coefficients 0.95–0.99), indicating that the oxidative mass shifts (+16, +32, +48, −43 Da) observed under Fe–C and Fe–Fe electrocoagulation, as well as $FeCl_3$ coagulation, reflect reproducible treatment-specific modifications rather than analytical variability. This high level of reproducibility supports the reliability of the MALDI-TOF measurements and validates the observed oxidative modifications under different electrochemical and coagulation conditions.

Figure S12 and Table S6 show deconvolution of average MALDI-TOF-MS spectra[41, 42] per the criteria outlined in Section S6c, revealing characteristic mass shifts of +16, +32, and +48 Da—consistent with single, double, and triple oxidation events as well as a -43 Da shift indicative of side-chain fragmentation. These modifications were observed across both pH 6.5 and pH 5.5 conditions, with Fe–C electrocoagulation showing the highest abundance of oxidized species, followed by Fe–Fe EC, while $FeCl_3$ coagulation exhibited comparatively lower levels of oxidative modifications in the viral coat protein (Figure 2C and F).

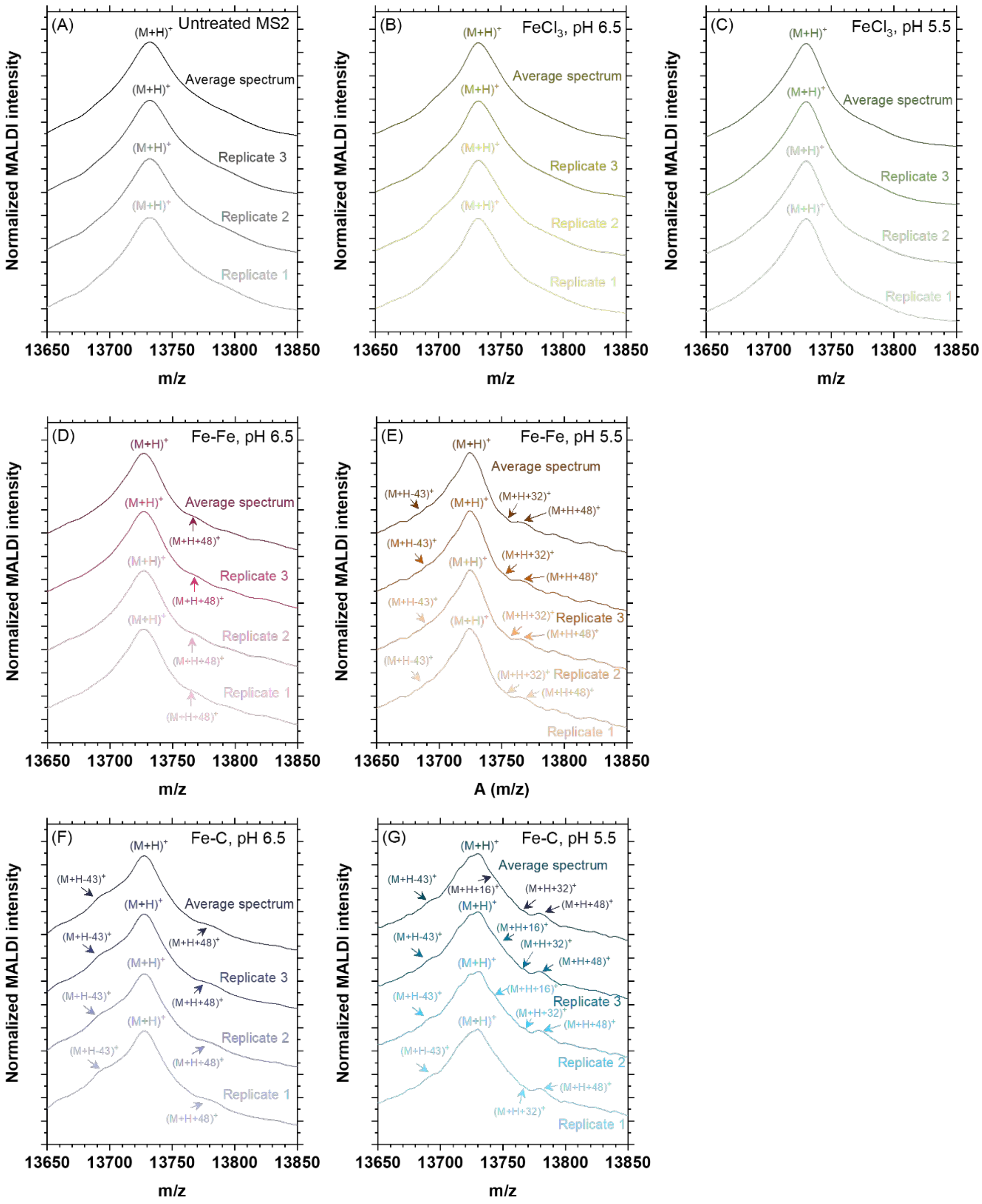

Figure S11. Reproducibility of close-up MALDI spectra (m/z 13650-13850) for (A) untreated, (B, C) iron conventionally coagulated ($FeCl_3$ CC), (D, E) iron-iron electrocoagulated (Fe-Fe EC), and (F, G) iron-carbon electrocoagulated (Fe-C EC) MS2 at pH ~ 6.5 and 5.5. Replicates are 0.90-0.99 correlated with each other.

Table S6. Assignment and relative distribution of oxidation adducts observed in MALDI-TOF-MS. m/z – mass by charge, RA – relative area, $FeCl_3$ – $FeCl_3$ coagulation, Fe-Fe – iron-iron electrode electrocoagulation, Fe-C – iron-carbon electrocoagulation.

Adducts	Untreated MS2		pH ~ 6.5						pH ~ 5.5					
			$FeCl_3$		Fe-Fe		Fe-C		$FeCl_3$		Fe-Fe		Fe-C	
	m/z	RA	m/z	RA	m/z	RA (	m/z	RA	m/z	RA	m/z	RA	m/z	RA
	Da	(%)	Da	(%)	Da	(%)	Da	(%)	Da	(%)	Da	(%)	Da	(%)
$(M+H)^+$	13729	84.2	13731	75.7	13726	46.1	13732	47.7	13728	17.4	13725	21.6	13729	41.6
	13734	15.8	13731	5.3	13727	13.6			13729	80.1	13725	0.8 (53)	13732	2.1
			13731	19.0	13728	5.8			13732	2.5	13726	28.6		
					13731	17.0					13730	1.8		
$(M+H+16)^+$	No peaks detected		No peaks detected						No peaks detected				13743	1.1
													13750	3.2
$(M+H+32)^+$			No peaks detected						No peaks detected		13760	0.2	13758	4.7
											13761	1.5		
$(M+H+48)^+$			No peaks detected		13776	0.5	13781	24.3	No peaks detected		13775	21.5	13758	22.9
					13777	11.2	13782	3.5					13758	8.5
													13758	1.8
$(M+H-43)^+$			No peaks detected		13783	5.5	13687	18.2	No peaks detected		13681	14.3	13691	13.7
							13688	3.5			13683	4.3	13691	0.5

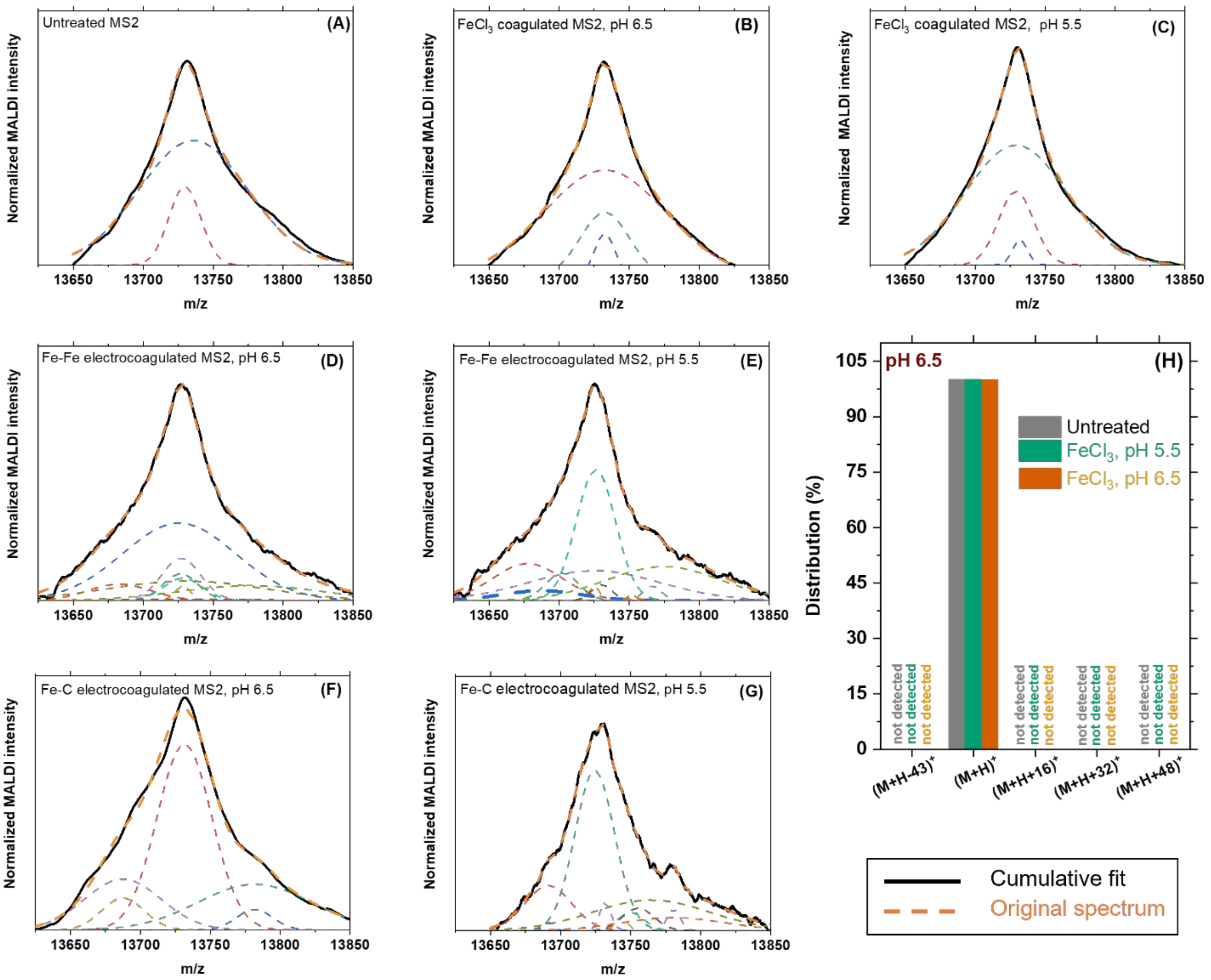

Figure S12. Decomposition of close-up MALDI spectra (m/z 13650-13850) for (A) untreated, (B, C) iron conventionally coagulated ($FeCl_3$ CC), (D, E) iron-iron electrocoagulated (Fe-Fe EC), and (F, G) iron-carbon electrocoagulated (Fe-C EC) MS2 at pH ~ 6.5 and 5.5. (H) Only the $(M+H)^+$ peak contributed to untreated and $FeCl_3$-coagulated viruses at both pH 6.5 and 5.5 Average spectra from three independent runs were deconvoluted.

S10e. Challenges in MS2 coat protein digestion in electrocoagulation systems

We attempted peptide-level digestion and proteomic analysis of MS2 coat protein in electrocoagulation system. However, we faced inherent challenge due to (i) the limited total protein mass available for analysis, despite high initial MS2 titers (~10^9 PFU/mL), and (ii) presence of high concentrations of iron-containing colloids generated post (electro)coagulation. The titer of infective MS2 in our stock was ~10^{12} PFU/mL. For protein analysis, we performed electrocoagulation experiments with minimum possible volume of the electrolyte (i.e., 50 mL). This leads to the low MS2 starting concentrations (10^9 PFU/mL). The problem is further compounded in electrocoagulated MS2 samples. Fe-containing colloids and reactive intermediates were formed that adsorb viral proteins and contribute to secondary reactions during sample concentration and processing.

Proteomic workflows involving enzymatic digestion (e.g., trypsin, chymotrypsin) typically require protein amounts in the low-microgram range to generate reliable peptide maps and MS/MS data with sufficient signal-to-noise and sequence coverage.[98-100] Attempting digestion at sub-optimal protein concentrations risks poor peptide yield, low ionization efficiency in MALDI-TOF-MS or LC-MS/MS, and insufficient detectable peptide fragment coverage. To compensate for low initial viral concentration, especially for (electro)coagulated MS2, we attempted to concentrate and prepare samples via sonication (required for separating protein–colloid complexes) followed by filtration and centrifugation. These attempts of concentrating samples resulted in protein degradation even for untreated MS2.[100] Such degradation not only reduced protein yield, but would have also compromised peptide integrity and complicates downstream interpretation of peptide mass spectra. Peptide-level digestion and proteomic analysis of viral capsid proteins such

as the MS2 coat protein is typically not recommended when starting concentrations are low (e.g., 10^9 PFU/mL), particularly following electrocoagulation treatments.

Section S11. Accessible coat-protein locations and reactive geometries

S11a. Structure-based interaction mapping

Molecular docking identified several surface-exposed regions on the MS2 coat protein that are geometrically accessible to Fe(IV)=O and •OH. Because these species are highly reactive and transient, the docking was used solely as a geometry-based screen (rigid receptor and rigid ligands) to map accessible surface proximity rather than to claim stable binding interactions.[43-46] We therefore report a contact map (≤ 6.0 Å) between probe atoms (e.g., Fe or O•) and specific residue atoms, treating Discovery Studio interaction labels as qualitative. Predicted proximity sites were compared with oxidation signatures observed by MALDI-TOF mass spectrometry, supporting docking as a preliminary accessibility map that helps localize oxidation-prone residues.[47-49] This usage is consistent with prior applications of docking around protein radical centers (e.g., the tyrosyl radical in ribonucleotide reductase).[47-49] Conventional docking scores (Table S7) do not describe covalent modification or electron transfer; we use them only to rank poses consistently. Mechanistic interpretation was developed from quantum chemical cluster calculations (see subsequent sections).

Docking of electrochemically relevant ROS (SI Section S2d) to the coat-protein structure yielded negative AutoDock scoring energies across the models (SI Table S8); we interpret these only for pose ranking, not as thermodynamic or kinetic quantities.[51, 63] Across runs, Fe(IV)=O poses tended to rank slightly better than •OH (more negative scoring energies and lower heuristic K_i), but this is not evidence of stronger binding; rather, it flags geometries with closer approach of the reactive center (e.g., Fe···S(Cys 46) within ≤ 6.0 Å), guiding subsequent QM analysis.

Table S7. Molecular docking analysis of the MS2 coat protein (CP) with electrochemically relevant reactive oxygen species (ROS) during iron electrocoagulation. ROS: hydroxyl radical ($^{\bullet}$OH) and ferryl (Fe(IV)=O). Scoring energies and K_i are heuristic and used only for pose ranking; they are not thermodynamic/kinetic quantities.

**Complex**[$]	ΔG **(kJ/mol)**	**IC (mM)**	**Residues involved in various interactions with ROS**[#]	
			**H-bond, metal acceptor, charge/metal repulsion**	**Nearby residues (< 6Å)**
Coat protein and $^{\bullet}$OH	-7.9	40.0	Asn24, Ala26, Val29	Phe4, Ser23, Phe25, Asn27, Ala30, Glu31, Trp32, Thr45, **Cys46***
Coat protein and Fe(IV)=O	-8.4	36.6	Asn24, Val29, Glu31	Phe4, Ser23, Phe25, Ala26, Asn27, Ala30, Thr45, **Cys46***

[$] Docking models assumed neutral Fe–O and O–H fragments for structural placement only, while energetics and spin states were evaluated using quantum mechanical cluster calculations.
[#] Discovery Studio labels (e.g., H-bond, metal-acceptor, charge/metal repulsion) are qualitative descriptors of noncovalent contacts in the docked geometries.
* Cys46 was selected for further QM investigation due to reasons listed in Section S7e

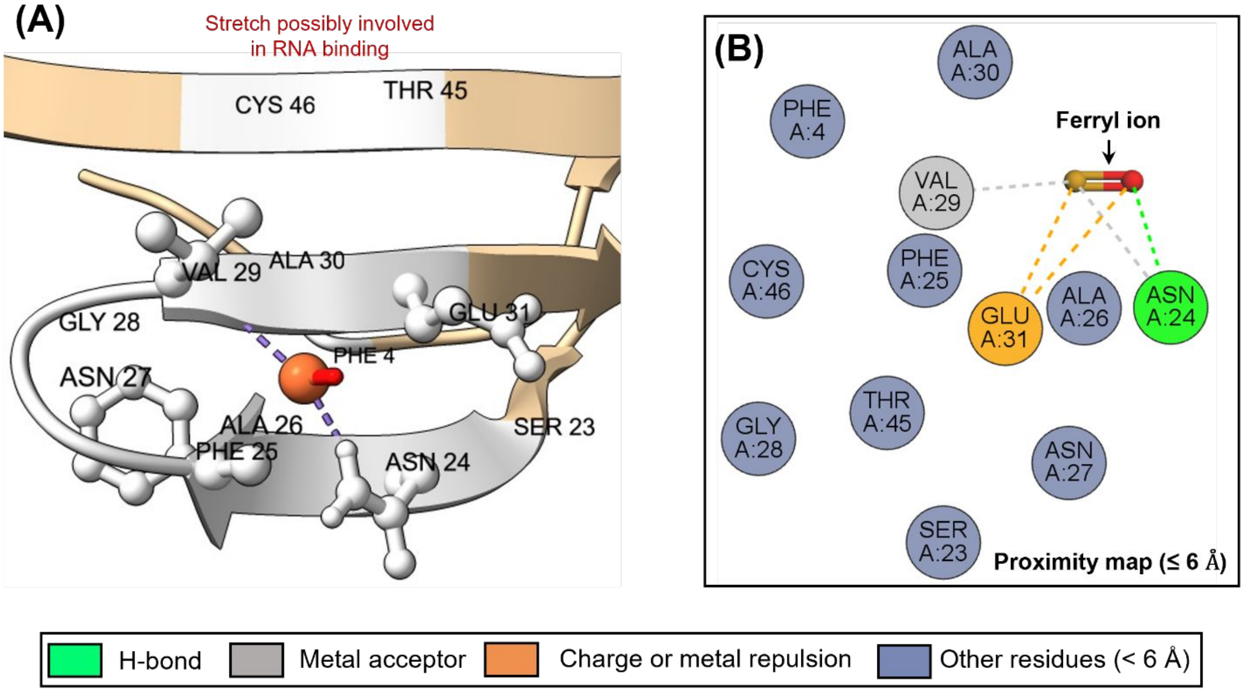

Figure S13. Biomolecular contacts of ferryl ion with the MS2 coat protein. Molecular docking was used solely as a geometry-based screen to identify accessible oxidative sites; predicted proximity was cross-compared with MALDI-TOF results (Figure 2). (A) Enlarged view of the docked region in the rigid protein–ligand complex; dotted-line values are distances (Å). (B) 2D proximity map showing labeled contacts and nearby residues (≤ 6.0 Å). The ferryl ion was modeled as a neutral Fe–O fragment only for spatial interaction mapping. Energetics and spin analysis calculations were performed using DFT and modeled with its correct oxidation state and the appropriate net charge of the QM cluster. Discovery Studio interaction labels are qualitative and docking scoring energies/K*i* are heuristic for pose ranking only (see Table S7). Docking used AutoDock 4.2.6;[50, 53, 61, 62] analysis/graphics used UCSF ChimeraX 1.9[52, 53] and BIOVIA Discovery Studio Visualizer 20.1.0.19295.[54]

Docking of ROS–coat-protein complexes refined the set of potentially affected residues (Figures S13 and S14). Used solely as a geometry-based screen with a rigid receptor and rigid ligands, the analysis reports labeled contacts (H-bond, metal-acceptor, charge/metal repulsion) and nearby residues (≤ 6.0 Å), with Discovery Studio labels treated as qualitative. H-bonds are typically stronger than dipole–dipole and van der Waals contacts, but this ordering was used only qualitatively.[101] The ROS probes were placed toward the N-terminal region of the coat protein—Ser23–Cys46 and Phe4—consistent with our accessibility mapping (Figures S13 and S14). Across the docked complexes, several residues recurred, but the interaction types differed by ROS, as seen

in the best-ranked poses (selected by more negative AutoDock scoring energy ΔG and lower heuristic K_i; Table S7).

For Fe(IV)=O, Asn24 formed the only labeled H-bond; additional labels included metal-acceptor contacts with Asn24 and Val29, and charge/metal repulsion with Glu31 (Figure S13B). For •OH, labeled H-bonds involved Asn24, Ala26, and Val29 (Figure S14B). In both docked complexes, several residues—Phe4, Ser23, Phe25, Asn27, Ala30, Thr45, and Cys46—occur within ≤ 6.0 Å of the probe (Table S8).[102]

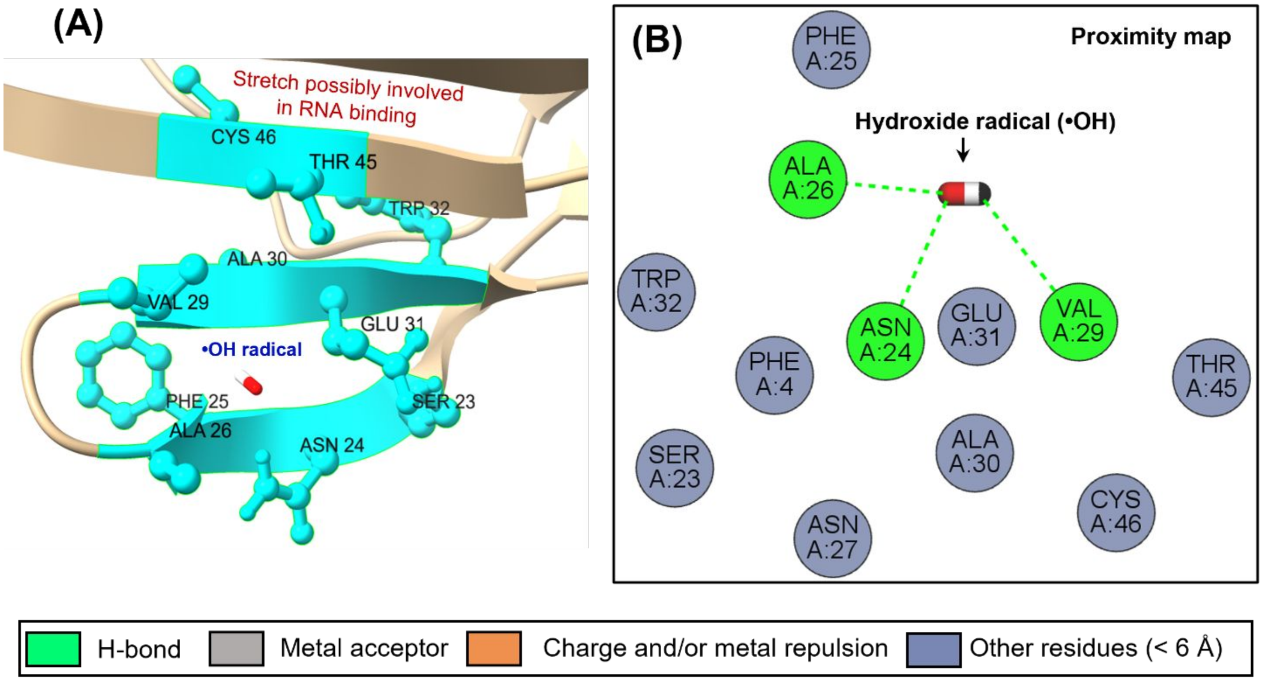

Figure S14. Biomolecular contacts of hydroxyl radical with the MS2 coat protein. Molecular docking was used solely as a geometry-based screen to identify accessible oxidative sites; predicted proximity was cross-compared with MALDI-TOF results (Figure 2). (A) Enlarged view of the docked region in the rigid protein–ligand complex; dotted-line values are distances (Å). (B) 2D proximity map showing labeled contacts and nearby residues (≤ 6.0 Å). Discovery Studio interaction labels are qualitative, and docking scoring energies/K*i* are heuristic for pose ranking only (see Table S7). Docking used AutoDock 4.2.6;[50, 53, 61, 62] analysis/graphics used UCSF ChimeraX 1.9[52, 53] and BIOVIA Discovery Studio Visualizer 20.1.0.19295.[54] Hydroxyl radical was modeled as a neutral O–H fragment only for spatial interaction mapping.

For the •OH –coat-protein and Fe(IV)=O–coat-protein complexes, Phe4, Ser23, Asn24, Phe25, Ala26, Asn27, Val29, Ala30, Glu31, Thr45, and Cys46 were either labeled as interacting (H-bond,

metal-acceptor, or charge/metal repulsion) or occurred as nearby residues (≤ 6.0 Å; Figures. S13B, S14B; Table S7). Among these residues, Ser, Asn, Thr, and Cys are polar amino acids,[103] typically localized on the outer surface of the coat protein.[77, 104, 105] Consistent with this, Ser23, Asn24, Thr45, and Cys46 are likely more accessible—and thus more susceptible—to $^{\bullet}$OH and Fe(IV)=O. Notably, Thr45 and Cys46 are implicated in RNA binding.[24, 77] Interactions of these ROS at such sites could contribute to MS2 inactivation during iron electrocoagulation.

Alongside Cys46, Trp32 (predicted in the $^{\bullet}$OH complex) can also undergo multiple oxidations—e.g., hydroxytryptophan and N-formylkynurenine.[106] Although Trp32 is non-polar,[103] these products are well documented. Together, Trp32 and Cys46 (the latter with higher plausibility) provide a mechanistic link to the observed +16, +32, and +48 Da MALDI shifts during iron electrocoagulation at pH 6.5 and 5.5 (Figures 2B, 2E, and 3), which we evaluate next via cluster-based DFT. We therefore focused on Cys46 for the QM cluster analyses (Sections S7e and S11b).

Table S8. Closest approach of ferryl ion ($[Fe^{IV}O]^{2+}$) and hydroxyl radical ($^{\bullet}$OH) to MS2 coat protein residues identified via docking analysis. Distances were computed using chimerax1.9[52, 53]

**$[Fe^{IV}O]^{2+}$ interaction with protein**		**$^{\bullet}$OH interaction with protein**	
**Residue**	**Distance(Å) (nearest atom)**	**Residue**	**Distance(Å) (nearest atom)**
Asn24	2.002 (O)	Ala26	1.83 (HN)
Val29	2.047 (O)	Asn24	2.2 (2HD2)
Ala26	2.110 (HN)	Val29	2.58 (O)
Glu31	2.223 (HN)	Glu31	3.05 (CB)
Phe25	2.750 (CA)	Phe25	3.44 (N)
Ala30	2.913 (CA)	Ala30	3.9 (C)
Phe4	4.638 (CZ)	Ser23	5.59 (OG)
Cys46	5.635 (HN)	Asn27	5.64 (N)
Ser23	5.664 (HN)	Phe4	5.81 (CZ)
Asn27	5.708 (N)	Thr45	5.99 (OG1)
Gly28	5.902 (C)	Cys46	6.08 (HN)
Thr45	6.266 Å (HG1)	Trp32	6.25 (N)
Arg49	13.301 (N) – not in binding site	Gly28	6.51 (C)
Arg38	18.956 (HN) – not in binding site	Arg49	13.9 (N) – not in binding site
		Arg38	18.29 (HN) – not in binding site

HN: Backbone amide hydrogen, CA: Alpha carbon, O: Backbone carbonyl oxygen, N: Backbone nitrogen, SG: Sulfur atom in cysteine side chain, CB: Cβ carbon of sidechain, OG: Sidechain hydroxyl oxygen, CZ: Aromatic carbon in phenyl ring, 2HD2: Sidechain hydrogen on nitrogen (e.g., in Arg),

Section S11b. Cluster-based modeling using QM approach

To evaluate the local electronic environment and reactivity of the $[Fe^{IV}O]^{2+}$ center in proximity to Cys46, we constructed a capped cluster model and carried out geometry optimizations across three spin states: doublet (S = ½), triplet (S = 1), and quintet (S = 2). All optimizations were performed at the B3LYP-D3(BJ)/def2-SVP level of theory, using coordinates derived from the docking pose. The resulting geometries were then subjected to single-point (SP) energy calculations and electronic structure analyses at the higher PBE0-D3BJ/def2-TZVP level. Convergence profiles are shown in Figure S15, which includes six panels: (A–C) optimizations without geometric counterpoise (gCP) for the doublet, triplet, and quintet; and (D–F) the corresponding optimizations repeated with gCP [B3LYP-D3(BJ)/def2-SVP(gCP)]. Detailed numerical results—including SCF energies, $\langle S^2 \rangle$ values, spin populations, bond lengths, and bond orders—are summarized in Table S9. CPCM results (HOMO–LUMO gaps) are summarized below and discussed alongside the gas-phase metrics. Frequency analysis at the B3LYP-D3(BJ)/def2-SVP(gCP) level confirmed no imaginary modes for the quintet cluster (true minimum; ν(Fe=O) = 947.5 cm^{-1}). The corresponding zero-point vibrational energy was obtained from the vibrational analysis.

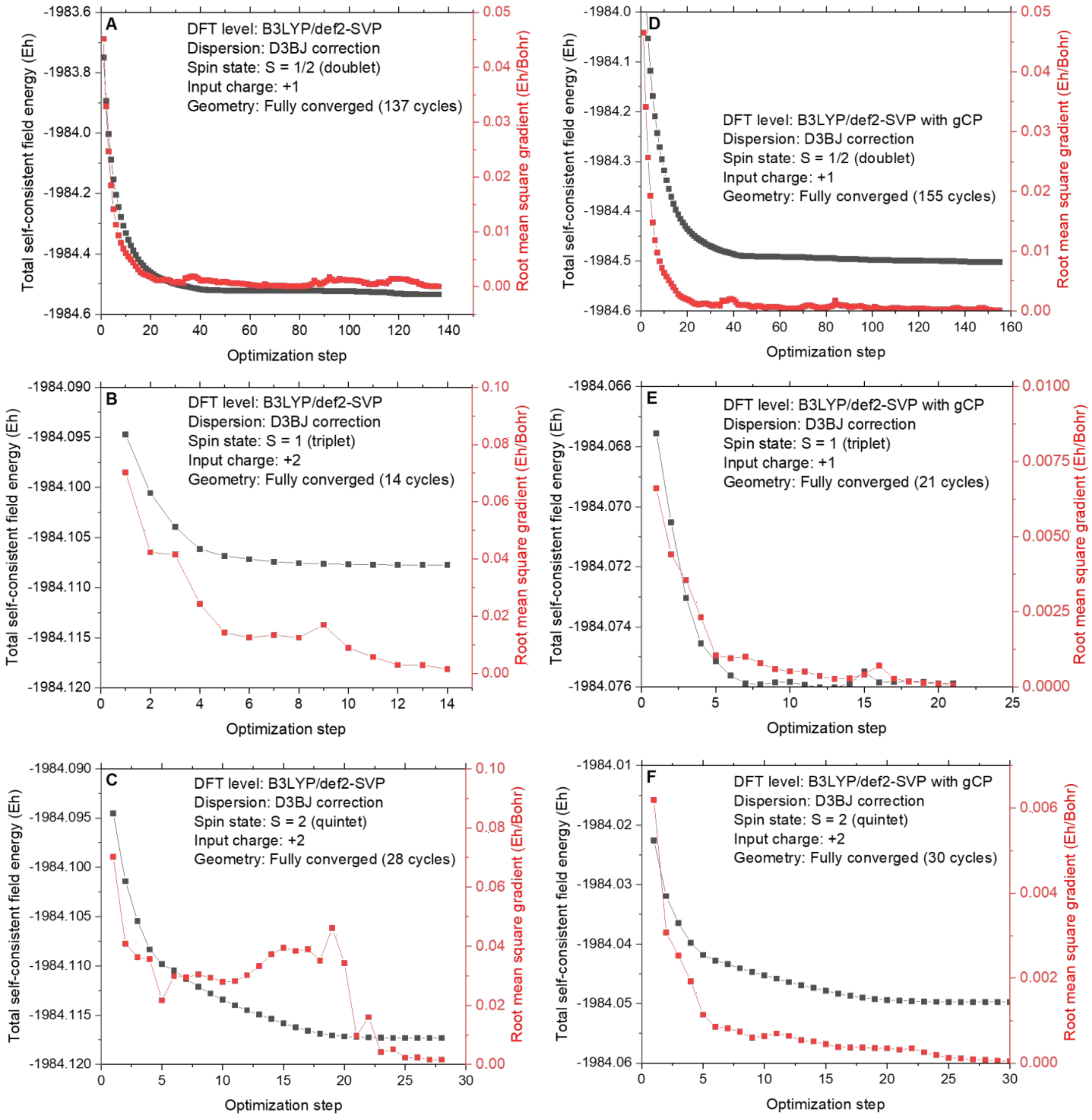

Figure S15. Geometry optimization convergence profiles for the ferryl–Cys46 cluster under six conditions: (A) doublet (charge = +1, multiplicity = 2), (B) triplet (charge = +2, multiplicity = 3), and (C) quintet (charge = +2, multiplicity = 5) without geometric counterpoise (gCP) correction; and (D)–(F) the corresponding optimizations repeated with gCP [B3LYP-D3(BJ)/def2-SVP(gCP)]. Optimizations were initiated for the doublet spin state from the docking-derived coordinates, and the converged doublet structure was subsequently used as the starting geometry for the triplet and quintet runs. Each panel shows the self-consistent field (SCF) total electronic energy (left y-axis, black) and the root-mean-square (RMS) Cartesian gradient (right y-axis, red) as a function of optimization step. The resulting geometries were further analyzed by higher-level single-point calculations at PBE0-D3(BJ)/def2-TZVP, with additional CPCM(water, ε = 78.4) solvation applied to the gas-phase geometries. Inclusion of gCP did not materially alter the optimization trajectories or final structures, confirming that the mechanistic conclusions are unaffected by applying geometric counterpoise correction at the double-ζ geometry level.

Doublet configuration: In the doublet spin state (Figure S15A), the system converged over 137 geometry steps. The corresponding single point (SP) energy was –1984.76 Eh (Table S9), numerically the lowest among the three configurations. However, the calculated ⟨S^2⟩ value was 1.63, substantially higher than the expected 0.75, indicating ~117% spin contamination. This suggested mixing with higher spin states, which undermined confidence in the wavefunction's physical validity. Under CPCM solvation, the contamination worsened to ~268%, confirming that the doublet wavefunction was fundamentally unreliable. The doublet state was not considered reliable for further mechanistic interpretation due to this level of spin contamination, and also due to the increased energy (-1984.44 Eh) in the solvated phase. The Mayer bond orders further reinforced this limitation: Fe=O (1.675) and Fe–S (0.752) were reasonable, but not the Fe···O(carbonyl) contact, which was unusually high (0.795), suggesting artificial bonding contributions from spin contamination. In solvent, Fe···O dropped to ~0.481 while Fe=O and Fe–S remained nearly unchanged, highlighting that the apparent secondary coordination in gas was not robust. In solvent, Fe···O dropped to ~0.481 while Fe=O and Fe–S remained nearly unchanged, highlighting that the apparent secondary coordination in gas was not robust (Table S9). Under CPCM (water), the smallest spin-channel HOMO–LUMO gap for the doublet was ~2.89 eV, which is narrower than in the gas phase (~3.35 eV) but does not mitigate the severe spin contamination. With gCP (Figure S15D), the optimization converged in 155 steps with a final RMS gradient of ~5.16×10^{-5} Eh $Bohr^{-1}$ and a final SCF energy of -1984.50 Eh. Importantly, the bonding metrics and electronic features of the gCP geometry were essentially indistinguishable from the no-gCP case, confirming that applying gCP does not alter the interpretation.

Triplet configuration: The triplet state (Figure S15B) converged in 15 optimization steps. The SP energy was -1984.33 Eh, and the ⟨S^2⟩ value was 2.0611, indicating mild contamination (~5%)

in the gas phase (Table S9). Although, under CPCM solvation, energy change was minimal (-1984.33 Eh), spin contamination rose dramatically to ~53%, undermining its apparent gas-phase stability. The spin population on the Fe center was 1.84, lower than would be expected for a strongly oxidizing ferryl species, and the energy was higher than both the doublet and the quintet. The Mayer bond orders reflected this: the Fe=O was strongest (1.983) at the shortest bond length (1.545 Å), but the Fe–S remained relatively covalent (0.901), inconsistent with the expected polarization of the Cys thiolate. In solvent, Fe···O collapsed from 0.953 to ~0.394, showing that much of the secondary coordination was environmentally fragile. Thus, although the triplet appeared electronically "cleaner" than the doublet in gas phase, its severe solvation-driven contamination and bonding pattern make it chemically unreliable. With CPCM (water), the α-channel gap narrowed to ~2.30 eV (β-channel ~4.39 eV), giving the triplet the smallest spin-channel gap among the three states in solvent. Because gap alone is not a sufficient criterion—and the Fe(IV)=O radical character remains subdued in the triplet—this did not change our state selection. With gCP (Figure S15E), the optimization converged in 21 steps with a final RMS gradient of ~5.21×10^{-5} Eh $Bohr^{-1}$ and a final SCF energy of -1984.08 Eh. The gCP trajectory led to the same qualitative outcome as the no-gCP run, underscoring that the triplet's limitations arise from intrinsic spin contamination rather than basis-set superposition.

Quintet configuration: The quintet spin configuration (Figure S15C) converged within 29 steps and produced an SP energy of –1984.34 Eh (gas) and –1984.48 Eh (solvent) (Table S9). The calculated $\langle S^2 \rangle$ value of 6.0736 was very close to the ideal value of 6.00, with minimal spin contamination (~1.7%) making it the only state with consistently negligible contamination across environments. Löwdin analysis gave Fe a partial charge of +0.36 with a spin density of 2.75; the terminal oxo carried a charge of –0.26 and spin density of 0.20; and the Cys sulfur carried a charge

of +0.66 and spin density of 0.04, consistent with a high-spin Fe(IV)=O radical and a polarized Cys thiolate. A spin density of ~0.8 was also localized on the cysteine backbone carbon, reflecting delocalization through the Fe–S–Cys framework and secondary orbital coupling with the backbone carbonyl rather than direct radical activity (SI Table S9). Mayer analysis aligned best with chemical expectations: Fe=O was strong (1.894), Fe–S was clearly weakened (0.702), and Fe···O was moderate but real (1.037). In solvent, Fe–O and Fe–S remained stable (1.750, 0.679), while Fe···O dropped to ~0.390, consistent with attenuation of only the weaker secondary interaction. This robustness across both gas and CPCM conditions underscored the quintet as the most chemically and physically reasonable state. Furthermore, the quintet state displayed the smallest HOMO–LUMO gap (highest occupied molecular orbital and lowest unoccupied molecular orbital; ~3.2 eV), consistent with a more reactive electronic configuration compared to the triplet (~4.1 eV) and doublet (~3.4 eV). This, along with lower SCF energy and acceptable spin properties, supports selection of the quintet solution for mechanistic interpretation. These features—summarized in Table S9—supported partial covalent bonding and electronic redistribution that would favor oxidative sulfur chemistry. In the solvent phase (CPCM, water), the smallest spin-channel gaps were: doublet ~2.89 eV; triplet ~2.30 eV (α); and quintet ~3.62 eV (α). Gas-phase values remain as listed in Table S9, and the solvent shifts do not alter the qualitative spin-state assignment—the quintet remained favored. With gCP (Figure S15F), convergence was achieved with virtually the same step count and a final SCF energy of -1984.34 Eh. Bonding and electronic descriptors were unchanged relative to the no-gCP case, reinforcing the robustness of the quintet assignment.

Table S9. Comparative analysis of different spin configuration for the ferryl–Cys46 cluster model

Property	Gas phase *(in vacuo)*			Implicit solvent or solvated phase		
	**Doublet**	**Triplet**	**Quintet**	**Doublet**	**Triplet**	**Quintet**
A. General input parameters						
Charge	+1	+2	+2	+1	+2	+2
Multiplicity (spin)	2 (S = 1/2)	3 (S = 1)	5 (S = 2)	2 (S = 1/2)	3 (S = 1)	5 (S = 2)
Geometry level	B3LYP-D3BJ/def2-SVP with gCP					
Energetic level	PBE0-D3BJ/def2-TZVP			PBE0-D3BJ/def2-TZVP + CPCM with SMD (water)		
B. Model quality						
SCF energy (Eh)[a]	-1984.76	-1984.33	-1984.34	-1984.44	-1984.37	-1984.48
ΔE (kcal/mol; vs quintet)	-263.6	6.3	0.0 (reference)	25.1	69.0	0.0 (reference)
Ideal $\langle S^2 \rangle$[b]	0.75	2	6	0.75	2	6
Calculated $\langle S^2 \rangle$[b]	1.63	2.1	6.1	2.76	3.1	6.1
Error in $\langle S^2 \rangle$ (%)	117.3 (severe)	5.0 (minimal)	1.7 (negligible)	268.1 (severe)	52.7 (severe)	1.7 (negligible)
C. Spin population (Löwdin analysis)[56, 81]						
Fe (ferryl)	1.30	1.84	3.27	2.79	2.68	2.75
O (ferryl)	-0.37	0.32	0.36	-0.05	0.25	0.20
S (Cys46)	-0.02	-0.09	0.15	-0.16	-0.03	0.04
O (Cys46)	0.06	-0.05	0.18	-0.01	-0.17	0.13
C (C=O, Cys46)	0.021	-0.010	-0.009	-0.19	-0.007	-0.005
C (Cα, Cys46)	0.003	-0.002	-0.001	-0.042	-0.013	0.007
N (peptide)	0.002	0	0.009	-0.235	-0.019	0.026
C (backbone, cys46)	0.004	-0.002	0.002	-0.432	-0.592	0.800
H (backbone, cys46)	*Insignificant*					
D. Partial charge (Löwdin analysis)[56, 81]						
Fe (ferryl)	0.14	0.32	0.55	0.19	0.23	0.36
O (ferryl)	-0.27	-0.06	-0.10	-0.29	-0.24	-0.26
S (Cys46)	0.71	0.86	0.76	0.64	0.72	0.66
O (Cys46)	-0.02	0.06	0.03	0.03	0.28	0.35
C (C=O, Cys46)	-0.36	-0.30	-0.33	-0.42	-0.35	-0.34
C (Cα, Cys46)	-0.27	-0.23	-0.22	-0.26	-0.24	-0.21
N (peptide)	0.18	0.22	0.21	0.12	0.22	0.24
C (backbone, cys46)	-0.35	-0.29	-0.30	-0.14	-0.20	-0.06
H (amide H)	0.17	0.20	0.19	0.13	0.23	0.19

Property	Gas phase *(in vacuo)*			Implicit solvent or solvated phase		
	**Doublet**	**Triplet**	**Quintet**	**Doublet**	**Triplet**	**Quintet**
H (Cα, H)	0.19	0.21	0.20	0.15	0.19	0.20
H (methylene H1)	0.18	0.20	0.20	0.23	0.25	0.20
H (methylene H2)	0.22	0.25	0.22	0.19	0.31	0.27
H (capping H1)	0.21	0.24	0.23	0.19	0.22	0.25
H (capping H2)	0.14	0.17	0.17	0.18	0.25	0.13
H (capping H3)	0.13	0.16	0.17	0.06	0.14	0.02
HOMO-LUMO gap (eV)[d]	3.35	4.06	3.17	2.79	2.30	3.62
E. Bond lengths (Å) and bond orders						
Lengths						
Fe=O	1.549	1.538	1.587	1.549	1.538	1.587
Fe–S (Cys)	2.172	2.307	2.482	2.172	2.307	2.482
Fe···O (Cys)	1.806	1.745	1.776	1.806	1.745	1.776
Orders						
Fe=O	1.675	1.983	1.894	1.616	1.810	1.750
Fe–S (Cys)	0.752	0.901	0.702	0.748	0.804	0.679
Fe···O (Cys)	0.7956	0.953	1.037	0.481	0.394	0.390
Remarks	Lowest electronic energy, but severe spin contamination (unreliable)	Higher energy than doublet or quintet, mild contamination (acceptable but less stable)	Slightly higher energy than doublet but cleanest spin, consistent charges/spins (mechanistically most interpretable)	Still severe spin contamination, distorted spin/charge distribution (unreliable)	Strong contamination, unstable distribution (unreliable)	Lowest solvated energy, minimal contamination, consistent spin localization on Fe/O, supports oxidative reactivity (Best)

[a] SCF: Self-consistent field. Measured in Hartree energy (Eh). 1 Eh = 2625.5 kJ/mol

[b] ⟨S^2⟩ is the expectation value of the total spin angular momentum operator**,** representing the average squared spin angular momentum of a quantum system. It is calculated as ⟨S^2⟩ = S(S+1), where S is the spin quantum number

[c] bond lengths between two atoms A and B with 3D coordinates are calculated using:

$$\text{Bond length (Å)} = \sqrt{(x_B - x_A)^2 + (y_B - y_A)^2 + (z_B - z_A)^2}$$

where, x, y, z are the Cartesian coordinates of atoms A and B

[d] Energy difference between the highest occupied molecular orbital (HOMO) and the lowest unoccupied molecular orbital (LUMO) was also monitored as a diagnostic of electronic stability:

Spin-state outcomes: As outlined in the Section S7e, the doublet (+1) was examined as a parity-consistent control, while the triplet and quintet (+2) were tested as the canonical d^4 ferryl states. The doublet optimization ultimately showed severe spin contamination and ligand-radical character, the triplet was moderately clean in gas phase but unstable under solvation, and only the quintet retained the expected high-spin ferryl character across all environments. When all energies were expressed relative to the quintet reference (Table S9), the gas-phase doublet appeared artificially lower by ~264 kcal/mol (an artefact of contamination), whereas in solvent it was ~+25 kcal/mol above the quintet; the triplet lies ~+6 kcal·mol^{-1} (vacuum) and ~+69 kcal/mol (solvent) higher. These comparisons reinforced the quintet as the most stable and chemically reasonable state.

Overall recommendation and other checks: Together, data in Table S9 suggested that the quintet state offered the most balanced and physically meaningful representation of the ferryl–thiolate cluster. While the doublet exhibited the lowest computed energy in gas phase, its catastrophic spin contamination precluded confident interpretation. The triplet had improved spin purity in gas phase but collapsed under solvation with severe contamination, making it unreliable. The quintet, by contrast, showed consistent behavior across energy, spin, and bonding metrics, and was selected as the most appropriate configuration for evaluating the interaction between $[Fe^{IV}O]^{2+}$ and Cys46 in the MS2 coat protein. The added CPCM analysis supports the robustness of this conclusion under an aqueous dielectric.

A basis-set convergence check was also performed by repeating one representative single-point calculation with the larger quadruple-ζ def2-QZVP basis (Table S10). The QZVP result reproduced the TZVP quintet's $\langle S^2 \rangle$ value and yielded only negligible changes in Löwdin spin populations on Fe, the terminal oxo, and the Cys46 sulfur (≤0.02 e^- or spin units). The frontier

orbital gaps were likewise unchanged (3.62 vs 3.64 eV), and the SCF energy was lower by 0.02 Eh (~12.6 kcal·mol^{-1}) relative to TZVP, consistent with variational principles. These checks confirmed that energetic, electronic, and bonding trends are already well converged at the CPCM/PBE0-D3(BJ)/def2-TZVP level used for reporting mechanistic insights.

Table S10. Comparison of solvated quintet ferryl–Cys46 cluster results obtained from single-point calculations at the PBE0-D3(BJ) level with def2-TZVP and def2-QZVP basis sets (with CPCM).

Property	TZVP	QZVP	Comment
Final SCF energy (E; Eh)	–1984.48	–1984.50	QZVP has lower energy than
ΔE (kcal/mol; relative to QZVP)	12.6	0.0	TZVP, and hence more stable
Spin contamination ⟨S²⟩ (dev.)	6.104	6.103	Identical spin purity
Fe Löwdin spin	2.752	2.750	Negligible difference
O(ferryl) Löwdin spin	0.197	0.194	Negligible difference
S (Cys46) Löwdin spin	0.040	0.039	Negligible difference
Mayer Fe=O bond order	1.75	1.78	Same bonding
Mayer Fe–S bond order	0.68	0.50	Lower, but trend unchanged
Mayer Fe–O(carbonyl)	0.39	0.37	Negligible difference
Frontier orbital gap (eV)	3.62	3.64	Negligible difference

S11c. QM-based electronic clues to protein oxidation in electrocoagulated samples

The QM cluster model offered useful insights into the electronic structure and bonding characteristics of the Fe(IV)=O–Cys46 interaction. Among the spin states evaluated, the quintet configuration exhibited features consistent with oxidative activity, including Fe–S bond formation, partial charge transfer to the sulfur atom, and radical character on the ferryl oxygen, while the doublet and triplet were discounted due to severe spin contamination or solvent instability (SI Section S11b). These electronic signatures help rationally determine how high-valent iron could initiate oxidation of thiol groups in proteins.

The solvent-phase (CPCM, water) single-point analysis preserved these qualitative features, and further highlighted the quintet as the only state with consistently clean spin and chemically reasonable bonding, even though the absolute HOMO–LUMO gaps shifted; the quintet still presents the most chemically consistent description of a reactive ferryl–thiolate. A spin density of

~0.8 was also observed on the cysteine backbone carbon, reflecting delocalization through the Fe–S–Cys bonding framework and not a true radical site (SI Table S9).

Although simplified, the model helped contextualize the MALDI-TOF results, particularly the +16, +32, and +48 Da mass shifts observed after electrochemical treatment. The MS2 capsid is composed of a single copy of the maturation protein and 178 coat proteins arranged as 89 dimers, forming a T = 3 quasi-icosahedral structure.[57, 107] Each coat-protein subunit contains two cysteine residues, Cys46 and Cys101.[57] Molecular modeling and spatial interaction mapping indicated that ferryl ion lay close to Cys46 (closer than 5.6Å, Figure 3A of the main manuscript), making it the more likely oxidation site. Consequently, it can be presumed that oxidation preferentially occurred primarily at Cys46 rather than uniformly across all residues.

These mass changes are consistent with a plausible stepwise oxidation of cysteine to sulfenic, sulfinic, and sulfonic acid forms. The QM analysis supported the plausibility of such transformations by showing that Cys46 was electronically well-positioned for oxidative attack by Fe(IV)=O. The docking results identified Cys46 as the most likely target residue, and the QM results reinforce that interpretation from a chemical bonding perspective.

It is important to note that the calculations used static cluster models: geometries were optimized in the gas phase, and solvation effects were included only at the single-point energy stage via CPCM (water; ε = 78.4), and did not account for protein dynamics or cooperative interactions with surrounding residues. Only ground-state geometries were considered; no transition states or energy barriers were computed. Other residues, such as Thr45, Trp 32 or Arg 49, were excluded from the QM region based on spatial separation in the docking model. While CPCM was included to emulate bulk water, it is a continuum model and does not capture explicit hydrogen bonding or specific ion/pH effects; pH was represented only through the chosen

protonation state (identical at pH 6.5 and 5.5 in our tests). Therefore, the analysis should be viewed as a snapshot of a plausible reactive configuration rather than a full mechanistic pathway.

Despite these limitations, the QM approach contributed a meaningful layer of mechanistic support. It offered atomic-level evidence for covalent and polar interactions that may underlie oxidative modifications observed experimentally, helping bridge docking predictions, quantum analysis, and mass spectrometric findings. The additional CPCM checks strengthen confidence that these conclusions extend to an aqueous dielectric environment relevant to the experiments, further supported by a def2-QZVP basis set check showing negligible changes (SI Section S11b).

Section S12. Secondary structure changes and oxidative damages

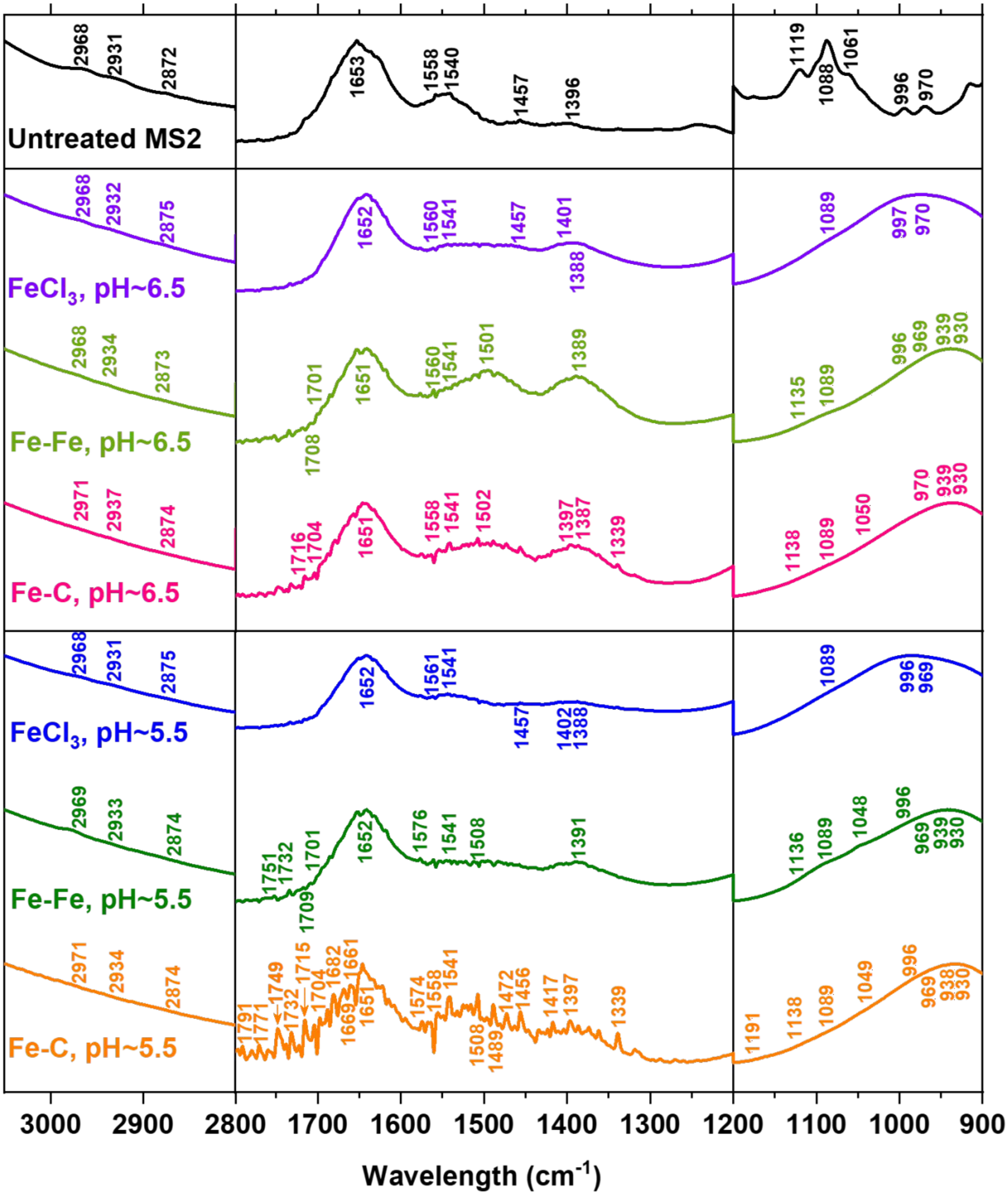

Figure S16. FTIR spectra of untreated, Fe-Fe electrocoagulated (iron anode and iron cathode), Fe-C electrocoagulated (iron anode and carbon cathode) at pH 6.5 and 5.5, 60 min, 20 mg/L total iron dosage. Spectra were discontinued at 2800 and 1200 cm^{-1} because peaks were normalized for different regions (3050-2800 cm^{-1}, 2800-1200 cm^{-1}, and 1200-900 cm^{-1}) were normalized to clearly see all less intense peaks across the complete ATR-FTIR spectra (3050-900 cm^{-1}). Spectra represent the average from three independent runs.

Table S11 compares amide II and carbonyl content across untreated and (electro)coagulated samples. Amide II content progressively decreased from 33% in untreated MS2 to as low as 1.7% in Fe–C EC at pH 5.5, indicating substantial loss of native secondary structure under more oxidative treatments. Concurrently, carbonyl content—undetectable in untreated MS2—increased markedly, reaching 4.2% in Fe–C EC at pH 5.5, consistent with extensive protein oxidation. These trends suggest that electrocoagulation, particularly with Fe–C at acidic pH, induced the most pronounced structural degradation and oxidative modification of the capsid protein. As expected, Fe-Fe electrocoagulation gave similar trends with smaller magnitudes. In case of Fe-Fe electrocoagulated MS2, aliphatic ketone (~3.2%), aliphatic aldehyde (0.24%), and anhydides (0.36%) were present at pH ~ 6.5, whereas aliphatic ketone (~4.9%), aliphatic aldehyde (0.36%), and anhydides (~0.43%) were present at pH ~ 5.5, but acyl chloride was not detected.

Table S11. Relative change in amide II and carbonyl peak areas untreated and (electro)coagulated MS2 samples.

**Sample**	**Amide II (%)**	**Carbonyl content (%)**
	**(1700-1600 cm^{-1})**	**(1820-1700 cm^{-1})**
Untreated MS2	33.0	0.0
$FeCl_3$, pH 6.5	28.1	0.1
$FeCl_3$, pH 5.5	23.2	0.1
Fe-Fe, pH 6.5	18.0	1.4
Fe-Fe, pH 5.5	12.3	3.2
Fe-C, pH 6.5	7.2	3.0
Fe-C, pH 5.5	1.7	4.2

Beyond these backbone and carbonyl changes, electrocoagulated samples also exhibited new peaks diagnostic of sulfur oxidation. After subtracting a same-pH reference spectrum (average of untreated and $FeCl_3$-coagulated viruses) to minimize pH and film-thickness effects, the EC–reference spectra revealed distinct bands at 1191, 1138, 1136, 1135, 1049, and 1048 cm^{-1} (SI Figure S16). These features, absent in the references and above local noise, corresponded to

sulfur–oxygen stretching [ν(S–O_x)] and are consistent with cysteine (possibly Cys46) residue oxidation.

Table S12. Assignment of protein secondary structure and carbonyl-associated groups[36, 58, 108-111]

**Wavenumber (cm^{-1})**	**Assignment**
**I. Secondary structure[108]**	
1692-1679	High component β-sheet
1667-1677	Turns
1661-1645	alpha helix
1644-1642	Unordered structure
1640-1624	Low component β-sheet
1622-1609	Aggregated structure
**II. Carbonyl-associated group**	
1704-1717	Aliphatic ketone
1731-1744	Aliphatic aldehyde
1749, 1758, 1759, 1810	Anhydride
1770, 1791	Acyl chloride

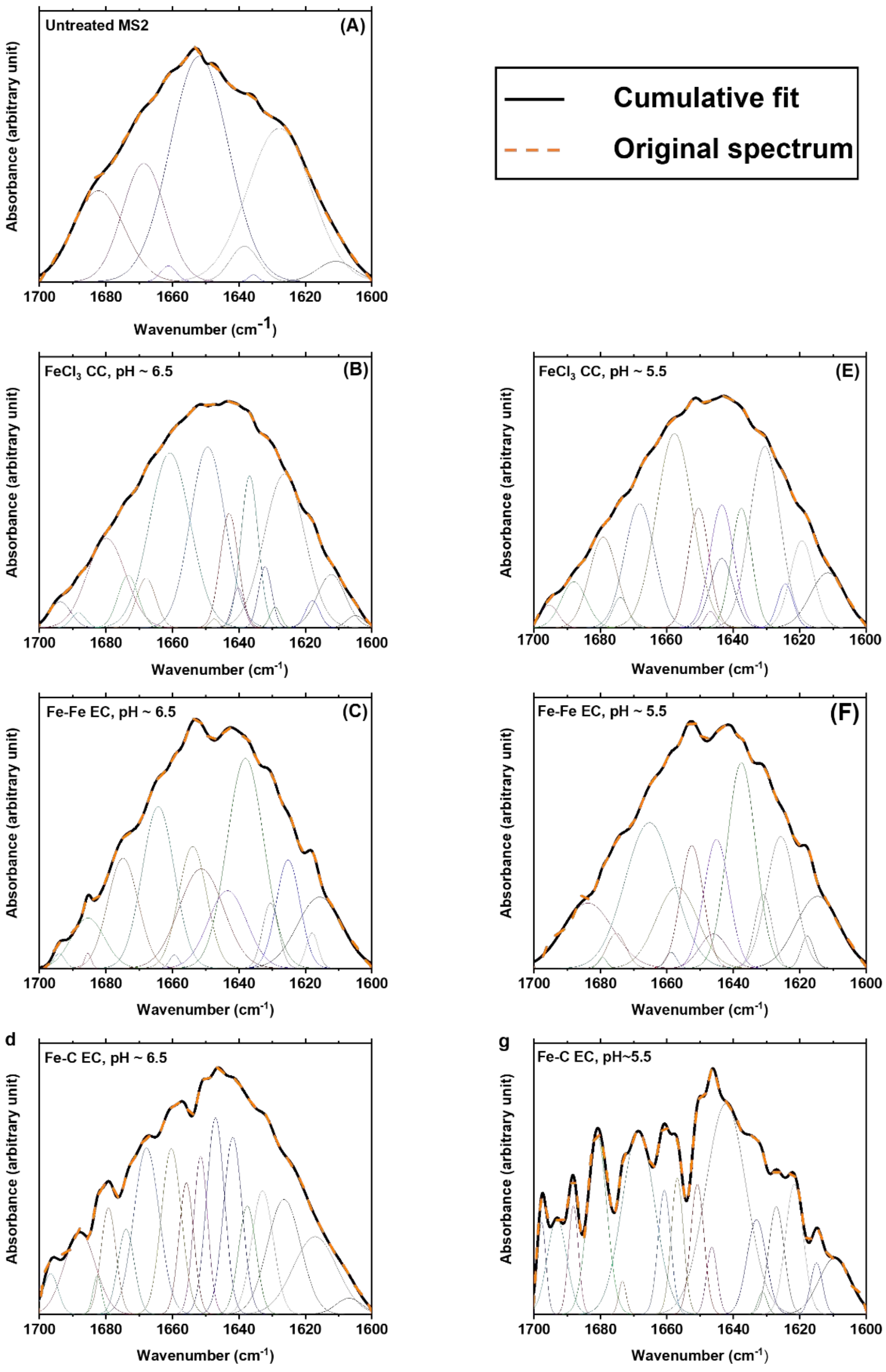

Figure S17. Decomposition of amide I region (1700-1600 cm^{-1}) for (A) untreated, (B, C) chemical coagulated (CC), (D, E) iron-iron electrocoagulated (Fe-Fe EC), and (F, G) iron-carbon electrocoagulated (Fe-C EC) MS2 at pH ~ 6.5 and 5.5. Average spectra from three independent runs were deconvoluted.

Table S13. Assignment and relative distribution of secondary structure of protein and carbonyl-associated assignments. WN – wavenumber, RA – relative area, $FeCl_3$ – $FeCl_3$ coagulation, Fe-Fe – iron-iron electrocoagulation, Fe-C – iron-carbon electrocoagulation

**Protein structure/ carbonyl groups**	**Untreated MS2**		**pH ~ 6.5**						**pH ~ 5.5**					
			**$FeCl_3$**		**Fe-Fe**		**Fe-C**		**$FeCl_3$**		**Fe-Fe**		**Fe-C**	
	**WN cm^{-1}**	**RA (%)**	**WN cm^{-1}**	**RA (%)**	**WN cm^{-1}**	**RA (%)**	**WN cm^{-1}**	**RA (%)**	**WN cm^{-1}**	**RA (%)**	**WN cm^{-1}**	**RA (%)**	**WN cm^{-1}**	**RA (%)**
**I. Assignment of secondary structure of protein and its relative area**														
Aggregated	1611<br>-	2.1	1612<br>1618<br>-	4.4<br>1.1	1616<br>1618<br>-	8.4<br>1.1	1617<br>-	9.1	1612<br>1619<br>-	1.4<br>0.9	1615<br>1618<br>-	8.9<br>1.1	1609<br>1615<br>1622	4.9<br>1.7<br>6.5
β-sheet (low component)	1628<br>1636<br>1638<br>-	28.1<br>0.2<br>2.8	1626<br>1629<br>1632<br>1637<br>1640	18.2<br>2.6<br>2.4<br>7.7<br>3.4	1625<br>1631<br>1638<br>-<br>-	6.9<br>2.9<br>20.8	1626<br>1633<br>1637<br>-	10.3<br>7.2<br>5.1	1624<br>1631<br>1638	8.7<br>10.9<br>5.3	1626<br>1632<br>1638<br>-	10.8<br>5.3<br>14.7	1627<br>1631<br>1633	4.7<br>0.5<br>5.1
β-sheet (high component)	1682<br>-	13.2	1680<br>1688<br>-	10.0<br>0.6	1685<br>1685<br>1694	4.4<br>4.3<br>0.4	1679<br>4.4<br>4.4	6.3<br>0.9<br>6.8	1679<br>1689<br>1695	14.9<br>4.5<br>1.0	1679<br>1683	0.4<br>7.5	1681<br>1688<br>-	9.6<br>4.0
Unordered					1643	7.6	1642	8.9	1643	2.9	1644	6.6	1642	23.8
α-helix	1652<br>1661<br>-	36.8<br>0.7	1647<br>1649<br>1661<br>-	0.2<br>16.4<br>20.9	1651<br>1654<br>1659<br>1664	7.3<br>9.5<br>0.3<br>13.8	1647<br>1652<br>1656<br>1660<br>-	7.4<br>6.0<br>5.2<br>9.0	1647<br>1650<br>1658<br>-	10.5<br>7.1<br>20.2	1647<br>1651<br>1655<br>1659<br>-	2.2<br>6.3<br>16.9<br>0.2	1646<br>1651<br>1657<br>1661<br>-	2.0<br>3.9<br>4.6<br>4.4
Turns	1669	14.5	1668<br>1673	5.8<br>6.1	1675<br>-	11.9	1668<br>1674	11.4<br>3.8	1668<br>1674	10.6<br>1.3	1666<br>1675	16.2<br>2.8	1669<br>1673	16.6<br>0.7
Unassigned/others		1.6		0.24		0.33		2.6		0.3		0.3		7.2
**II. Assignment of carbonyl-associated group and its relative area**														
Aliphatic ketone	-		-		1717<br>-	0.79	1705<br>-	1.43	-		1709<br>1711	0.2<br>2.23	1711	0.96
Aliphatic aldehyde	-		1744<br>-	0.1	1734	0.24	3.2<br>-	0.9	1733	0.2	1734<br>-	0.36	1731<br>1744	0.9<br>1.2
Anhydride	-				1759	0.36	1749	0.66	-		1758	0.43	1749	0.7
Acyl chloride	-		-		-		-		-		-		1791<br>1770	0.2<br>0.2

Figure S18 shows a clear inverse relationship: as the α-helix/β-sheet (α/β) ratio goes down, MS2 log reduction goes up ($r = -0.84$; $R^2 = 0.70$). Each triangle is one condition; the dashed lines are the 95% confidence bands. This matches the main-text results: a lower α/β also tracks with more carbonyls (Fig. 4D), greater inactivation (Fig. 4E), and more MALDI-detected damage (Fig. 4F). Together, these agree that loss of secondary structure is linked to oxidative damage and loss of function during electrocoagulation.

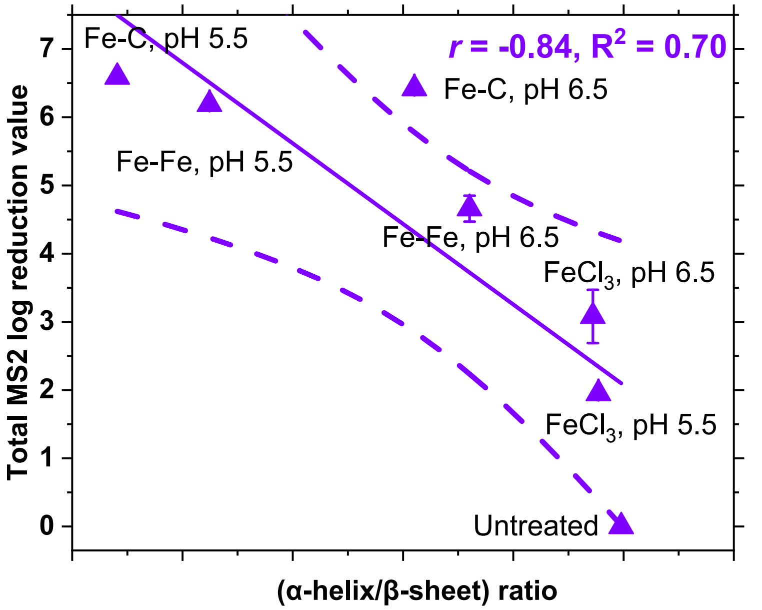

Figure S18. Association of α/β ratio with total MS2 log reduction value. The coefficient of determination (R^2) for each linear fit exceeds 0.7. The dashed lines represent the 95% confidence bands. Error bar represent the average ± standard deviation of relative peak intensities obtained after deconvolution.

Section S13. Evidence of Fenton reaction

S13a. Variation of total iron and iron (II)

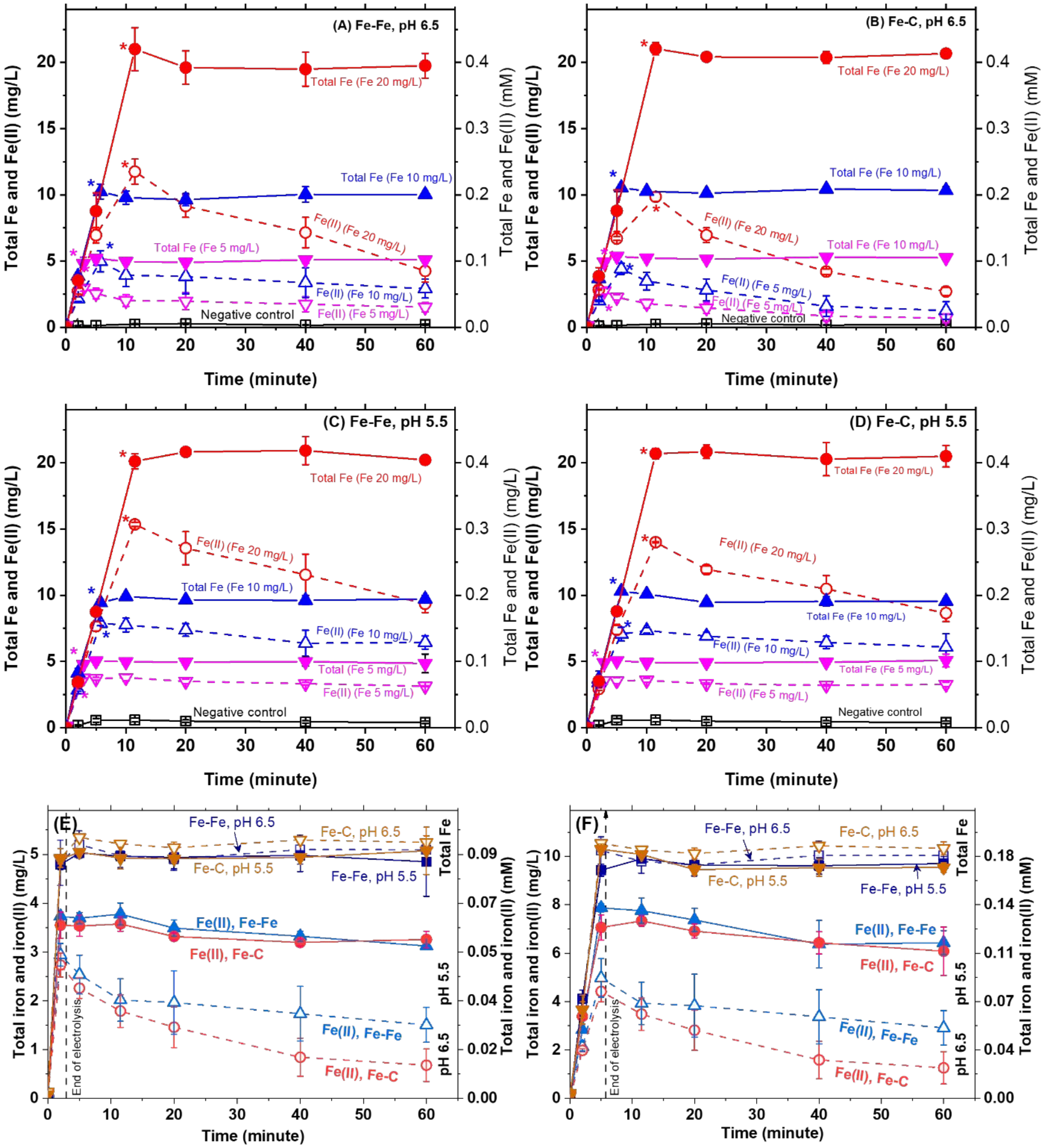

Figure S19. Temporal variation of total iron and iron(II) concentrations for Fe-Fe and Fe-C electrode systems for different iron dosages (5, 10, and 20 mg/L) at pH 6.5 (A, B) and pH 5.5 (C, D). For all conditions, asterisk (*) denotes end of electrolysis. Comparison of total iron and iron(II) at different condition (electrode configuration and pH) for 5 mg/L (E) and 10 mg/L (F) of iron dosages, where dashed line represents end of electrolysis. Data represent average ± standard deviation from three independent experiments.

S13b. Faradaic efficiency of hydrogen peroxide in electrocoagulation systems

For Fe-Fe and Fe-C electrocoagulation systems, theoretical doses of H_2O_2 were calculated using equation S20.

$$Faradaic\ efficiency\ (\%) = \frac{Measured\ concentration}{Theoretical\ dose} \times 100 \quad (S20)$$

where, theoretical H_2O_2 was estimated using equation S20 and measured concentrations of total H_2O_2 was determined per details mentioned in SI Section S5b

$$Theoretical\ dose\ \left(\frac{mg}{L}\right) = \frac{1000A_wIt}{ZFV} \quad (S21)$$

where, A_w represents molecular mass of H_2O_2 34 g/mol, I represents current (0.05 A), t is time (691 s for 20 mg/L of Fe dosage, 346 s for 10 mg/L of Fe dosage, and 173 s for 5 mg/L of Fe dosage), F is Faraday constant (96,485 C/mol), Z represents number of electrons transferred (= 2) V is volume (0.5 L).

H_2O_2 Faradaic efficiency (Table S13; Figure S20) generally decreased with increasing total iron dosage in both Fe–Fe and Fe–C electrocoagulation systems, with the specific exception of Fe–Fe at pH 6.5, where the Faradaic efficiency at 10 mg L^{-1} (23.0 %) was higher than at 5 mg L^{-1} (16.5 %). This trend reflected the balance between cathodic H_2O_2 generation and its rapid *in situ* consumption through iron-mediated oxidation pathways. At pH 5.5 and 6.5, the dominant sink for H_2O_2 is the high-valent ferryl species $[Fe^{IV}O]^{2+}$ formed from the reaction of Fe^{2+} and H_2O_2. Hydroxyl radicals may also form via Fenton-type reactions, but their contribution is expected to be minor under these mildly acidic conditions.[4, 20, 30, 36, 72-74, 82] At lower iron dosages, the dissolved Fe^{2+} concentration is smaller, slowing ferryl formation and allowing a higher fraction of cathodically produced H_2O_2 to persist in solution, which results in higher Faradaic efficiency. However, a lower iron dosage also corresponds to less total current passed—and thus less oxygen

reduction at the cathode—so the absolute H_2O_2 generated is also smaller. In the case of Fe–Fe at pH 6.5, the low production rate at 5 mg L^{-1} may have made the early stages of electrolysis more susceptible to cathodic pathways that do not yield H_2O_2, such as the four-electron oxygen reduction to water, explaining why 10 mg L^{-1} showed a higher Faradaic efficiency before ferryl-driven consumption became dominant at still higher iron dosage.

Table S14. Faradaic efficiency (FE) of H_2O_2 in different electrocoagulation system

Electrode configuration	Iron dose (mg/L)	Faradaic efficiency (%)	
		pH 5.5	pH 6.5
Fe-Fe	5	29.5	16.5
	10	23.1	23.0
	20	19.9	13.2
Fe-C	5	30.9	23.0
	10	31.3	25.1
	20	22.8	20.3

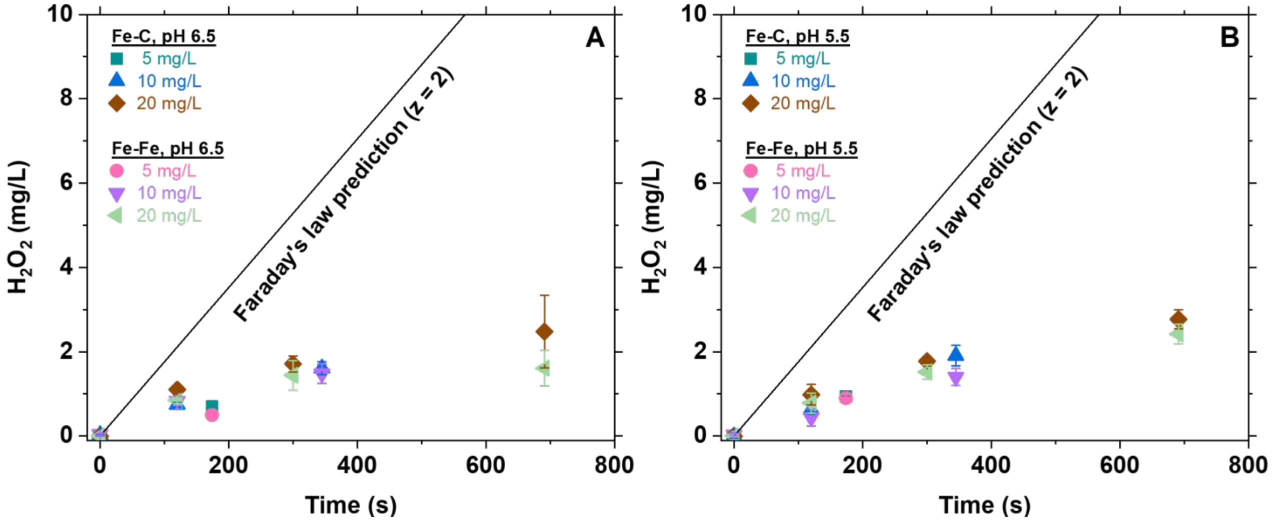

Figure S20. Faradaic efficiency of H_2O_2 production in Fe-Fe and Fe-C electrode systems at pH 6.5 and 5.5. Data represent average ± standard deviation from three independent experiments.

Differences between the two electrode configurations arose from cathode selectivity rather than iron release rate, as in both systems only the iron anode supplies Fe^{2+}. In the Fe–C configuration, the carbon cathode favors the two-electron oxygen reduction reaction to H_2O_2 and suppressed its electrochemical reduction[12, 13] (Section S3), enabling a greater fraction of the charge

to be retained as H_2O_2 at comparable iron dosages. In contrast, the iron cathode in the Fe–Fe configuration more readily facilitated cathodic H_2O_2 reduction and the four-electron oxygen reduction to water, lowering the fraction of charge captured as H_2O_2 and generally reducing Faradaic efficiency.

It is emphasized that these Faradaic efficiencies are conservative estimates because of *in situ* peroxide consumption by Fe(II) and decay during measurement given the associated fast kinetics.[112-114] Other cathodic processes such as H_2(g) evolution and minor side reactions[20, 28] (SI Section S4b) were not considered in the Faradaic efficiency estimates, as they were not measured and fell outside the scope of this study.

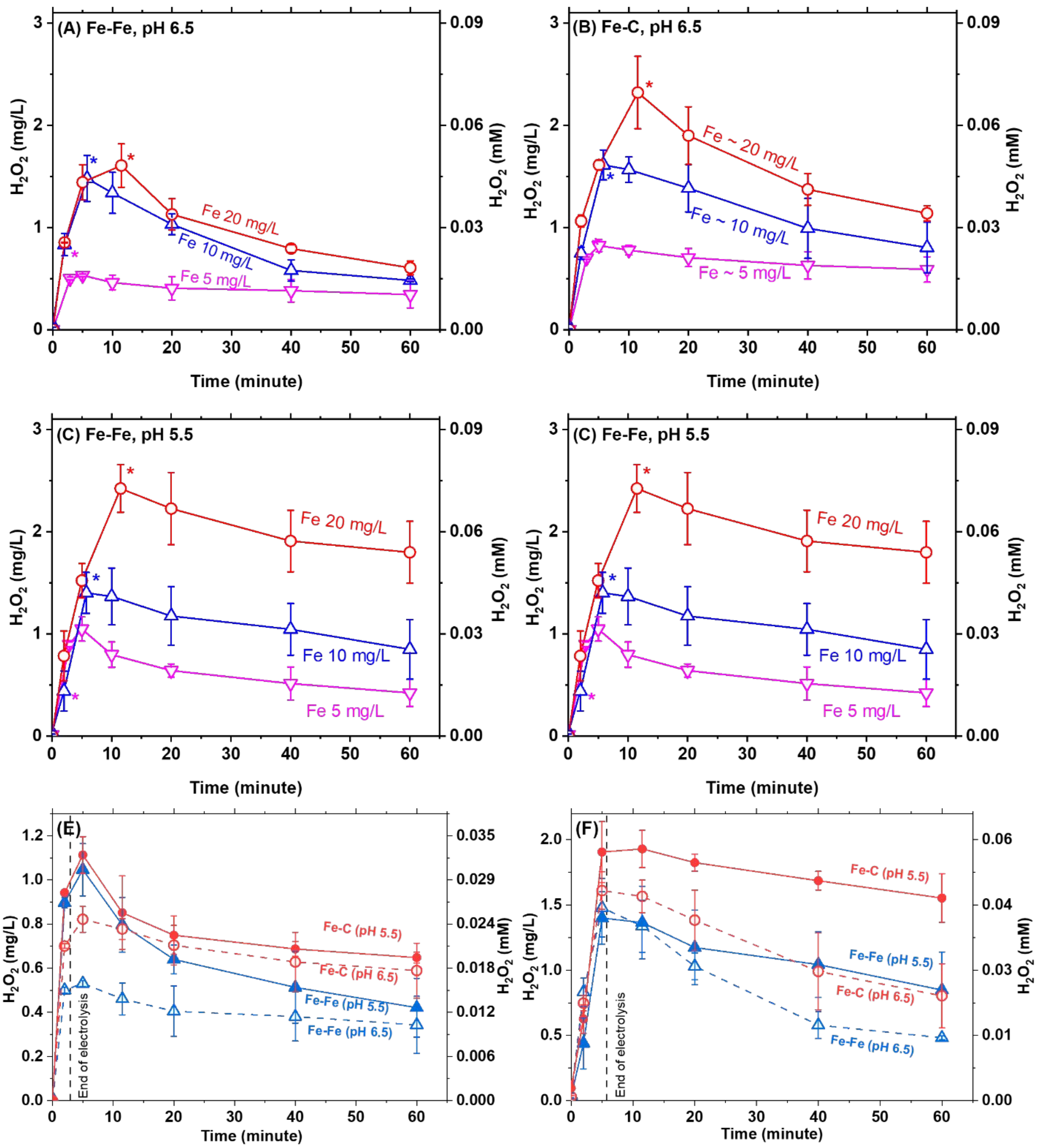

Figure S21. Temporal variation of total hydrogen peroxide concentrations for Fe-Fe and Fe-C electrode systems for different iron dosages (5, 10, and 20 mg/L) at pH 6.5 (A, B) and pH 5.5 (C, D). For all conditions, asterisk (*) denotes end of electrolysis. Comparison of hydrogen peroxide at different condition (electrode configuration and pH) for 5 mg/L (E) and 10 mg/L (F) of iron dosages, where dashed line represents end of electrolysis. For figures E and F, open and closed symbols correspond to experiments at pH 6.5 and 5.5, respectively. Data represent average ± standard deviation from three independent experiments.

Section S14. Control experiments for explaining virus inactivation

S14a. Virus time series profiles in the presence of hydrogen peroxide only

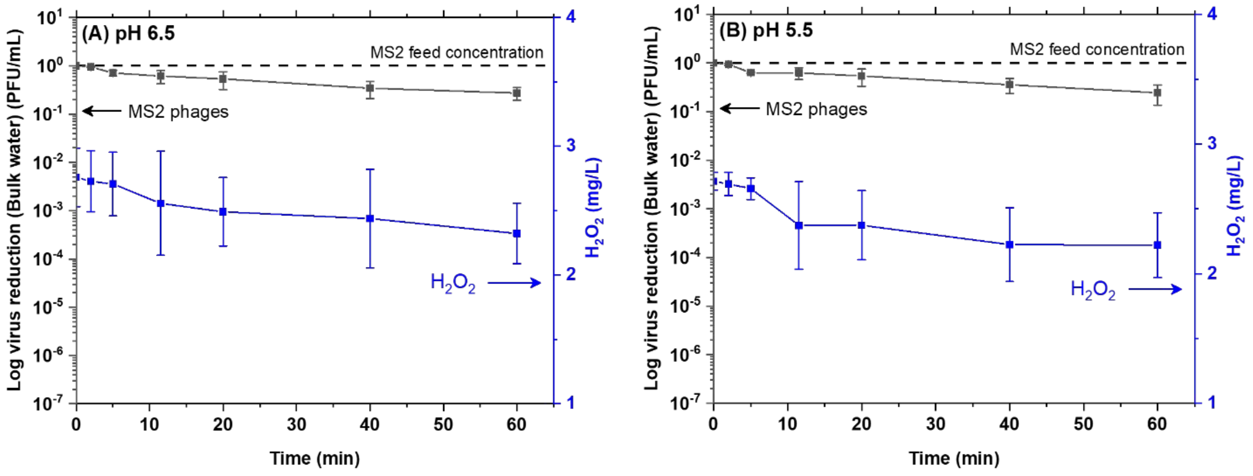

Figure S22. Effect of H_2O_2 on MS2 inactivation at pH (A) 6.5 and (B) pH 5.5. Data represent average ± standard deviation from three independent experiments.

We monitored changes in infective virus concentrations in the presence of only H_2O_2 (~2.75 mg/L) at both pH 6.5 and 5.5 in the synthetic secondary effluent containing Na_2SiO_3, NaCl, Na_2SO_4, $NaHCO_3$, $CaCl_2$, $MgCl_2$ (Table S1). Figure S22 shows that hydrogen peroxide alone had an insignificant effect on MS2 inactivation at both pH 6.5 and 5.5. The initial H_2O_2 concentration was 2.75 mg/L, and no appreciable viral reduction was observed over time at either pH. H_2O_2 levels decreased slightly, reaching approximately ~2.2 mg/L by the 60-minute mark at both pH levels. These results are consistent with previous publications that examined virus LRVs in simpler water matrices only containing $NaHCO_3$ and $CaCl_2$,[4, 20, 115-118] all showing that H_2O_2 alone negligibly inactivated MS2.

S14b. Systematic comparison of virus performance in the presence of iron salts ($FeCl_2$ and $FeCl_3$) and iron electrocoagulation (Fe-Fe and Fe-C)

Figure S22 compares MS2 reduction and inactivation by $FeCl_2$, $FeCl_3$, and electrocoagulation (Fe–Fe and Fe–C) at pH 6.5 and 5.5, with Figures S22A and S22C showing virus reduction from bulk water, and Figures S22B and S22D showing inactivation from suspension. As expected, $FeCl_3$ showed minimal MS2 inactivation across both pH conditions (Figures S22B and S22D), though some virus reduction was observed (Figures S22A and S22C) due to sweep coagulation—removal of viruses via enmeshment in iron(III) flocs.[4, 36, 92, 119-121] Note that $FeCl_3$ lacks redox activity and does not generate reactive oxygen species (ROS), explaining the absence of virucidal effects.

$FeCl_2$, in contrast, led to moderate MS2 inactivation, particularly at pH 5.5 (Figure S22D). This is attributed to two parallel mechanisms: (i) sweep coagulation as Fe^{2+} oxidizes to Fe^{3+} and (ii) partial ROS generation via the autoxidation of Fe^{2+} in the presence of dissolved oxygen.[4, 36] While this can produce hydroxyl radicals or other intermediates, it occurs slowly and without regeneration of Fe^{2+}.

Electrocoagulation systems (Fe–Fe and Fe–C) exhibited the highest MS2 inactivation at both pH levels (Figures S22B and S22D), with Fe–C consistently outperforming Fe–Fe. This superior performance of electrocoagulation was attributed to the synergistic action of two processes—(a) virus enmeshment in iron colloids and (B) virus inactivation due to generation of virucidal species as a part of Fenton chemistry.[4, 36] The *in situ* combination of Fe^{2+} and H_2O_2 drives Fenton chemistry, producing potent oxidants. Under the near-neutral pH conditions used here (6.5 and 5.5), $[Fe^{IV}O]^{2+}$ rather than free hydroxyl radicals is likely the dominant reactive species.[30, 72-74]

The effect of pH is evident in all treatments: MS2 inactivation was more pronounced at pH 5.5 (Figures S22C and S22D), consistent with enhanced ROS formation and faster iron redox cycling under mildly acidic conditions. The Fe–C system demonstrated the highest overall efficiency, due to more effective H_2O_2 generation and enhanced electron transfer at the carbon cathode,[12, 13] reinforcing it as the most potent configuration for virus inactivation among the systems tested.

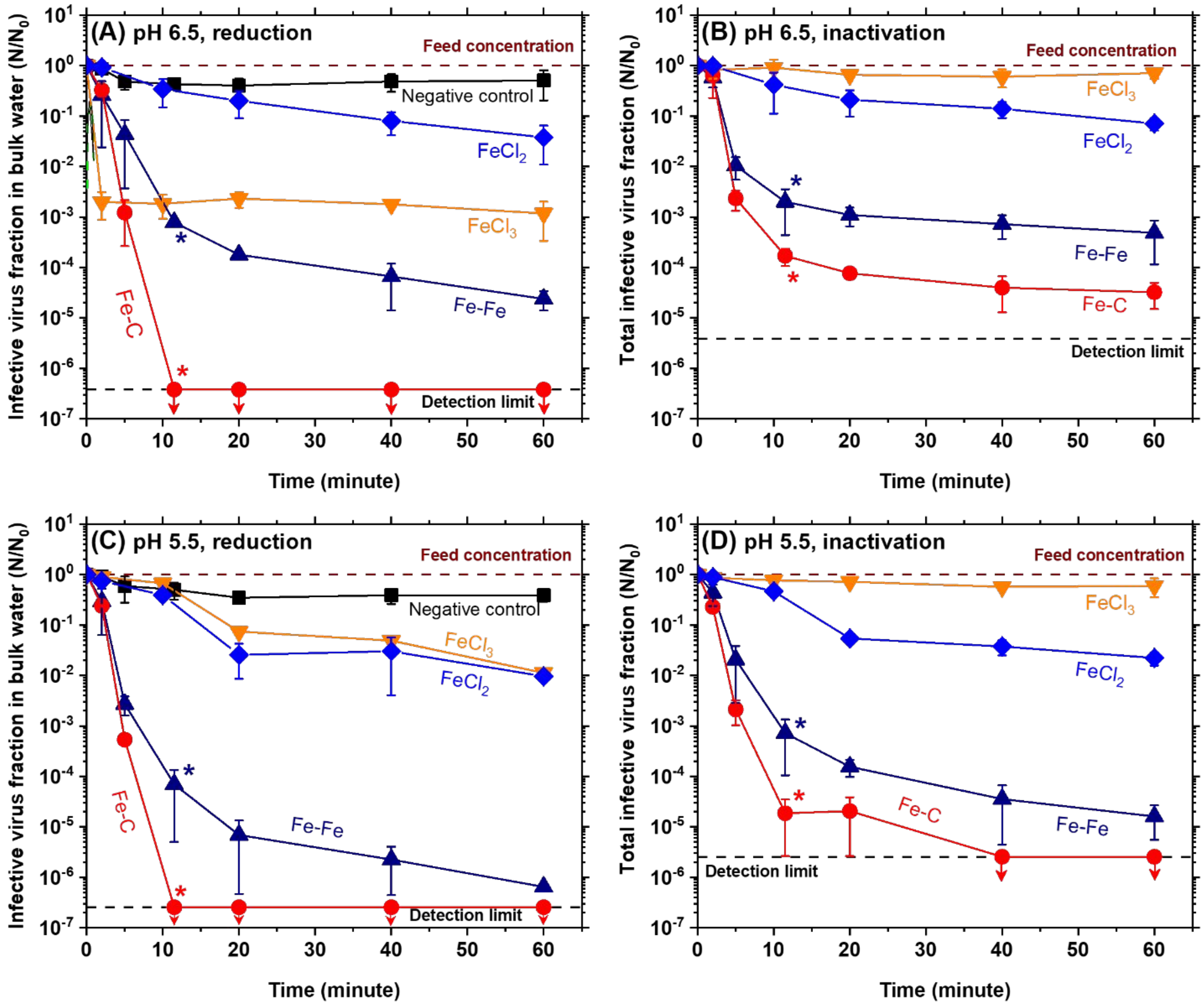

Figure S23. Comparison of MS2 reduction and inactivation by $FeCl_2$, $FeCl_3$, and electrocoagulation (Fe-Fe and Fe-C) at pH 6.5 and 5.5. MS2 reduction and inactivation were determined from bulk water and suspension (bulk water + flocs), respectively. For electrocoagulation systems, electrolysis was performed at 1 mA/cm^2 for 11.5 minutes as indicated by asterisk (*) mark with total Fe dosage of 20 mg/L. Data represent average ± standard deviation from three independent experiments.

Section S15. Organic matter and water-matrix effects on ROS and virus inactivation

S15a. Effect of organic matter on ROS during virus inactivation

Comparative studies have consistently shown that organic matter suppresses virus inactivation in electrochemical and oxidant-based systems because it consumes and transforms reactive oxygen species (ROS) before these short-lived intermediates could reach capsids and/or genomes. Mechanistically, this is largely due to organics (i) acting as a sink for short-lived oxidative species, (ii) transforming reactive oxygen species (ROS) into less reactive organic-centered radicals (e.g., phenoxyl-type intermediates), (iii) sequestering Fe(II), an electroFenton precursor, or (iv) fouling electrodes and reducing production of Fe(II) and H_2O_2, and (v) associating with viruses and changes their surface properties and reduces oxidant access. For instance, natural organic matter and turbidity reduced virus removal during electrooxidation following iron electrocoagulation, confirming competition for short-lived oxidants.[19] Consistent with this trend, lower MS2 inactivation was measured in secondary effluent than in buffered clean water when ozone, peroxymonosulfate, or hydrogen peroxide were applied, reflecting the scavenging capacity of effluent organic matter.[115] Virus disinfection experiments in natural water also reported lower LRVs in the presence of organic matter than in organic-free laboratory water, supporting the general observation that organic constituents suppress virus inactivation.[122] Thus, in matrices containing organic matter such as natural water and secondary or tertiary effluents, virus LRVs can be expected to diminish compared to those measured with synthetic waters without organics under otherwise identical chemical and electrochemical conditions. Contrastingly, in sunlight-photosensitized systems, natural organic matter (NOM) can sometimes increase MS2 inactivation through localized formation of singlet oxygen ($^1O_2$) within NOM microdomains.[123] This opposite

trend highlights that the effect of organics depends on the type of oxidants and organic matter present.

Mechanistically, organic matter competes directly with viruses for ROS, thereby lowering the fraction of short-lived oxidative species that can react with capsids and/or genomes leading to inactivation. The effective second-order rate constants for reactions between organic matter and $^{\bullet}OH$ are typically on the order of 10^8–10^9 M^{-1} s^{-1},[124-126] sufficient to deplete their pseudo equilibrium concentrations and yield lower apparent LRVs under otherwise identical conditions. In addition, reactions between ROS and organic matter can generate organic-centered radicals (for example, phenoxyl-type intermediates) that are far less reactive toward biological macromolecules, further diverting oxidative capacity away from oxidizing viral components.[127] Organic ligands can also complex dissolved Fe(II) and Fe(III), altering iron-redox turnover and decreasing the yield or lifetime of strongly oxidizing reactive species. [128-130] Collectively, these effects suppress the extent of virus inactivation by consuming or transforming ROS.

A separate issue is the possible fouling of electrode surfaces due to adsorption of organic matter during electrocoagulation. Natural and effluent organic matter can accumulate on surfaces of iron electrodes and form insulating layers that block electroactive sites, increase power consumption, and suppress anodic dissolution and cathodic reactions.[131, 132] Such surface fouling suppresses anodic dissolution and lowers the rate of Fe(II) generation, and can also reduce cathodic activity, thereby diminishing H_2O_2 production under aerobic conditions.[133] Persistent accumulation of organic (and inorganic) species on iron electrodes has been reported during long-term operation of electrocoagulation systems treating natural waters and groundwaters, resulting in electrode passivation and reduced treatment efficiency.[134, 135] In matrices such as secondary or tertiary

wastewater effluents, these electrode-fouling processes are expected to act in parallel to ROS scavenging by organic matter to reduce overall electrocoagulation performance and virus LRVs.

Another mechanism by which organic matter influences virus LRVs is by adsorbing to them.[136, 137] This phenomenon forms a physical layer in the proximity of sorption sites thereby limiting the penetration of oxidants and their ability to reach locations of potentially reactive amino acids on the virus capsid. Organic coatings can also change local surface charge and hydration, which reduces contact between oxidants and the viral proteins.[136, 137] Indeed, laboratory studies have empirically shown that waterborne viruses associated with organic matter and/or suspended debris required higher disinfectant exposures and were inactivated more slowly.[136] Such organic matter–virus associations therefore add an additional barrier that suppresses virus LRVs in engineered systems.

S15b. Water-quality considerations influencing virus inactivation

Virus inactivation during electrocoagulation can be expected to depend on the water composition. In a simple water matrix such as phosphate buffered saline solution or synthetic water containing only bicarbonate alkalinity, there are very few species that can interact with iron and/or ROS.[4, 21, 24, 26, 36] Under these conditions, ROS should persist longer and LRVs would be highest.[24, 36]

In the current study, the feedwater composition was tailored towards secondary wastewater effluents (but without organics). Our synthetic water contained higher levels of hardness and bicarbonate alkalinity and introduces species such as silica, sulfate, and chloride (Table S1), water chemistry becomes more complex. Hardness and alkalinity can influence local pH and iron hydrolysis.[91, 138] Silica and sulfate can interact with iron solids and can change the way flocs form.[139] Chloride can form chlorine-based radicals and other reactive chlorine species.[140] Sulfate

can form sulfate-based radicals or stabilize certain iron species.[141, 142] These processes can change which oxidants form and how long they remain active. Virus inactivation in the synthetic secondary wastewater effluent used by us is therefore expected to be lower than in simple laboratory water.

Actual secondary wastewater effluents also contain organic matter, which will react with oxidants[128, 129] often transforming them into less reactive species.[127] It also binds iron,[125, 127] fouls electrode surfaces,[131-133] and attaches to virus particles[136, 137] (details in Section S15b). These processes are expected to further reduce oxidant availability. For these reasons, LRVs in real effluents are expected to be lower than in our synthetic effluent. Although organic matter was excluded in this study, these literature-supported effects assist in placing our results in the context of water quality conditions found in the real world.

Table S15. Summary of published studies reporting biochemical and biophysical modifications to MS2 bacteriophage capsid proteins under different disinfection treatments.

No.	Treatment	Conditions	Matrix	LRV	Mechanism	Affected residues	Reference
1	Iron electrocoagulation	Bench scale with iron anode and iron cathode (Fe-Fe) and iron anode and carbon cathode (Fe-C); 20 mg/L iron dose for 60 minutes	Synthetic secondary effluent (Table S1); pH 6.5 and 5.5	≳ 6.7 in Fe-C at both pH ~5 and ~6 at pH 6.5 and 5.5 in Fe-Fe, respectively	Non-proteolytic capsid modification by $[Fe^{IV}O]^{2+}$ (and less likely by $^{\bullet}OH$)	N-terminal region (Ser23-Cys46); Susceptible residues: Cys46, Arg49, and Trp32	**This study**
2	Free chlorine (HOCl)	Bench-scale free chlorine (3.1 mg/L as Cl_2), continuous quench-flow reactor, quantitative proteomics	Buffered phosphate solution (pH 7.5)	Up to ~3-log inactivation (mechanistic emphasis)	Peptide-level oxidation; SASA–decay correlation; peptides in host-attachment/genome-injection proteins most predictive of inactivation	Met (sulfoxide), Tyr (3-chlorotyrosine), Cys-containing peptides detected but quantification limited due to dithiothreitol	143
3	UV_{254} (collimated beam)	Bench scale with 254 nm and exposure up to ~8-log MS2 inactivation	Dilution buffer (DB): 5 mM phosphate, 10 mM NaCl, pH 7.4 (per SI)	Up to 8-log (study range)	Capsid protein oxidation (non-proteolytic) and backbone cleavage (proteolytic); RNA replication also impaired	Outer-surface Pro117, Tyr129 modified; interior Thr45–Cys46–Ser47 involved; peptide-level cleavage products detected	24
4	UV_{254} (genome-mediated cleavage)	254 nm; top-down MS (FT-ICR/Orbitrap/TOF)	Buffered lab water (see Methods)	Not reported (focus on mechanism)	Genome-mediated, site-specific capsid protein cleavage (proteolytic) (RNA-dependent)	Backbone cleavage at Cys46–Ser47 in the MS2 coat protein	22
5	Singlet oxygen ($^{1}O_2$; Rose Bengal sensitization)	Photosensitized $^{1}O_2$; exposures up to ~8-log MS2 inactivation	DB: 5 mM phosphate, 10 mM NaCl, pH 7.4	Up to 8-log (study range)	Selective oxidation of susceptible residues on capsid surface (non-proteolytic)	Oxidation detected in peptide 84–106; exterior Cys101; Met88 within 84–106 reported as hotspot	24
6	Free chlorine ($HOCl/OCl^{-}$)	Bench-scale chlorination; peptide-resolved MS	Buffered lab water (phosphate; see Methods)	Varied (mechanistic	Capsid protein damage with widespread peptide degradation;	Reacts broadly across peptides; targets Cys, Tyr,	24, 144

No.	Treatment	Conditions	Matrix	LRV	Mechanism	Affected residues	Reference
				study across multiple logs)	backbone cleavage also observed	Trp; peptide-level backbone cleavage detected in intact capsid protein	
7	Chlorine dioxide (ClO_2)	Bench-scale; peptide-resolved MS	Buffered lab water (phosphate; see Methods)	Varied (mechanistic study)	Selective oxidation of susceptible domains; interference with host-cell recognition/binding	Preferential degradation of peptides 84–106 and 59–82 (contains Trp); selectivity versus free chlorine	[24, 144]
8	Ferrate ($Fe(VI)O_4^{2-}$)	pH 6–11; 5–30°C; inhibition constant k_i = 2.27 L·mgFe⁻¹·min⁻¹ at 1.23 mgFe/L (pH 7, 25°C); ~4-log at Ct ≈ 4 mgFe·min·L⁻¹	Buffered lab water (phosphate/borate depending on pH)	≥4-log (conditions above)	Capsid protein damage localized to cysteine-containing regions; protein cleavage in one region (proteolytic); genome damage follows	Damage localized near Cys46 (peptide 44–49, VTCSVR) and near Cys101 (within 84–106 region); cleavage in Cys46 region	[26]
9	Free chlorine (HOCl) — expanded mapping	HOCl in buffer; proteomics mapping	Buffered lab water (see Methods)	0–3+ (varies by dose/time; mechanistic emphasis)	Stable oxidation and cross-linking of capsid proteins; CP–RNA cross-links	Stable oxidation (and chlorination) of Cys, Met, Tyr, Trp; evidence for CP–RNA and CP–CP crosslinking	[144]
10	UV_{254} — peptide domain susceptibility (comparative across leviviruses)	254 nm; peptide-resolved MS during incremental inactivation	Buffered lab water (see Methods)	Multiple-log series (mechanistic)	Capsid domain-specific damage; genome-mediated cleavage in MS2/fr but not GA	MS2 peptides 44–49 and 114–129 most affected; cleavage around Cys46–Ser47 requires RNA	[23]

References

1. Environmental Protection Agency, Drinking water contaminant candidate list 5-final; 87 FR 68060. In 2022. https://www.govinfo.gov/content/pkg/FR-2022-11-14/pdf/2022-23963.pdf

2. Amarasiri, M.; Kitajima, M.; Nguyen, T. H.; Okabe, S.; Sano, D., Bacteriophage removal efficiency as a validation and operational monitoring tool for virus reduction in wastewater reclamation: Review. *Water Research* **2017,** *121*, 258-269. DOI: https://doi.org/10.1016/j.watres.2017.05.035

3. Gerrity, D.; Crank, K.; Steinle-Darling, E.; Pecson, B. M., Establishing pathogen log reduction value targets for direct potable reuse in the United States. *AWWA Water Science* **2023,** *5*, (5), e1353. DOI: https://doi.org/10.1002/aws2.1353

4. Kim, K.; Sen, A.; Chellam, S., Removal and inactivation of nonenveloped and enveloped virus surrogates by conventional coagulation and electrocoagulation using aluminum and iron. *ACS ES&T Engineering* **2022,** *2*, (10), 1974-1986. DOI: http://doi.org/10.1021/acsestengg.2c00128

5. Pecson, B. M.; Martin, L. V.; Kohn, T., Quantitative PCR for determining the infectivity of bacteriophage MS2 upon inactivation by heat, UV-B radiation, and singlet oxygen: Advantages and limitations of an enzymatic treatment to reduce false-positive results. *Applied and Environmental Microbiology* **2009,** *75*, (17), 5544-5554. DOI: https://doi.org/10.1128/AEM.00425-09

6. APHA; AWWA; WEF *Standard methods for the examination of water and wastewater*; American Public Health Association, American Water Works Association, Water Environment Federation: Washington, D.C,, 2017; pp 9(1)-9(231), https://www.standardmethods.org/doi/epdf/10.2105/SMWW.2882.178

7. Abada, B.; Safarik, J.; Ishida, K. P.; Chellam, S., Elucidating foulant diversity during full-scale potable reuse: Forensic analysis of lead and lag elements of a three-stage reverse osmosis system. *ACS ES&T Engineering* **2022,** *2*, (11), 2116-2129. DOI: https://doi.org/10.1021/acsestengg.2c00171

8. Abada, B.; Joag, S.; Alspach, B.; Bustamante, A.; Chellam, S., Inorganic and organic silicon fouling of nanofiltration membranes during pilot-scale direct potable reuse. *ACS ES&T Engineering* **2023,** *3*, (9), 1413-1423. DOI: https://doi.org/10.1021/acsestengg.3c00172

9. Abada, B.; Safarik, J.; Ishida, K. P.; Chellam, S., Surface characterization of end-of-life reverse osmosis membranes from a full-scale advanced water reuse facility: Combined role of bioorganic materials and silicon on chemically irreversible fouling. *Journal of Membrane Science* **2022,** *653*, 120511. DOI: https://doi.org/10.1016/j.memsci.2022.120511

10. Kim, K.; Castillo, C.; Jang, G. G.; Zhang, Y.; Tsouris, C.; Chellam, S., Porous iron electrodes reduce energy consumption during electrocoagulation of a virus surrogate: Insights into performance enhancements using three-dimensional neutron computed tomography. *ACS ES&T Engineering* **2024,** *4*, (10), 2573-2584. DOI: http://doi.org/10.1021/acsestengg.4c00317

11. Pecson, B. M.; Kaufmann, A.; Gerrity, D.; Haas, C. N.; Seto, E.; Ashbolt, N. J.; Slifko, T.; Darby, E.; Olivieri, A., Science-based pathogen treatment requirements for direct potable reuse. *Environmental Science: Water Research & Technology* **2023,** *9*, (12), 3377-3390. DOI: https://doi.org/10.1039/D3EW00362K

12. Wang, N.; Ma, S.; Zuo, P.; Duan, J.; Hou, B., Recent progress of electrochemical production of hydrogen peroxide by two-electron oxygen reduction reaction. *Advanced Science* **2021,** *8*, (15), 2100076. DOI: https://doi.org/10.1002/advs.202100076

13. Kulkarni, A.; Siahrostami, S.; Patel, A.; Nørskov, J. K., Understanding catalytic activity trends in the oxygen reduction reaction. *Chemical Reviews* **2018,** *118*, (5), 2302-2312. DOI: https://doi.org/10.1021/acs.chemrev.7b00488

14. Bicudo, B.; van der Werff, B.-J.; Medema, G.; van Halem, D., Disinfection during iron electrocoagulation: Differentiating between inactivation and floc entrapment for *Escherichia coli* and somatic coliphage ØX174. *ACS ES&T Water* **2022,** *2*, (10), 1707-1714. DOI: https://doi.org/10.1021/acsestwater.2c00230

15. Tian, Y.; He, W.; Zhu, X.; Yang, W.; Ren, N.; Logan, B. E., Energy efficient electrocoagulation using an air-breathing cathode to remove nutrients from wastewater. *Chemical Engineering Journal* **2016,** *292*, 308-314. DOI: https://doi.org/10.1016/j.cej.2016.02.004

16. Ren, G.; Zhou, M.; Su, P.; Liang, L.; Yang, W.; Mousset, E., Highly energy-efficient removal of acrylonitrile by peroxi-coagulation with modified graphite felt cathode: Influence factors, possible mechanism. *Chemical Engineering Journal* **2018,** *343*, 467-476. DOI: https://doi.org/10.1016/j.cej.2018.02.115

17. Sinclair, R. G.; Rose, J. B.; Hashsham, S. A.; Gerba, C. P.; Haas, C. N., Criteria for selection of surrogates used to study the fate and control of pathogens in the environment. *Applied and Environmental Microbiology* **2012,** *78*, (6), 1969-1977. DOI: https://doi.org/10.1128/AEM.06582-11

18. Havelaar, A. H.; van Olphen, M.; Drost, Y. C., F-specific RNA bacteriophages are adequate model organisms for enteric viruses in fresh water. *Applied and Environmental Microbiology* **1993,** *59*, (9), 2956-2962. DOI: https://doi.org/10.1128/aem.59.9.2956-2962.1993

19. Heffron, J.; McDermid, B.; Maher, E.; McNamara, P. J.; Mayer, B. K., Mechanisms of virus mitigation and suitability of bacteriophages as surrogates in drinking water treatment by iron electrocoagulation. *Water Research* **2019,** *163*, 114877. DOI: http://doi.org/10.1016/j.watres.2019.114877

20. Kim, J. Y.; Lee, C.; Sedlak, D. L.; Yoon, J.; Nelson, K. L., Inactivation of MS2 coliphage by Fenton's reagent. *Water Research* **2010,** *44*, (8), 2647-2653. DOI: http://doi.org/10.1016/j.watres.2010.01.025

21. Wigginton, K. R.; Pecson, B. M.; Sigstam, T.; Bosshard, F.; Kohn, T., Virus inactivation mechanisms: Impact of disinfectants on virus function and structural integrity. *Environmental Science & Technology* **2012,** *46*, (21), 12069-12078. DOI: http://doi.org/10.1021/es3029473

22. Wigginton, K. R.; Menin, L.; Sigstam, T.; Gannon, G.; Cascella, M.; Hamidane, H. B.; Tsybin, Y. O.; Waridel, P.; Kohn, T., UV radiation induces genome-mediated, site-specific cleavage in viral proteins. *ChemBioChem* **2012,** *13*, (6), 837-845. DOI: https://doi.org/10.1002/cbic.201100601

23. Sigstam, T.; Gannon, G.; Cascella, M.; Pecson, B. M.; Wigginton, K. R.; Kohn, T., Subtle differences in virus composition affect disinfection kinetics and mechanisms. *Applied Environmental Microbiology* **2013,** *79*, (11), 3455-67. DOI: http://doi.org/10.1128/aem.00663-13

24. Wigginton, K. R.; Menin, L.; Montoya, J. P.; Kohn, T., Oxidation of virus proteins during UV_{254} and singlet oxygen mediated inactivation. *Environmental Science & Technology* **2010,** *44*, (14), 5437-5443. DOI: http://doi.org/10.1021/es100435a

25. Ye, Y.; Chang, P. H.; Hartert, J.; Wigginton, K. R., Reactivity of enveloped virus genome, proteins, and lipids with free chlorine and UV_{254}. *Environmental Science & Technology* **2018,** *52*, (14), 7698-7708. DOI: https://doi.org/10.1021/acs.est.8b00824

26. Hu, L.; Page, M. A.; Sigstam, T.; Kohn, T.; Mariñas, B. J.; Strathmann, T. J., Inactivation of bacteriophage MS2 with potassium ferrate(VI). *Environmental Science & Technology* **2012,** *46*, (21), 12079-12087. DOI: https://doi.org/10.1021/es3031962

27. Li, L.; van G., C. M.; Addy, S. E. A.; Yao, J.; Gao, N.; Gadgil, A. J., Modeling As(III) oxidation and removal with iron electrocoagulation in groundwater. *Environmental Science & Technology* **2012,** *46*, (21), 12038-12045. DOI: https://doi.org/10.1021/es302456b

28. Wiegand, H. L.; Orths, C. T.; Kerpen, K.; Lutze, H. V.; Schmidt, T. C., Investigation of the iron–peroxo complex in the Fenton reaction: Kinetic indication, decay kinetics, and hydroxyl radical yields. *Environmental Science & Technology* **2017,** *51*, (24), 14321-14329. DOI: https://doi.org/10.1021/acs.est.7b03706

29. Heffron, J.; Ryan, D. R.; Mayer, B. K., Sequential electrocoagulation-electrooxidation for virus mitigation in drinking water. *Water Research* **2019,** *160*, 435-444. DOI: http://doi.org/10.1016/j.watres.2019.05.078

30. Bataineh, H.; Pestovsky, O.; Bakac, A., pH-induced mechanistic changeover from hydroxyl radicals to iron(iv) in the Fenton reaction. *Chemical Science* **2012,** *3*, (5), 1594-1599. DOI: https://doi.org/10.1039/C2SC20099F

31. Keenan, C. R.; Sedlak, D. L., Factors affecting the yield of oxidants from the reaction of nanoparticulate zero-valent iron and oxygen. *Environmental Science & Technology* **2008,** *42*, (4), 1262-1267. DOI: https://doi.org/10.1021/es7025664

32. Duan, Y.; Jiang, W.; Sedlak, D. L., Surface pocesses control the fate of reactive oxidants generated by electrochemical activation of hydrogen peroxide on stainless-steel electrodes. *Environmental Science & Technology* **2023,** *57*, (47), 18680-18689. DOI: https://doi.org/10.1021/acs.est.2c08404

33. Deng, G.; Wang, Z.; Ma, J.; Jiang, J.; He, D.; Li, X.; Szczuka, A.; Zhang, Z., Ferryl ion in the photo-Fenton process at acidic pH: Occurrence, fate, and implications. *Environmental Science & Technology* **2023,** *57*, (47), 18586-18596. DOI: https://doi.org/10.1021/acs.est.2c06373

34. Xie, S.; Li, C.; Liao, P.; Wang, J.; Chen, J.; Qian, A.; Zhang, Y.; Wei, T.; Cheng, D.; Jia, M., Experimental and modeling evidence of hydroxyl radical production in iron electrocoagulation as a new mechanism for contaminant transformation in bicarbonate electrolyte. *Water Research* **2022,** *220*, 118662. DOI: https://doi.org/10.1016/j.watres.2022.118662

35. Chen, Y.; Miller, C. J.; Waite, T. D., pH dependence of hydroxyl radical, ferryl, and/or ferric peroxo species generation in the heterogeneous fenton process. *Environmental Science & Technology* **2022,** *56*, (2), 1278-1288. DOI: https://doi.org/10.1021/acs.est.1c05722

36. Kim, K.; Narayanan, J.; Sen, A.; Chellam, S., Virus removal and inactivation mechanisms during iron electrocoagulation: Capsid and genome damages and electro-Fenton reactions. *Environmental Science & Technology* **2021,** *55*, (19), 13198-13208. DOI: http://doi.org/10.1021/acs.est.0c04438

37. APHA; AWWA; WEF, Fe IRON, Method 3500. . In *Standard methods for the examination of water and wastewater*, APHA press: Washington DC, 2020; Vol. 3. https://www.standardmethods.org/doi/epdf/10.2105/SMWW.2882.055

38. Stookey, L., Ferrozine---a new spectrophotometric reagent for iron. *Analytical Chemistry* **1970,** *42*, (7), 779-781. DOI: https://doi.org/10.1021/ac60289a016

39. Florence, T. M.; Stauber, J. L.; Mann, K. J., The reaction of copper-2,9-dimethyl-1,10-phenanthroline with hydrogen peroxide. *Journal of Inorganic Biochemistry* **1985,** *24*, (4), 243-254. DOI: https://doi.org/10.1016/0162-0134(85)85053-4

40. Baga, A. N.; Johnson, G. R. A.; Nazhat, N. B.; Saadalla-Nazhat, R. A., A simple spectrophotometric determination of hydrogen peroxide at low concentrations in aqueous solution. *Analytica Chimica Acta* **1988,** *204*, 349-353. DOI: https://doi.org/10.1016/S0003-2670(00)86374-6

41. Picaud, V.; Giovannelli, J. F.; Truntzer, C.; Charrier, J. P.; Giremus, A.; Grangeat, P.; Mercier, C., Linear MALDI-ToF simultaneous spectrum deconvolution and baseline removal. *BMC Bioinformatics* **2018,** *19*, (1), 123. DOI: https://doi.org/10.1186/s12859-018-2116-3

42. Xu, G.; Stupak, J.; Yang, L.; Hu, L.; Guo, B.; Li, J., Deconvolution in mass spectrometry based proteomics. *Rapid Communications in Mass Spectrometry* **2018,** *32*, (10), 763-774. DOI: https://doi.org/10.1002/rcm.8103

43. Lyu, J.; Kapolka, N.; Gumpper, R.; Alon, A.; Wang, L.; Jain, M. K.; Barros-Álvarez, X.; Sakamoto, K.; Kim, Y.; DiBerto, J.; Kim, K.; Glenn, I. S.; Tummino, T. A.; Huang, S.; Irwin, J. J.; Tarkhanova, O. O.; Moroz, Y.; Skiniotis, G.; Kruse, A. C.; Shoichet, B. K.; Roth, B. L., AlphaFold2 structures guide prospective ligand discovery. *Science* **2024,** *384*, (6702), eadn6354. DOI: http://doi.org/10.1126/science.adn6354

44. Mohanty, M.; Mohanty, P. S., Molecular docking in organic, inorganic, and hybrid systems: a tutorial review. *Monatshefte für Chemie - Chemical Monthly* **2023,** *154*, 683-707. DOI: https://doi.org/10.1007/s00706-023-03076-1

45. Du, J.; Gebicki, J. M., Proteins are major initial cell targets of hydroxyl free radicals. *The International Journal of Biochemistry & Cell Biology* **2004,** *36*, (11), 2334-2343. DOI: https://doi.org/10.1016/j.biocel.2004.05.012

46. Hohenberger, J.; Ray, K.; Meyer, K., The biology and chemistry of high-valent iron–oxo and iron–nitrido complexes. *Nature Communications* **2012,** *3*, (1), 720. DOI: https://doi.org/10.1038/ncomms1718

47. Priya, P. L.; Shanmughavel, P., A docking model of human ribonucleotide reductase with flavin and phenosafranine. *Bioinformation* **2009,** *4*, (3), 123-6. DOI: https://doi.org/10.6026/97320630004123

48. Basu, A.; Sinha, B. N., Understanding the molecular interactions of different radical scavengers with ribonucleotide reductase M2 (hRRM2) domain: opening the gates and gaining access. *Journal of Computer-Aided Molecular Design* **2012,** *26*, (7), 865-881. DOI: https://doi.org/10.1007/s10822-012-9581-y

49. Maryam, I.; Zeynab, K.; Asghar, D., Modeling and proposed molecular mechanism of hydroxyurea through docking and molecular dynamic simulation to curtail the action of ribonucleotide reductase. *Recent Patents on Anti-Cancer Drug Discovery* **2016,** *11*, (4), 461-468. DOI: https://doi.org/10.2174/1574892811666160926143534

50. Morris, G. M.; Huey, R.; Lindstrom, W.; Sanner, M. F.; Belew, R. K.; Goodsell, D. S.; Olson, A., AutoDock4 and AutoDockTools4: Automated docking with selective receptor flexibility. *Journal of Computational Chemistry* **2009,** *30*, (16), 2785-2791. DOI: https://doi.org/10.1002/jcc.21256

51. Holt, P. A.; Chaires, J. B.; Trent, J. O., Molecular docking of intercalators and groove-binders to nucleic acids using *Autodock* and *Surflex*. *Journal of Chemical Information and Modeling* **2008,** *48*, (8), 1602-1615. DOI: https://doi.org/10.1021/ci800063v

52. Pettersen, E. F.; Goddard, T. D.; Huang, C. C.; Couch, G. S.; Greenblatt, D. M.; Meng, E. C.; Ferrin, T. E., UCSF chimera—A visualization system for exploratory research and analysis. *Journal of Computational Chemistry* **2004,** *25*, (13), 1605-1612. DOI: https://doi.org/10.1002/jcc.20084

53. Huang, C. C.; Meng, E. C.; Morris, J. H.; Pettersen, E. F.; Ferrin, T. E., Enhancing UCSF Chimera through web services. *Nucleic Acids Research* **2014,** *42*, (W1), W478-W484. DOI: https://doi.org/10.1093/nar/gku377

54. Visualizer, D. S. *B. Discovery Studio Visualizer*, 16.1.0.15350; Dassault Systemes: an Diego,, 2016. https://discover.3ds.com/discovery-studio-visualizer-download

55. O'Boyle, N. M.; Banck, M.; James, C. A.; Morley, C.; Vandermeersch, T.; Hutchison, G. R., Open Babel: An open chemical toolbox. *Journal of Cheminformatics* **2011,** *3*, (1), 33. DOI: https://doi.org/10.1186/1758-2946-3-33

56. Neese, F.; Wennmohs, F.; Becker, U.; Riplinger, C., The ORCA quantum chemistry program package. *The Journal of Chemical Physics* **2020,** *152*, (22). DOI: https://doi.org/10.1063/5.0004608

57. Berman, H. M.; Westbrook, J.; Feng, Z.; Gilliland, G.; Bhat, T. N.; Weissig, H.; Shindyalov, I. N.; Bourne, P. E., The Protein Data Bank. *Nucleic Acids Research* **2000,** *28*, (1), 235-242. DOI: https://doi.org/10.1093/nar/28.1.235

58. Linstrom, P. NIST Chemistry WebBook, SRD 69. 2005. (accessed September 1^{6th}, 2024).

59. National Center for Biotechnology Information PubChem compound summary for CID 11963629, Iron(IV). 2024. (accessed September 28).

60. Bergwerf, H., MolView: an attempt to get the cloud into chemistry classrooms. In *ACS CHED CCCE Newsletter*, DivCHED CCCE: Committee on Computers in Chemical Education: 2015; Vol. 9, pp 1-9. https://confchem.ccce.divched.org/2015FallCCCENLP9

61. Morris, G. M.; Goodsell, D. S.; Halliday, R. S.; Huey, R.; Hart, W. E.; Belew, R. K.; Olson, A., Automated docking using a Lamarckian genetic algorithm and an empirical binding free energy function. *Journal of Computational Chemistry* **1998,** *19*, (14), 1639-1662. DOI: https://doi.org/10.1002/(SICI)1096-987X(19981115)19:14%3C1639::AID-JCC10%3E3.0.CO;2-B

62. Morris, G. M.; Goodsell, D. S.; Pique, M. E.; Lindstrom, W.; Huey, R.; Forli, S.; Hart, W. E.; Halliday, S.; Belew, R.; Olson, A. J. u. g. A., User Guide: Automated docking of flexible ligands to flexible receptors. In *AutoDock Version 4.2*, AutoDockTools: 2010. DOI: https://autodock.scripps.edu/wp-content/uploads/sites/56/2022/04/AutoDock3.0.5_UserGuide.pdf

63. Ahmad, I.; Singh, A. K.; Mohd, S.; Katari, S. K.; Nalamolu, R. M.; Ahmad, A.; Baothman, O. A.; Hosawi, S. A.; Altayeb, H.; Nadeem, M. S.; Ahmad, V., *In silico* insights into the arsenic binding mechanism deploying application of computational Biology-based toolsets. *ACS Omega* **2024,** *9*, (7), 7529-7544. DOI: https://doi.org/10.1021/acsomega.3c06313

64. Yang, J.; Roy, A.; Zhang, Y., Protein-ligand binding site recognition using complementary binding-specific substructure comparison and sequence profile alignment. *Bioinformatics (Oxford, England)* **2013,** *29*, (20), 2588-95. DOI: https://doi.org/10.1093/bioinformatics/btt447

65. Gao, M.; Skolnick, J., The distribution of ligand-binding pockets around protein-protein interfaces suggests a general mechanism for pocket formation. *Proceedings of the National Academy of Sciences* **2012,** *109*, (10), 3784-9. DOI: https://doi.org/10.1073/pnas.1117768109

66. Guo, Z.; Li, B.; Cheng, L. T.; Zhou, S.; McCammon, J. A.; Che, J., Identification of protein–ligand binding sites by the level-set variational implicit-solvent approach. *Journal of chemical theory and computation* **2015,** *11*, (2), 753-765. DOI: https://doi.org/10.1021/ct500867u

67. van der Reest, J.; Lilla, S.; Zheng, L.; Zanivan, S.; Gottlieb, E., Proteome-wide analysis of cysteine oxidation reveals metabolic sensitivity to redox stress. *Nature Communications* **2018,** *9*, (1), 1581. DOI: https://doi.org/10.1038/s41467-018-04003-3

68. Kisty, E. A.; Falco, J. A.; Weerapana, E., Redox proteomics combined with proximity labeling enables monitoring of localized cysteine oxidation in cells. *Cell chemical biology* **2023,** *30*, (3), 321-336.e6. DOI: https://doi.org/10.1016/j.chembiol.2023.02.006

69. Morales, F. J., Assessing the non-specific hydroxyl radical scavenging properties of melanoidins in a Fenton-type reaction system. *Analytica Chimica Acta* **2005,** *534*, (1), 171-176. DOI: https://doi.org/10.1016/j.aca.2004.11.028

70. Guptasarma, P.; Balasubramanian, D.; Matsugo, S.; Saito, I., Hydroxyl radical mediated damage to proteins, with special reference to the crystallins. *Biochemistry* **1992,** *31*, (17), 4296-4303. DOI: https://doi.org/10.1021/bi00132a021

71. Davies, M. J., The oxidative environment and protein damage. *Biochimica et Biophysica Acta (BBA) - Proteins and Proteomics* **2005,** *1703*, (2), 93-109. DOI: https://doi.org/10.1016/j.bbapap.2004.08.007

72. O'Dowd, K.; Pillai, S. C., Photo-Fenton disinfection at near neutral pH: Process, parameter optimization and recent advances. *Journal of Environmental Chemical Engineering* **2020,** *8*, (5), 104063. DOI: https://doi.org/10.1016/j.jece.2020.104063

73. Park, E. J.; Kim, M. S.; Lee, C., Aqueous Fe(IV) and Cu(III) species from Fenton (-like) reactions: Chemistry and environmental applications. *Water Research* **2025**, 124169. DOI: http://doi.org/10.1016/j.watres.2025.124169

74. Koppenol, W. H., Ferryl for real. The Fenton reaction near neutral pH. *Dalton Transactions* **2022,** *51*, (45), 17496-17502. DOI: https://doi.org/10.1039/D2DT03168J

75. Ensing, B.; Buda, F.; Blöchl, P. E.; Baerends, E. J., A Car–Parrinello study of the formation of oxidizing intermediates from Fenton's reagent in aqueous solution. *Physical Chemistry Chemical Physics* **2002,** *4*, (15), 3619-3627. DOI: https://doi.org/10.1039/B201864K

76. Garrido Ruiz, D.; Sandoval-Perez, A.; Rangarajan, A. V.; Gunderson, E. L.; Jacobson, M. P., Cysteine oxidation in proteins: Structure, biophysics, and simulation. *Biochemistry* **2022,** *61*, (20), 2165-2176. DOI: https://doi.org/10.1021/acs.biochem.2c00349

77. Valegård, K.; Liljas, L.; Fridborg, K.; Unge, T., The three-dimensional structure of the bacterial virus MS2. *Nature* **1990,** *345*, (6270), 36-41. DOI: https://doi.org/10.1038/345036a0

78. Bursch, M.; Mewes, J. M.; Hansen, A.; Grimme, S., Best-practice DFT protocols for basic molecular computational chemistry. *Angewandte Chemie* **2022,** *134*, (42), e202205735. DOI: https://doi.org/10.1002/ange.202205735

79. Rittle, J.; Green, M. T., Cytochrome P450 Compound I: Capture, Characterization, and C-H Bond Activation Kinetics. *Science* **2010,** *330*, (6006), 933-937. DOI: https://doi.org/10.1126/science.1193478

80. Decker, A.; Rohde, J. U.; Klinker, E. J.; Wong, S. D.; Que, L., Jr.; Solomon, E. I., Spectroscopic and quantum chemical studies on low-spin Fe^{IV}=O complexes: Fe-O bonding and its contributions to reactivity. *Journal of the American Chemical Society* **2007,** *129*, (51), 15983-96. DOI: https://doi.org/10.1021/ja074900s

81. Löwdin, P. O., On the non-orthogonality problem connected with the use of atomic wave functions in the theory of molecules and crystals. *The Journal of Chemical Physics* **1950,** *18*, (3), 365-375. DOI: https://doi.org/10.1063/1.1747632

82. Kim, K.; Jothikumar, N.; Sen, A.; Murphy, J. L.; Chellam, S., Removal and inactivation of an enveloped virus surrogate by iron conventional coagulation and electrocoagulation. *Environmental Science & Technology* **2021,** *55*, (4), 2674-2683. DOI: http://doi.org/10.1021/acs.est.0c07697

83. Nilling, J. J.; Verma, A.; Singh, A., Precipitation of arsenic-bearing solids as a secondary control on arsenic speciation in groundwater: Evidence from field study and geochemical analysis. *Geochimica et Cosmochimica Acta* **2022,** *333*, 308-332. DOI: https://doi.org/10.1016/j.gca.2022.07.017

84. Gustafsson, J. P., Visual Minteq 3.0 user guide. *KTH, Department of Land and Water Recources, Stockholm, Sweden* **2011**. https://www.labxing.com/files/lab_data/1750-1688022974-3V73rhX1.pdf

85. Bethke, C. M.; Farrell, B.; Yeakel, S., The Geochemist's Workbench: Community Edition. *Illinois: Aqueous Solutions, LLC Campaign* **2023**. https://www.gwb.com/pdf/GWB/GWBessentials.pdf

86. Pham, A. N.; Rose, A. L.; Feitz, A. J.; Waite, T. D., Kinetics of Fe(III) precipitation in aqueous solutions at pH 6.0–9.5 and 25°C. *Geochimica et Cosmochimica Acta* **2006,** *70*, (3), 640-650. DOI: https://doi.org/10.1016/j.gca.2005.10.018

87. Grundl, T.; Delwiche, J., Kinetics of ferric oxyhydroxide precipitation. *Journal of Contaminant Hydrology* **1993,** *14*, (1), 71-87. DOI: https://doi.org/10.1016/0169-7722(93)90042-Q

88. He, X.; Flynn, E. D.; Catalano, J. G.; Giammar, D. E., Selenium(VI) removal by continuous flow-through iron electrocoagulation: Effects of operating conditions and stability of selenium in residual solids. *Environmental Science & Technology* **2025,** *59*, (10), 5359-5369. DOI: https://doi.org/10.1021/acs.est.4c12305

89. Yuan, Y.; Mehrotra, M.; He, X.; Flynn, E. D.; Catalano, J. G.; Giammar, D. E., Advancing selenium(VI) removal by iron electrocoagulation: Roles of water chemistry and operating conditions. *ACS ES&T Engineering* **2025,** *5*, (7), 1821-1830. DOI: https://doi.org/10.1021/acsestengg.5c00068

90. Benjamin, M. M., *Water chemistry*. Second ed.; Waveland Press: USA, 2014. https://books.google.com/books/about/Water_Chemistry.html?id=UD_hoQEACAAJ

91. Stumm, W.; Morgan, J. J., *Aquatic chemistry: Chemical equilibria and rates in natural waters* Third ed.; John Wiley & Sons, Inc.: USA, 1996.

92. Lakshmanan, D.; Clifford, D. A.; Samanta, G., Ferrous and ferric ion generation during iron electrocoagulation. *Environmental Science & Technology* **2009,** *43*, (10), 3853-3859. DOI: https://doi.org/10.1021/es8036669

93. Chaurand, P.; Schriver, K. E.; Caprioli, R. M., Instrument design and characterization for high resolution MALDI-MS imaging of tissue sections. *Journal of Mass Spectrometry* **2007,** *42*, (4), 476-489. DOI: https://doi.org/10.1002/jms.1180

94. Cobo, F., Application of MALDI-TOF mass spectrometry in clinical virology: a review. *Open Virology* **2013,** *7*, 84-90. DOI: https://doi.org/10.2174/1874357920130927003

95. Satoh, T.; Takei, M.; Uematsu, F., Development of a peak extraction method using the high-resolution matrix-assisted laser desorption/ionization time-of-flight mass spectrometry and machine learning techniques: Analysis of peak shapes. *apid Communications in Mass Spectrometry* **2022,** *36*, (6), e9235. DOI: https://doi.org/10.1002/rcm.9235

96. Ng, E. W. Y.; Wong, M. Y. M.; Poon, T. C. W., Advances in MALDI mass spectrometry in clinical diagnostic applications. In *Chemical Diagnostics: From Bench to Bedside*, L.S. Tang, N.; Poon, T., Eds. Springer Berlin Heidelberg: Berlin, Heidelberg, 2014; pp 139-175. DOI: https://doi.org/10.1007/128_2012_413

97. Zhu, X.; Xu, T.; Peng, C.; Wu, S., Advances in MALDI mass spectrometry imaging single cell and tissues. *Frontiers in Chemistry* **2022,** *Volume 9 - 2021*. DOI: https://doi.org/10.3389/fchem.2021.782432

98. Slysz, G. W.; Lewis, D. F.; Schriemer, D. C., Detection and identification of sub-nanogram levels of protein in a nanoLC-trypsin-MS system. *Journal of Proteome Research* **2006,** *5*, (8), 1959-1966. DOI: https://doi.org/10.1021/pr060142d

99. Schneider, G.; Garbett, N. C., Sample processing considerations for protein stability Studies of low concentration biofluid samples using differential scanning calorimetry. *Protein and peptide letters* **2022,** *29*, (6), 485-495. DOI: https://doi.org/10.2174/0929866529666220416164305

100. Stathopulos, P. B.; Scholz, G. A.; Hwang, Y.-M.; Rumfeldt, J. A. O.; Lepock, J. R.; Meiering, E. M., Sonication of proteins causes formation of aggregates that resemble amyloid. *Protein Science* **2004,** *13*, (11), 3017-3027. DOI: https://doi.org/10.1110/ps.04831804

101. Zeegers-Huyskens, T.; Huyskens, P., Intermolecular forces. In *Intermolecular forces: An introduction to modern methods and results*, Zeegers-Huyskens, T.; Huyskens, P., Eds. Springer: Berlin, Heidelberg 1991; pp 1-30. DOI: https://doi.org/10.1002/ange.19921041161

102. Kitchen, D. B.; Decornez, H.; Furr, J. R.; Bajorath, J., Docking and scoring in virtual screening for drug discovery: methods and applications. *Nature Reviews Drug Discovery* **2004,** *3*, (11), 935-949. DOI: https://doi.org/10.1038/nrd1549

103. Volkenstein, M. V., Coding of polar and non-polar amino-acids. *Nature* **1965,** *207*, (4994), 294-295. DOI: https://doi.org/10.1038/207294a0

104. Ni, C. Z.; Syed, R.; Kodandapani, R.; Wickersham, J.; Peabody, D. S.; Ely, K. R., Crystal structure of the MS2 coat protein dimer: implications for RNA binding and virus assembly. *Structure* **1995,** *3*, (3), 255-263. DOI: https://doi.org/10.1016/S0969-2126(01)00156-3

105. Rose, G. D.; Geselowitz, A. R.; Lesser, G. J.; Lee, R. H.; Zehfus, M. H., Hydrophobicity of Amino Acid Residues in Globular Proteins. *Science* **1985,** *229*, (4716), 834-838. DOI: https://doi.org/10.1126/science.4023714

106. Finley, E. L.; Dillon, J.; Crouch, R. K.; Schey, K., Identification of tryptophan oxidation products in bovine α-crystallin. *Protein Science* **1998,** *7*, (11), 2391-2397. DOI: https://doi.org/10.1002/pro.5560071116

107. Meng, R.; Jiang, M.; Cui, Z.; Chang, J. Y.; Yang, K.; Jakana, J.; Yu, X.; Wang, Z.; Hu, B.; Zhang, J., Structural basis for the adsorption of a single-stranded RNA bacteriophage. *Nature Communications* **2019,** *10*, (1), 3130. DOI: https://doi.org/10.1038/s41467-019-11126-8

108. Barth, A., Infrared spectroscopy of proteins. *Biochimica et Biophysica Acta (BBA) - Bioenergetics* **2007,** *1767*, (9), 1073-1101. DOI: https://doi.org/10.1016/j.bbabio.2007.06.004

109. Jackson, M.; Mantsch, H. H., The use and misuse of FTIR spectroscopy in the determination of protein structure. *Critical Reviews in Biochemistry and Molecular Biology* **1995,** *30*, (2), 95-120. DOI: https://doi.org/10.3109/10409239509085140

110. Stuart, B. H., *Infrared spectroscopy: fundamentals and applications*. John Wiley & Sons: 2004. DOI: https://doi.org/10.1002/0470011149

111. Movasaghi, Z.; Rehman, S.; ur Rehman, D. I., Fourier transform infrared (FTIR) spectroscopy of biological tissues. *Applied Spectroscopy Reviews* **2008,** *43*, (2), 134-179. DOI: https://doi.org/10.1080/05704920701829043

112. Duan, Y.; Jiang, W.; Sedlak, D. L., Surface processes control the fate of reactive oxidants generated by electrochemical activation of hydrogen peroxide on stainless-steel electrodes. *Environmental Science & Technology* **2023,** *57*, (47), 18680-18689. DOI: https://doi.org/10.1021/acs.est.2c08404

113. Lin, S. S.; Gurol, M. D., Catalytic decomposition of hydrogen peroxide on iron Oxide: Kinetics, mechanism, and implications. *Environmental Science & Technology* **1998,** *32*, (10), 1417-1423. DOI: https://doi.org/10.1021/es970648k

114. De Laat, J.; Gallard, H., Catalytic decomposition of hydrogen peroxide by Fe(III) in homogeneous aqueous solution: Mechanism and kinetic modeling. *Environmental Science & Technology* **1999,** *33*, (16), 2726-2732. DOI: https://doi.org/10.1021/es981171v

115. Yang, Z. C.; Wang, W. L.; Jing, Z. B.; Jiang, Y. Q.; Zhang, H. Q.; Lee, M. Y.; Peng, L.; Wu, Q. Y., Ozone, hydrogen peroxide, and peroxymonosulfate disinfection of MS2 coliphage in water. *Environmental Science: Processes & Impacts* **2024,** *26*, (5), 824-831. DOI: https://doi.org/10.1039/D3EM00527E

116. Hall, R. M.; Sobsey, M. D., Inactivation of Hepatitis A virus and MS2 by ozone and ozone-hydrogen peroxide in buffered water. *Water Science and Technology* **1993,** *27*, (3-4), 371-378. DOI: https://doi.org/10.2166/wst.1993.0377

117. Silva, K. J. S.; Sabogal-Paz, L. P., A 10-year critical review on hydrogen peroxide as a disinfectant: could it be an alternative for household water treatment? *Water Supply* **2022,** *22*, (12), 8527-8539. DOI: https://doi.org/10.2166/ws.2022.384

118. Szczuka, A.; Berglund-Brown, J. P.; MacDonald, J. A.; Mitch, W. A., Control of sulfides and coliphage MS2 using hydrogen peroxide and UV disinfection for non-potable reuse of pilot-scale anaerobic membrane bioreactor effluent. *Water Research X* **2021,** *11*, 100097. DOI: https://doi.org/10.1016/j.wroa.2021.100097

119. Wang, Y.; Liu, L.; Yang, X.; Suib, S. L.; Qiu, G., Removal of As(V) from wastewaters using magnetic iron oxides formed by zero-valent iron electrocoagulation. *Journal of Environmental Management* **2022,** *307*, 114519. DOI: https://doi.org/10.1016/j.jenvman.2022.114519

120. Dubrawski, K. L.; Mohseni, M., In-situ identification of iron electrocoagulation speciation and application for natural organic matter (NOM) removal. *Water Research* **2013,** *47*, (14), 5371-5380. DOI: https://doi.org/10.1016/j.watres.2013.06.021

121. Guo, Y.; Shi, J.; Sharma, E.; Gao, S.; Zhou, X.; Liu, Y.; Sivakumar, M.; Jiang, G., Fate of coronaviruses during the wastewater coagulation with ferric chloride. *ACS ES&T Water* **2023,** *3*, (10), 3206-3214. DOI: https://doi.org/10.1021/acsestwater.3c00112

122. Haas, C. N.; Joffe, J.; Anmangandla, U.; Jacangelo, J. G.; Heath, M., Water quality and disinfection kinetics. *Journal (American Water Works Association)* **1996,** *88*, (3), 95-103. DOI: https://www.jstor.org/stable/41295474

123. Nelson, K. L.; Boehm, A. B.; Davies-Colley, R. J.; Dodd, M. C.; Kohn, T.; Linden, K. G.; Liu, Y.; Maraccini, P. A.; McNeill, K.; Mitch, W. A.; Nguyen, T. H.; Parker, K. M.; Rodriguez, R. A.; Sassoubre, L. M.; Silverman, A. I.; Wigginton, K. R.; Zepp, R. G., Sunlight-mediated inactivation of health-relevant microorganisms in water: a review of mechanisms and modeling approaches. *Environmental Science: Processes & Impacts* **2018,** *20*, (8), 1089-1122. DOI: https://doi.org/10.1039/C8EM00047F

124. Dong, M. M.; Mezyk, S. P.; Rosario-Ortiz, F. L., Reactivity of effluent organic matter (EfOM) with hydroxyl radical as a function of molecular weight. *Environmental Science & Technology* **2010,** *44*, (15), 5714-5720. DOI: https://doi.org/10.1021/es1004736

125. Appiani, E.; Page, S. E.; McNeill, K., On the use of hydroxyl radical kinetics to assess the number-average molecular weight of dissolved organic matter. *Environmental Science & Technology* **2014,** *48*, (20), 11794-11802. DOI: https://doi.org/10.1021/es5021873

126. Lee, E.; Glover, C. M.; Rosario-Ortiz, F. L., Photochemical formation of hydroxyl radical from effluent organic matter: Role of composition. *Environmental Science & Technology* **2013,** *47*, (21), 12073-12080. DOI: https://doi.org/10.1021/es402491t

127. Lindsey, M. E.; Tarr, M. A., Inhibition of hydroxyl radical reaction with aromatics by dissolved natural organic matter. *Environmental Science & Technology* **2000,** *34*, (3), 444-449. DOI: https://doi.org/10.1021/es990457c

128. Zhang, S.; Hedtke, T.; Zhu, Q.; Sun, M.; Weon, S.; Zhao, Y.; Stavitski, E.; Elimelech, M.; Kim, J. H., Membrane-confined iron oxychloride nanocatalysts for highly efficient heterogeneous fenton water treatment. *Environmental Science & Technology* **2021,** *55*, (13), 9266-9275. DOI: https://doi.org/10.1021/acs.est.1c01391

129. Wan, Z.; Chae, S. H.; Meese, A. F.; Nwokonkwo, O.; Arrazolo, L.; Yip, K. L.; Ma, X.; Liu, S.; Muhich, C.; Wang, D.; Wei, H.; Kim, J. H., Overcoming the reactivity-stability challenge in water treatment catalyst through spatial confinement. *Nature Communications* **2025,** *16*, (1), 9672. DOI: https://doi.org/10.1038/s41467-025-64684-5

130. Rose, A. L.; Waite, T. D., Effect of dissolved natural organic matter on the kinetics of ferrous iron oxygenation in seawater. *Environmental Science & Technology* **2003,** *37*, (21), 4877-4886. DOI: https://doi.org/10.1021/es034152g

131. McBeath, S. T.; Mohseni, M.; Wilkinson, D. P., Pilot-scale iron electrocoagulation treatment for natural organic matter removal. *Environmental Technology* **2020,** *41*, (5), 577-585. DOI: https://doi.org/10.1080/09593330.2018.1505965

132. Nickabadi, S.; Golmohammadi, B.; Hadavi, M., Enhanced organic matter removal and fouling mitigation in seawater desalination using electrocoagulation pretreatment using ZnO coated Fe electrodes. *Scientific Reports* **2025,** *15*, (1), 8256. DOI: https://doi.org/10.1038/s41598-025-93220-0

133. Hand, S.; Cusick, R. D., Electrochemical disinfection in water and wastewater treatment: Identifying impacts of water quality and operating conditions on performance. *Environmental Science & Technology* **2021,** *55*, (6), 3470-3482. DOI: https://doi.org/10.1021/acs.est.0c06254

134. Kabdaşlı, I.; Arslan-Alaton, I.; Ölmez-Hancı, T.; Tünay, O., Electrocoagulation applications for industrial wastewaters: a critical review. *Environmental Technology Reviews* **2012,** *1*, (1), 2-45. DOI: https://doi.org/10.1080/21622515.2012.715390

135. Amrose, S. E.; Bandaru, S. R. S.; Delaire, C.; van Genuchten, C. M.; Dutta, A.; DebSarkar, A.; Orr, C.; Roy, J.; Das, A.; Gadgil, A. J., Electro-chemical arsenic remediation: Field trials in West Bengal. *Science of The Total Environment* **2014,** *488-489*, 539-546. DOI: https://doi.org/10.1016/j.scitotenv.2013.11.074

136. Thurston-Enriquez, J. A.; Haas, C. N.; Jacangelo, J.; Gerba, C. P., Chlorine Inactivation of Adenovirus Type 40 and Feline Calicivirus. *Applied and Environmental Microbiology* **2003,** *69*, (7), 3979-3985. DOI: https://doi.org/10.1128/AEM.69.7.3979-3985.2003

137. Sellaoui, L.; Badawi, M.; Monari, A.; Tatarchuk, T.; Jemli, S.; Luiz Dotto, G.; Bonilla-Petriciolet, A.; Chen, Z., Make it clean, make it safe: A review on virus elimination via adsorption. *Chemical Engineering Journal* **2021,** *412*, 128682. DOI: https://doi.org/10.1016/j.cej.2021.128682

138. Sillanpää, M.; Shestakova, M., *Electrochemical water treatment methods: Fundamentals, methods and full scale applications*. Butterworth-Heinemann, Elsevier: United Kingdom and United States, 2017. DOI: https://dx.doi.org/10.1016/B978-0-12-811462-9.00001-3

139. Duan, J.; Gregory, J., Coagulation by hydrolysing metal salts. *Advances in Colloid and Interface Science* **2003,** *100-102*, 475-502. DOI: https://doi.org/10.1016/S0001-8686(02)00067-2

140. Grebel, J. E.; Pignatello, J. J.; Mitch, W. A., Effect of halide ions and carbonates on organic contaminant degradation by hydroxyl radical-based advanced oxidation processes in saline waters. *Environmental Science & Technology* **2010,** *44*, (17), 6822-6828. DOI: https://doi.org/10.1021/es1010225

141. Yang, Y.; Jiang, J.; Lu, X.; Ma, J.; Liu, Y., Production of sulfate radical and hydroxyl radical by reaction of ozone with peroxymonosulfate: A novel advanced oxidation process. *Environmental Science & Technology* **2015,** *49*, (12), 7330-7339. DOI: https://doi.org/10.1021/es506362e

142. Lian, L.; Yao, B.; Hou, S.; Fang, J.; Yan, S.; Song, W., Kinetic study of hydroxyl and sulfate radical-mediated oxidation of pharmaceuticals in wastewater effluents. *Environmental Science & Technology* **2017,** *51*, (5), 2954-2962. DOI: 10.1021/acs.est.6b05536

143. Zhu, C.; Ye, Y., Reactivity of viral proteins with free chlorine: Structural insights and implications for virus inactivation. *Environmental Science & Technology* **2025,** *59*, (32), 17188-17197. DOI: https://doi.org/10.1021/acs.est.5c01689

144. Bastin, G.; Loison, P.; Vernex-Loset, L.; Dupire, F.; Challant, J.; Majou, D.; Boudaud, N.; Krier, G.; Gantzer, C., Structural organizations of Qβ and MS2 phages affect capsid protein modifications by oxidants hypochlorous acid and peroxynitrite. **2020,** *Volume 11 - 2020*. DOI: https://doi.org/10.3389/fmicb.2020.01157
